# Cobalta‐Electrocatalyzed C−H Activation in Biomass‐Derived Glycerol: Powered by Renewable Wind and Solar Energy

**DOI:** 10.1002/cssc.202000057

**Published:** 2020-01-30

**Authors:** Tjark H. Meyer, Gleb A. Chesnokov, Lutz Ackermann

**Affiliations:** ^1^ Institut für Organische und Biomolekulare Chemie Georg-August-Universität Göttingen Tammannstraße 2 37077 Göttingen Germany

**Keywords:** biomass, C−H activation, cobalt, electrochemistry, renewable energy, sustainability

## Abstract

Aqueous glycerol was identified as a renewable reaction medium for metalla‐electrocatalyzed C−H activation powered by sustainable energy sources. The renewable solvent was employed for cobalt‐catalyzed C−H/N−H functionalizations under mild conditions. The cobalta‐electrocatalysis manifold occurred with high levels of chemo‐ and positional selectivity and allowed for electrochemical C−H activations with broad substrate scope. The resource economy of this strategy was considerably substantiated by the direct use of renewable solar and wind energy.

## Graphical abstract

**Renewing C−H activation**: Renewable resources prove amenable to enable cobalta‐electrocatalyzed C−H activations in aqueous glycerol, empowered by solar or wind energy. The cobalta‐electrocatalysis manifold occurs with high levels of chemo‐ and positional selectivity and allows for electrochemical C−H activations with broad substrate scope.

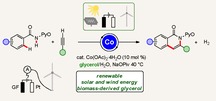

During the last decade, the development of efficient electrocatalysis for the interconversion of renewable energies, such as wind and solar energy, into value‐added products has attracted significant attention.^1^ A promising approach to convert the sustainable energy into chemical energy is the conversion of small molecules (e.g., by water oxidation) into alternative fuels.^2^ The splitting of water by electrolysis is separated in two half‐cell reactions, namely the cathodic hydrogen evolution reaction (HER) and the anodic oxygen evolution reaction (OER).^3^ In case of the OER, however, kinetic limitations result in a high‐overpotential,^4^ which renders the overall process highly energy consuming and as of yet too costly for large‐scale applications. Also, the resulting oxygen is of minor value, which lowers the overall economic footprint.^5^ Of greater interest would be the production of value‐added products on the anodic half‐cell reaction.^6^

In the meantime, C−H activation^7^ has emerged as a transformative tool in molecular sciences by its applications towards material sciences,^8^ drug discovery,^9^ and complex bioactive natural product synthesis.^10^ Despite indisputable advances, in the scenario of oxidative C−H functionalization stoichiometric amounts of metal‐based, often costly, and toxic oxidants are commonly required. Therefore, the merger of electrosynthesis^11^ and oxidative C−H activation has recently enabled the use of electricity as the terminal oxidant, obviating the use of chemical redox reagents.^12^ In this context, our group as well as Lei and co‐workers recently reported on a cobalta‐electrocatalyzed^13^ C−H/N−H alkyne annulation at ambient temperature.^14^ An energy‐relevant, beneficial asset of this strategy was represented by the HER as the cathodic half reaction. Nevertheless, most of the metal‐catalyzed C−H activations rely on the use of harmful, fossil‐derived, and noxious solvents, such as halogenated solvents.^15^ To address these major challenges, continuous efforts were directed towards the identification of less hazardous, environmentally benign solvents for the inherently sustainable C−H activation approach.^16^ Competing undesired cobalt‐catalyzed methoxygenation reactions^17^ were observed when methanol was used as a green solvent. In stark contrast, glycerol has arguably thus far not been employed as a biomass‐derived reaction medium in C−H functionalization processes. However, it would serve as an ideal candidate in sustainable electrochemical transformations because of its high conductivity, obviating the use of additional conducting salts.^18^ Biocompatible glycerol can be classified as a non‐flammable solvent, and it is produced on large scale as the waste product of biodiesel production.^19^ Importantly, to improve the overall resource economy^20^ of the strategy, we decided to power the desired oxidative transformation with renewable solar and wind energy (Figure 1).^21^

**Figure 1:**
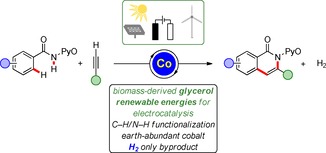
Electrochemical cobalt‐catalyzed C−H activation in glycerol empowered by solar and wind energy.

We initiated our studies by probing various reaction conditions for the envisioned electrochemical C−H transformation of substrate **1 a** with alkyne **2 a** in biomass‐derived glycerol (Table 1 and Table S1 in the Supporting Information).^22^ Thus, the desired product **3 aa** was obtained in excellent yield when 10 mol % Co(OAc)_2_⋅4 H_2_O was used as the catalyst (Table 1, entry 1). Here, a user‐friendly and cost‐efficient setup in an undivided cell, with graphite felt (GF) and platinum plate (Pt) as anode and cathode material, respectively, proved viable. The robust cobalt catalyst was operative under protic conditions and fully tolerant of H_2_O and glycerol. The addition of H_2_O was found to be essential because a higher concentration of glycerol led to a dramatic decrease in the product yield (entry 2). Sodium pivalate proved to be the optimal additive (entries 3 and 4). It is particularly noteworthy that commonly used toxic solvents as well as alternative renewable solvents, other than glycerol, were less efficient (entries 5–12 and Supporting Information). This can be explained by the high dielectric constant of glycerol (*ϵ*=42.5 at 25 °C),^23^ which renders it a suitable solvent for organic electrochemistry, and further addition of expensive conducting electrolytes can thereby be avoided (entries 9–11). Owing to the high viscosity of glycerol, the reaction temperature needed to be slightly adjusted to 40 °C to deliver the desired product **3 aa** in high yield,^22^ following the Stokes–Einstein equation. Notably, the reaction even proceeded efficiently with lower loadings of the cobalt(II) catalyst (entry 13), whereas the use of cobalt(III) salts yielded product **3 aa** in similar yields (entry 14). Control experiments verified the necessity of the cobalt catalyst and of the electric current (entries 15 and 16). Furthermore, cyclic voltammetry clearly indicated a cobalt oxidation, prior to substrate or solvent degradation.^22^, ^24^

**Table 1:** Optimization of the cobalta‐electrocatalyzed alkyne annulation.^[a]^

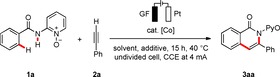				
Entry	[Co]	Solvent	Additive	Yield^[b]^ [%]
1	Co(OAc)_2_⋅4 H_2_O	glycerol/H_2_O (1:1)	NaOPiv	96 (92)
2	Co(OAc)_2_⋅4 H_2_O	glycerol	NaOPiv	24^[c]^
3	Co(OAc)_2_⋅4 H_2_O	glycerol/H_2_O (1:1)	HOPiv	14
4	Co(OAc)_2_⋅4 H_2_O	glycerol/H_2_O (1:1)	Na_2_CO_3_	29^[c]^
5	Co(OAc)_2_⋅4 H_2_O	2‐MeTHF/H_2_O (1:1)	NaOPiv	65^[c]^
6	Co(OAc)_2_⋅4 H_2_O	furfuryl alcohol/H_2_O (1:1)	NaOPiv	61^[c]^
7	Co(OAc)_2_⋅4 H_2_O	GVL/H_2_O (1:1)	NaOPiv	56^[c]^
8	Co(OAc)_2_⋅4 H_2_O	MeOH	NaOPiv	82 (78)^[d]^
9	Co(OAc)_2_⋅4 H_2_O	THF	NaOPiv	18^[e]^
10	Co(OAc)_2_⋅4 H_2_O	MeCN	NaOPiv	51^[e]^
11	Co(OAc)_2_⋅4 H_2_O	DCE	NaOPiv	43^[e]^
12	Co(OAc)_2_⋅4 H_2_O	TFE	NaOPiv	87^[f]^
13	Co(OAc)_2_⋅4 H_2_O	glycerol/H_2_O (1:1)	NaOPiv	73^[g]^
14	Co(OAc)_3_	glycerol/H_2_O (1:1)	NaOPiv	92 (91)
15	Co(OAc)_2_⋅4 H_2_O	glycerol/H_2_O (1:1)	NaOPiv	–^[h]^
16	–	glycerol/H_2_O (1:1)	NaOPiv	–

[a] Reaction conditions: Undivided cell, **1 a** (0.5 mmol), **2** (1.0 mmol), [Co] (10 mol %), additive (2.0 equiv.), solvent (5 mL), 40 °C, constant‐current electrolysis (CCE) at 4 mA, 15 h, graphite felt anode, Pt‐plate cathode, conversion measured by ^1^H NMR spectroscopy with 1,3,5‐trimethoxybenzene as internal standard. [b] Isolated yield in parentheses. [c] [Co] (20 mol %). [d] 9 % yield of oxygenated side product. [e] Addition of LiClO_4_ (1.0 equiv.). [f] 5 % yield of oxygenated side product. [g] [Co] (5.0 mol %). [h] No electricity. GVL=γ‐valerolactone; DCE=1,2‐dichloroethane; TFE=2,2,2‐trifluoroethanol.

With the optimized reaction conditions in hand, we probed its versatility in the C−H/N−H functionalizations of benzamides **1** in biomass‐derived glycerol (Scheme 1 a). Thus, the robust cobalta‐electrocatalysis enabled the efficient C−H activation of differently decorated amides **1** in aqueous glycerol. Notably, various functional groups, such as ether and fluoro groups, were fully tolerated. Owing to the lower solubility of amide **1 b**, the reaction was performed under sonification19c to furnish improved yields. Further, iodoarene (**1 f**) was smoothly converted to the desired product **3 fa**, albeit with slightly diminished yield. The positional selectivity for *meta*‐substituted arene **3 ja** in the electrochemical C−H activation was controlled by repulsive steric interactions. The use of glycerol/H_2_O as a solvent mixture extended the scope to thioethers **3 ga**, and even furan **3 ka** was converted likewise (Scheme 1 b). Finally, the robustness of the sustainable cobalta‐electrocatalyzed C−H/N−H functionalization could further be illustrated by the step‐economical synthesis of pyridones **3 la** and **3 ma** through alkenylic C−H activation.

**Scheme 1:**
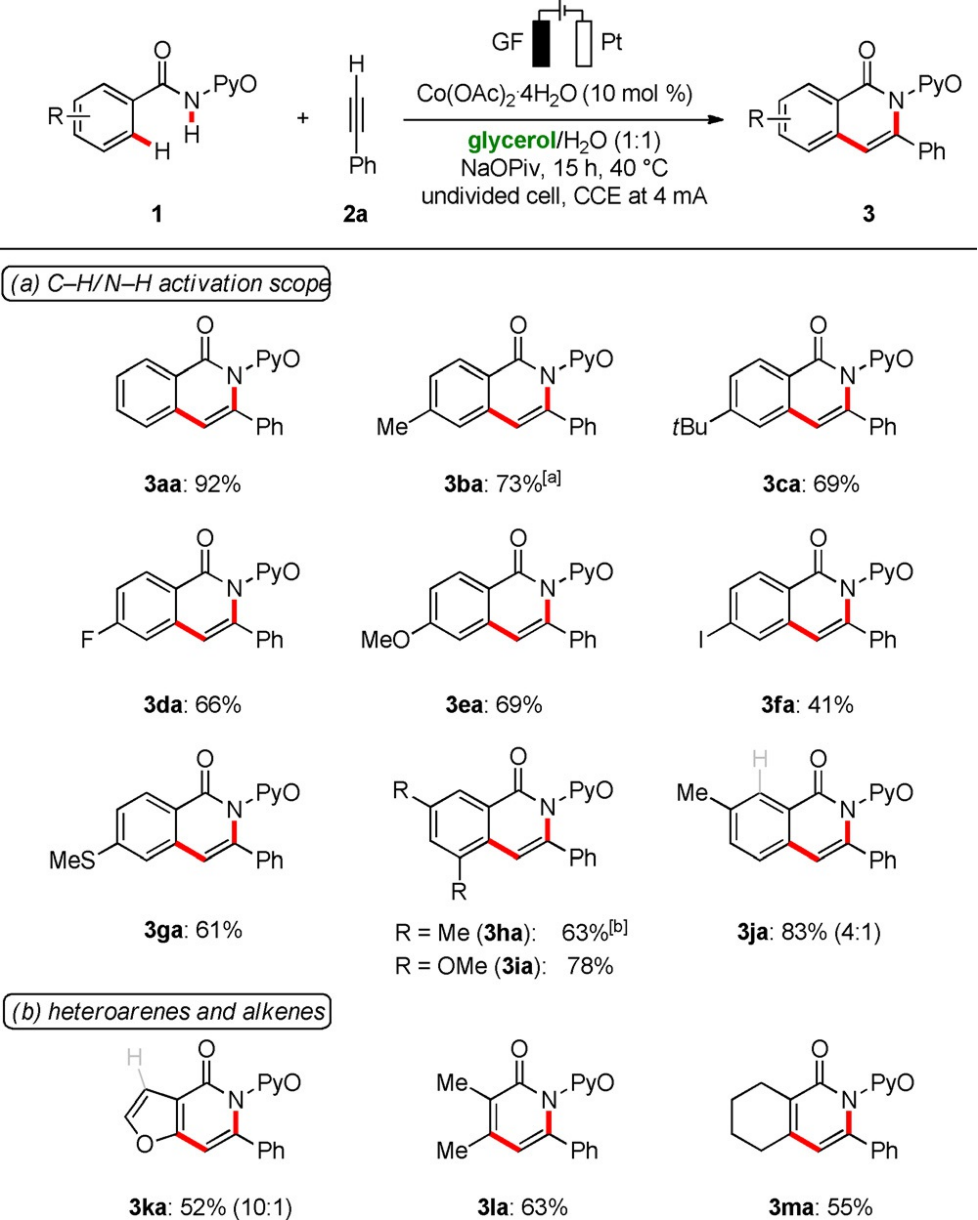
Versatility of the C−H activation in glycerol. [a] Under sonification, Co(OAc)_3_ (10 mol %).^22^ [b] Co(OAc)_3_ (10 mol %).

The robustness of the broadly applicable electrochemical C−H/N−H activation in biomass‐derived glycerol was further probed with substituted alkynes **2** (Scheme 2). Hence, the generality of the green cobalta‐electrocatalytic C−H activation was reflected by the effective annulation of a wealth of alkynes **2**, fully tolerant of valuable functional groups, including cyclopropyl and ether groups as well as sensitive alkyl chloride and nitrile substituents.

**Scheme 2:**
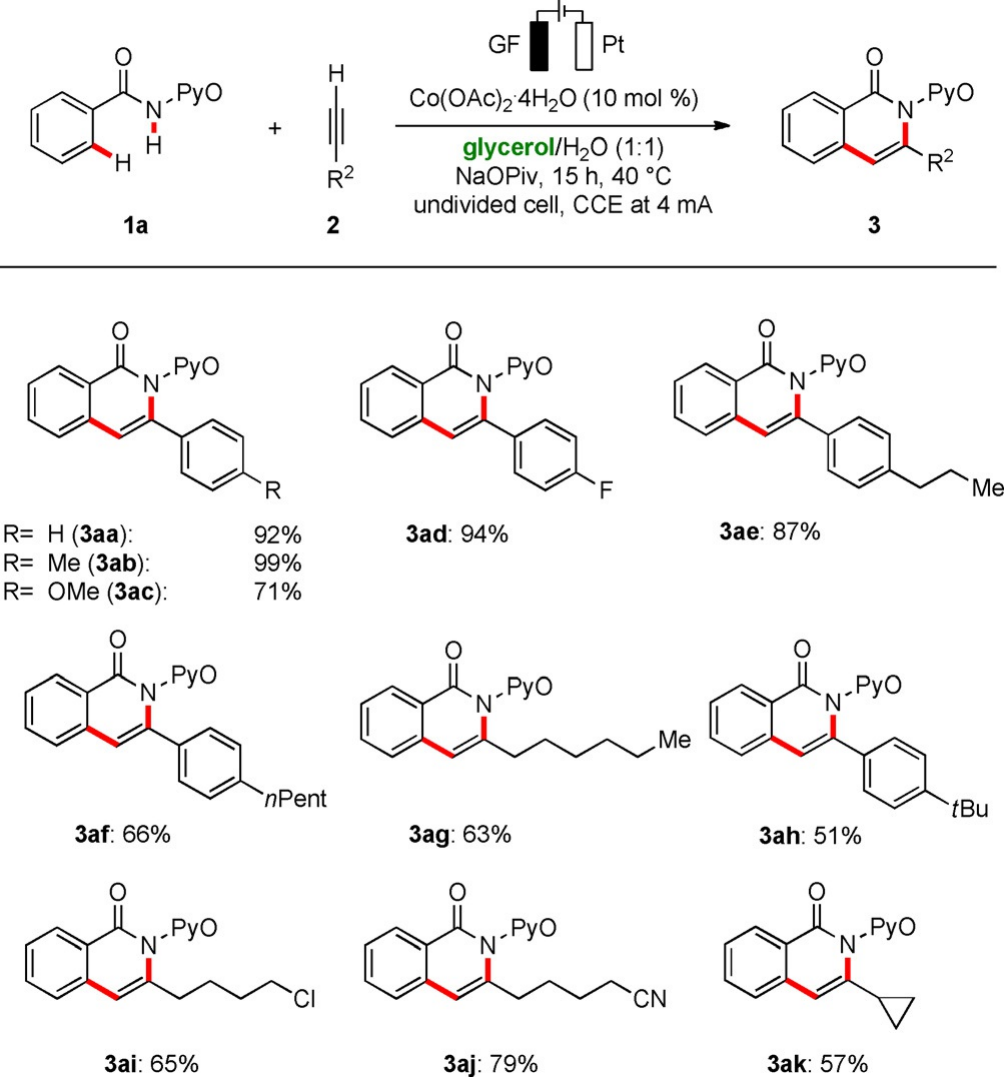
Cobalta‐electrocatalyzed C−H annulation of alkynes **2**.

Moreover, we were pleased to find that the cobalta‐electrocatalyzed C−H/N−H activation in biomass‐derived glycerol was not limited to alkynes **2**. Indeed, oxidative C−H annulation was also accomplished with the challenging allene **4**, using a slightly lower constant current of 2 mA (Scheme 3).

**Scheme 3:**
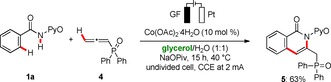
Electrochemical C−H annulation with allene **4** in glycerol/H_2_O.

Finally, we wanted to significantly improve the resource economy of our sustainable electrocatalysis in a renewable solvent by the direct use of renewable energy sources, such as photovoltaics or wind power. In general, applying a constant‐current electrolysis, the potential at the working electrode, hence the anode, will adjust until the substrate with the lowest oxidation potential gets consumed. In stark contrast, in the presented cobalta‐electrocatalytic approach, the working potential remains constant until the reaction is completed, owing to its catalytic nature. This offers the possibility to utilize electric current from inexpensive and diverse power sources. With this in mind, we were inspired by the direct use of inexpensive and commercially available photovoltaic cells as the power supply for the depicted constant‐current electrolysis (Scheme 4).^20^ The applied current was thereby fixed and regulated by a custom‐made constant‐current regulator. To our delight, the user‐friendly sunlight‐powered setup delivered the product **3 aa** in a comparable yield of 73 %. The reaction with the solar panel was performed on the 20th of December 2018, the shortest day of the year.

**Scheme 4:**
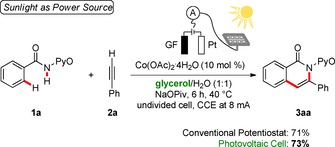
Renewable energy power for electrocatalysis by photovoltaics.

The direct utilization of sunlight for chemical bond transformations has recently attracted considerable attention.^25^ In contrast, the direct exploitation of wind power to drive electrocatalytic transformations is, to the best of our knowledge, thus far unprecedented. Applying a commercially available wind turbine and a digital current regulator in a proof‐of‐concept study, we performed the desired cobalta‐electrocatalyzed C−H/N−H activation in biomass‐derived glycerol to generate isoquinolone **3 aa** in comparable yields (Scheme 5). The slightly diminished reaction outcome can be explained by small current variations. These results clearly show that the envisioned metalla‐electrocatalyzed C−H activation can be put into practice with a simple and renewable power source to drive to the desired transformation.

**Scheme 5:**
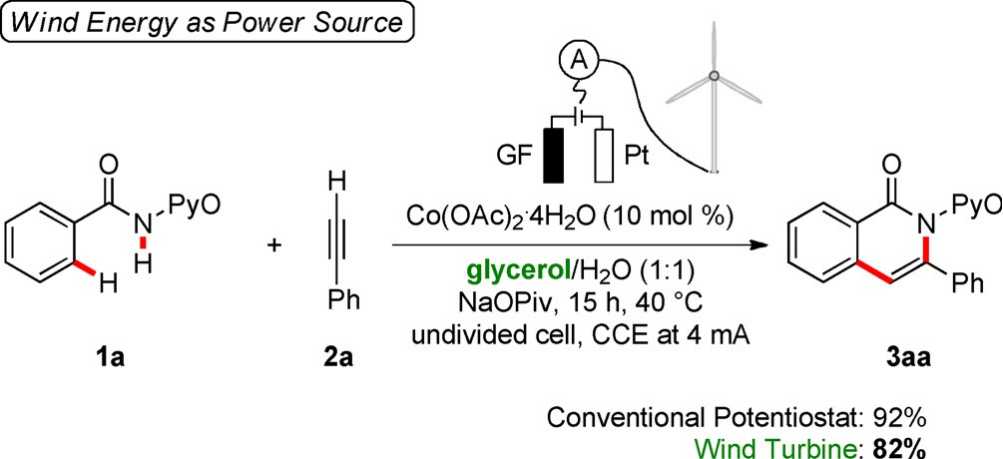
Wind turbine for metalla‐electrocatalyzed C−H activation.

In summary, we have disclosed the unprecedented application of biomass‐derived glycerol as a reaction medium for electro‐enabled C−H activation reactions. The resource economy of our strategy was substantiated by the direct use of renewable energies for chemical C−C/N−C bond formations, with H_2_ as the only side product by facile HER.^6^ Thus, a Cp*‐free cobalt catalyst enabled the sustainable C−H activation of amides in the absence of toxic metal oxidants. The mild C−H/N−H functionalization readily occurred in aqueous glycerol at 40 °C. Importantly, we also demonstrated that renewable solar and wind energy can directly be employed for electrocatalytic C−H activations. The merger of renewable solvents and alternative forms of energy for molecular catalysis should prove invaluable for establishing more sustainable future energy economies.

## Conflict of interest

*The authors declare no conflict of interest*.

## Supporting information

As a service to our authors and readers, this journal provides supporting information supplied by the authors. Such materials are peer reviewed and may be re‐organized for online delivery, but are not copy‐edited or typeset. Technical support issues arising from supporting information (other than missing files) should be addressed to the authors.

Supplementary
