# Supplementary material for: Cobalta‐Electrocatalyzed C−H Activation in Biomass‐Derived Glycerol: Powered by Renewable Wind and Solar Energy
Source: ChemSusChem. 2020 Jan 30;13(4):668–71. doi: 10.1002/cssc.202000057 (PMC7065255; doi:10.1002/cssc.202000057)

CHEMISTRY & SUSTAINABILITY
CHEMSUSCHEM
ENERGY & MATERIALS

Supporting Information

Cobalta-Electrocatalyzed C–H Activation in Biomass-Derived Glycerol: Powered by Renewable Wind and Solar Energy

Tjark H. Meyer, Gleb A. Chesnokov, and Lutz Ackermann*[a]

cssc_202000057_sm_miscellaneous_information.pdf

Table of Contents

General Remarks....2
Optimization of the Reaction Conditions....3
General Procedure **A** for Electrochemical Alkyne Annulations in Aqueous Glycerol....5
General Procedure **B** for Electrochemical C–H Activation with Allenes in Aqueous Glycerol5
Renewable Energy Power Setup....6
Cobaltaelectro-Catalyzed C–H Activation Powered by Sunlight....7
Cobaltaelectro-Catalyzed C–H Activation Powered by Wind....7
Characterization Data....10
Cyclic Voltammetry....25
References....26
^{1}H- and ^{13}C-NMR Spectra....27

General Remarks

Electrocatalytic reactions were carried out in undivided electrochemical cells (10 mL) using pre-dried glassware. Benzamides **1** and $Co(OAc)_3$ were synthesized according to previously described methods[1] and alkynes **2** were used as obtained by commercial sources. Platinum electrodes (10 mm × 15 mm × 0.125 mm, 99.9%, obtained from ChemPur® Karlsruhe, Germany) and graphite felt electrodes (10 mm × 15 mm × 6 mm, SIGRACELL®GFA 6 EA, obtained from SGL Carbon) were connected using stainless steel adapters. Electrocatalysis was conducted using an AXIOMET AX-3003P potentiostat in constant current (CCE) mode; CV studies were performed using a Metrohm Autolab PGSTAT204 workstation and Nova 2.1 software. Yields refer to isolated compounds, estimated to be >95% pure as determined by ^{1}H-NMR spectroscopy. Chromatography: Merck silica gel 60 (40–63 µm). NMR: Spectra were recorded on a Varian Mercury 300, Varian Inova 500 or Bruker Avance III 300, Bruker Avance III HD 400 and Bruker Avance III HD 500 in the solvent indicated; chemical shifts (δ) are given in ppm relative to the residual solvent peak. All IR spectra were recorded on a Bruker FT-IR Alpha device. MS: EI-MS- and ESI-MS-spectra were recorded with Finnigan MAT 95, 70 eV and Finnigan LCQ; High resolution mass spectrometry (HR-MS) with APEX IV 7T FTICR. M. p.: Stuart melting point apparatus SMP3, Barloworld Scientific, values are uncorrected.

Optimization of the Reaction Conditions

Table S-1: Solvent Optimization.[a]

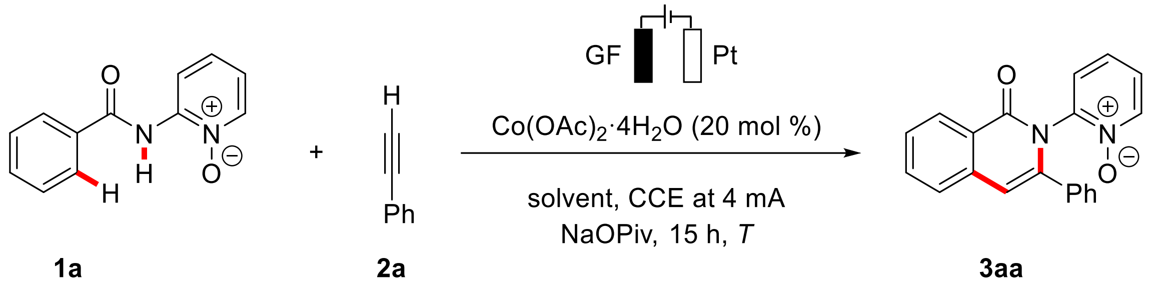

Entry	Solvent	*T* [°C]	Yield [%]
1[b]	DCE	40	43
2[b]	DCM	40	53
3[b]	DMF	40	21
4	DMSO	40	29
5[b]	MeCN	40	51
6	HFIP	40	21
7	TFE	40	87[c]
8[b]	THF	40	18
**9**	**MeOH**	**40**	**82 (78)[d]**
10	H_2O/MeOH (1:1)	23	78 (76)
11	H_2O	23	62
12	Glycerol	40	24
13	Glycerol/H_2O (1:1)	23	47
**14**	**Glycerol/H_2O (1:1)**	**40**	**70 (71)**
15	Glycerol/H_2O (1:1)	60	72
16	2-MeTHF[b]	40	69
17	2-MeTHF/H_2O	40	65
18	Furfuryl alcohol/H_2O (1:1)	40	61
19	GVL/H_2O (1:1)	40	56
20	GVL/Glycerol (1:1)	40	9

[a] Reaction conditions: Undivided cell, **1a** (0.50 mmol), **2a** (1.00 mmol), $Co(OAc)_2{\cdot}4H_2O$ (20 mol %), NaOPiv (2.00 equiv), solvent (5 mL), CCE = 4 mA, 15 h, graphite felt anode, Pt-plate cathode. Conversion of **3aa** determined by ^{1}H-NMR analysis with 1,3,5-trimethoxybenzene as an internal standard are shown. Isolated yield in parentheses. [b] Addition of $LiClO_4$ (1.0 equiv). [c] Oxygenated product (5%) was formed as a byproduct, based on ^{1}H NMR conversion. [d] Oxygenated product (11 mg, 45 µmol, 9%) was formed as a byproduct.

Table S-2: Catalyst Optimization and Additive Effects.[a]

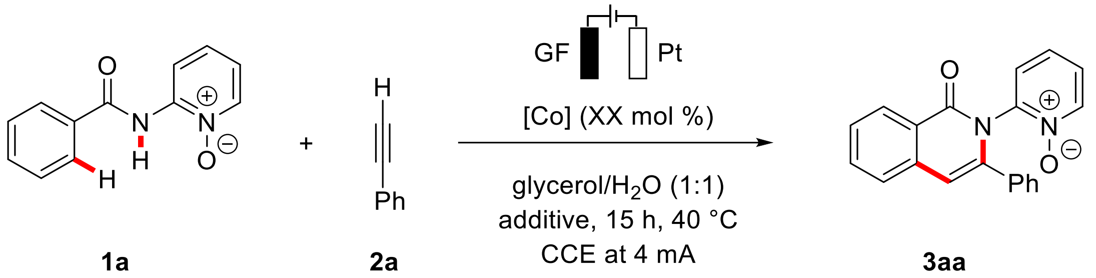

Entry	Additive (equiv)	[Co]	XX [mol %]	Yield [%]
1	NaOPiv (2.0)	$Co(OAc)_2{\cdot}4H_2O$	20	70 (71)
2	NaOPiv (1.0)	$Co(OAc)_2{\cdot}4H_2O$	20	99 (94)
3	NaOPiv (0.5)	$Co(OAc)_2{\cdot}4H_2O$	20	95
**4**	**NaOPiv (2.0)**	**$Co(OAc)_2{\cdot}4H_2O$**	**10**	**96 (92)**
5	NaOPiv (1.0)	$Co(OAc)_2{\cdot}4H_2O$	10	93
**6**	**NaOPiv (2.0)**	**$Co(OAc)_2{\cdot}4H_2O$**	**5**	**90**
7	NaOPiv (1.0)	$Co(OAc)_2{\cdot}4H_2O$	5	73
8	NaOPiv (0.5)	$Co(OAc)_2{\cdot}4H_2O$	5	67
9[b]	NaOPiv (2.0)	$Co(OAc)_2{\cdot}4H_2O$	10	54
10[c]	NaOPiv (2.0)	$Co(OAc)_2{\cdot}4H_2O$	10	81
11	Na_2CO_3 (2.0)	$Co(OAc)_2{\cdot}4H_2O$	10	29
12	HOPiv (2.0)	$Co(OAc)_2{\cdot}4H_2O$	10	14
13	NaOAc (2.0)	$Co(OAc)_2{\cdot}4H_2O$	10	31
14	KOAc (2.0)	$Co(OAc)_2{\cdot}4H_2O$	10	33
15	NaOH (2.0)	$Co(OAc)_2{\cdot}4H_2O$	10	---
16	NaOPiv (2.0)	$Co(OAc)_3$	10	92 (91)
17	NaOPiv (2.0)	$Co(OAc)_3$	5	82
18[d]	NaOPiv (2.0)	$Co(OAc)_2{\cdot}4H_2O$	10	traces
19[e]	NaOPiv (2.0)	$Co(OAc)_2{\cdot}4H_2O$	10	(71)
20	NaOPiv (2.0)	---	---	---
21	---	$Co(OAc)_2{\cdot}4H_2O$	20	19

[a] Reaction conditions: Undivided cell, **1a** (0.50 mmol), **2a** (1.00 mmol), [Co] (XX mol %), additive (XX equiv), glycerol/H_2O (1:1, 5 mL), 40 °C, 4 mA, 15 h, graphite felt anode, Pt-plate cathode. Conversion of **3aa** determined by ^{1}H-NMR analysis with 1,3,5-trimethoxybenzene as an internal standard are shown. Isolated yield in parentheses. [b] **2a** (0.6 mmol). [c] **2a** (0.75 mmol). [d] no current. [e] 8 mA, 6 h.

General Procedure A for Electrochemical Alkyne Annulations in Aqueous Glycerol

The electrocatalysis was carried out in an undivided cell, with a graphite felt (GF) anode (25 mm × 10 mm × 6.0 mm) and a platinum cathode (25 mm × 10 mm × 0.125 mm). Benzamide **1** (0.50 mmol, 1.00 equiv), alkyne **2** (1.00 mmol, 2.00 equiv), NaOPiv (124 mg, 1.00 mmol, 2.00 equiv), glycerol/H_2O (5 mL, 1:1) and $Co(OAc)_2{\cdot}4H_2O$ (12.7 mg, 10 mol %) were placed in a 10 mL cell. Electrocatalysis was performed at 40 °C with a constant current of 4 mA maintained for 15 h. Then, the DC-power supply was stopped, and the reaction mixture was diluted with CH_2Cl_2 (2.0 mL). The graphite felt anode was washed with CH_2Cl_2 (3 × 5.0 mL) in an ultrasonic cleaner (3 x 3 min). The combined washings were added to the reaction mixture and the combined phases were washed with H_2O (15 mL). The aqueous phase was extracted with CH_2Cl_2 (4 × 10 mL). The crude extracts were then dried over Na_2SO_4. Evaporation of the solvent and subsequent column chromatography on silica gel afforded the corresponding products **3**.

General Procedure B for Electrochemical C–H Activation with Allenes in Aqueous Glycerol

The electrocatalysis was carried out in an undivided cell, with a graphite felt (GF) anode (25 mm × 10 mm × 6.0 mm) and a platinum cathode (25 mm × 10 mm × 0.125 mm). Benzamide **1** (0.50 mmol, 1.00 equiv), allene **4** (1.00 mmol, 2.00 equiv), NaOPiv (124 mg, 1.00 mmol, 2.00 equiv), glycerol/H_2O (5 mL, 1:1) and $Co(OAc)_2{\cdot}4H_2O$ (12.7 mg, 10 mol %) were placed in a 10 mL cell. Electrocatalysis was performed at 40 °C with a constant current of 2 mA maintained for 15 h. Then, the DC-power supply was stopped, and the reaction mixture was diluted with CH_2Cl_2 (2.0 mL). The graphite felt anode was washed with CH_2Cl_2 (3 × 5.0 mL) in an ultrasonic cleaner (3 x 3 min). The combined washings were added to the reaction mixture and the combined phases were washed with H_2O (15 mL). The aqueous phase was extracted with CH_2Cl_2 (4 × 10 mL). The crude extracts were then dried over Na_2SO_4. Evaporation of the solvent and subsequent column chromatography on silica gel afforded the corresponding product **5**.

Renewable Energy Power Setup

For the electrocatalysis powered by sunlight, a commercially available photovoltaic cell (Conrad Electronic SE, TPS-103 6 W, 17.5 V max. voltage, 428 mA max. current, 467 mm x 161 mm x 19 mm) was used (Figure S-1).

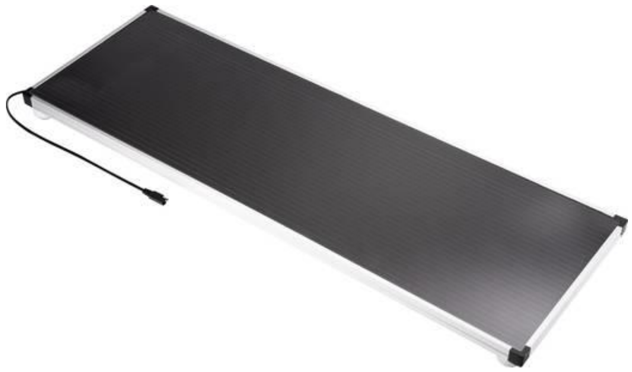

Figure S-1. Amorphous silicon solar panel used for the reactions.

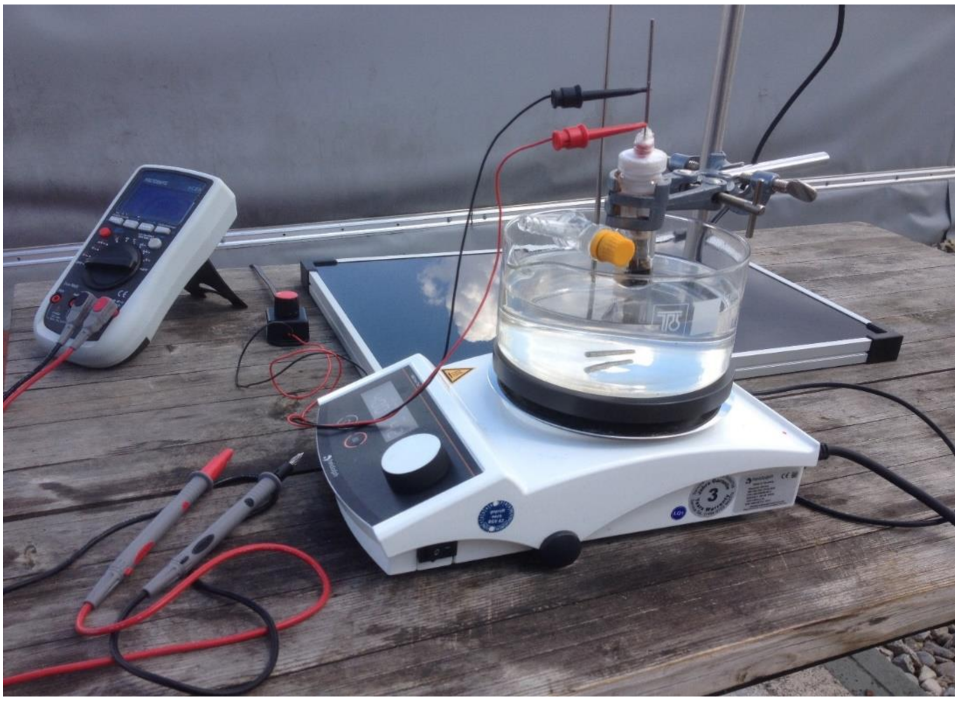

Figure S-2. Cobaltaelectrocatalysis powered by a photovoltaic cell.

The output current was controlled with a customized and normalized constant current regulator and regularly double checked with an ammeter. The electrocatalysis was performed by sunlight and the reaction temperature was controlled with an oil bath reservoir.

Cobaltaelectro-Catalyzed C–H Activation Powered by Sunlight

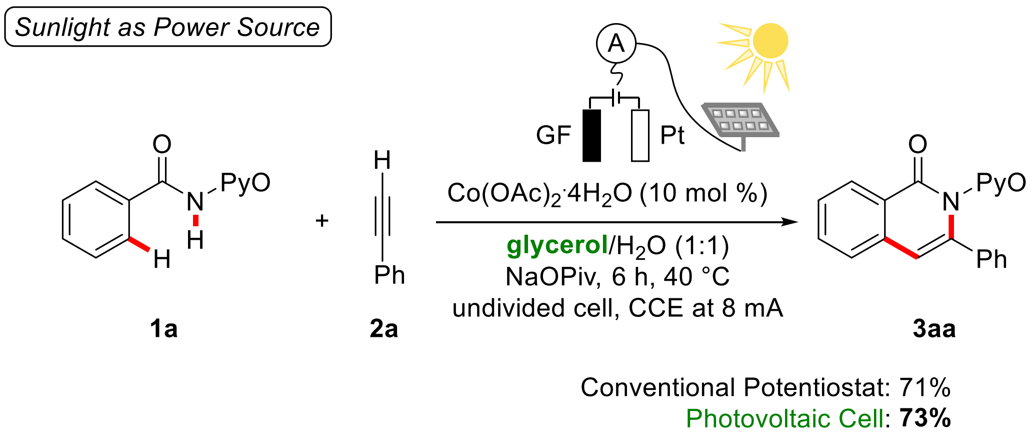

The electrocatalysis was carried out in an undivided cell, with a graphite felt (GF) anode (25 mm × 10 mm × 6.0 mm) and a platinum cathode (25 mm × 10 mm × 0.125 mm). Benzamide **1a** (0.50 mmol, 1.00 equiv), alkyne **2a** (1.00 mmol, 2.00 equiv), NaOPiv (124 mg, 1.00 mmol, 2.00 equiv), glycerol/H_2O (5.0 mL, 1:1) and $Co(OAc)_2{\cdot}4H_2O$ (12.7 mg, 10 mol %) were placed in a 10 mL cell. Electrocatalysis was performed at 40 °C with a constant current of 8 mA maintained for 6 h (Figure S-2). The solar panel was disconnected, and the reaction mixture was diluted with CH_2Cl_2 (2.0 mL). The graphite felt anode was washed with CH_2Cl_2 (3 × 5.0 mL) in an ultrasonic cleaner (3 x 3 min). The combined washings were added to the reaction mixture and the combined phases were washed with H_2O (15 mL). The aqueous phase was extracted with CH_2Cl_2 (4 × 10 mL). The crude extracts were then dried over Na_2SO_4. Evaporation of the solvent and subsequent column chromatography on silica gel afforded the corresponding product **3aa** (114 mg, 73%) as a white solid. The yield is in good agreement with the otherwise commonly employed DC-power source (Table S-2, entry 19).

Cobaltaelectro-Catalyzed C–H Activation Powered by Wind

For the electrocatalysis performed by wind, a commercially available wind turbine (Texenergy Limited, Infinite Air, 7.5–10 W, 1.5–2 A at 5 V output voltage, blade diameter: 320 mm) was used (Figure S-3a). The output current was controlled with a customized and normalized constant current regulator and regularly double checked with an ammeter. The reaction temperature was controlled with an oil bath reservoir. The wind turbine was powered with an external fan, to ensure a steady wind velocity (Figure S-3b).

A)

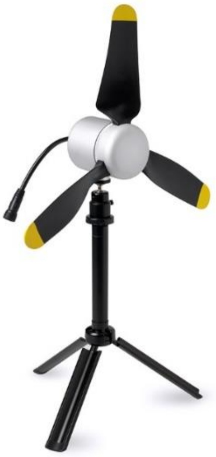

B)

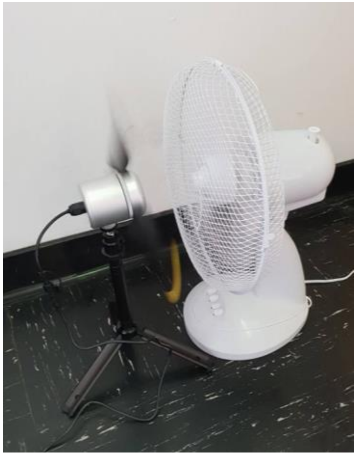

Figure S-3. A) Texenergy Limited, Infinite Air windturbine. B) Windturbine powered by a fan.

Attempts to perform the reaction with beyond the laboratory conditions felt short in giving reproducible reaction outcome.

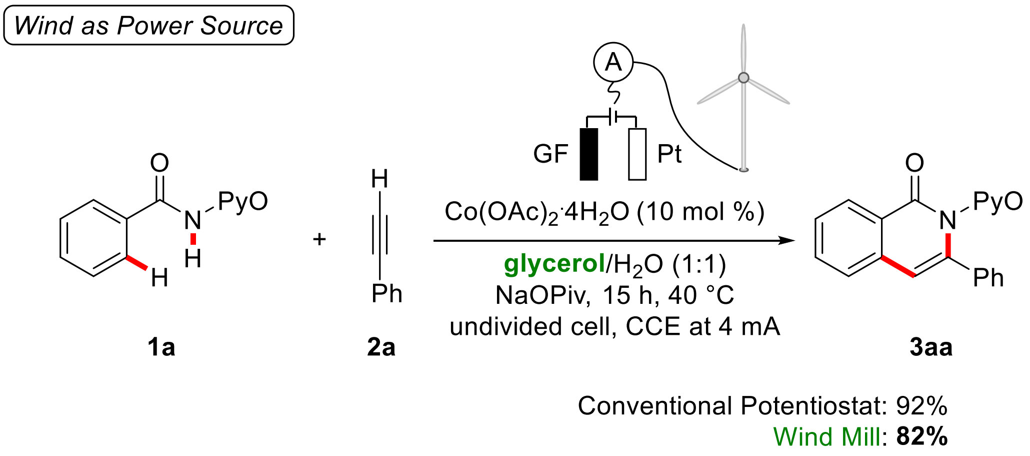

The electrocatalysis was carried out in an undivided cell, with a graphite felt (GF) anode (25 mm × 10 mm × 6.0 mm) and a platinum cathode (25 mm × 10 mm × 0.125 mm). Benzamide **1a** (0.50 mmol, 1.00 equiv), alkyne **2a** (1.00 mmol, 2.00 equiv), NaOPiv (124 mg, 1.00 mmol, 2.00 equiv), glycerol/H_2O (5.0 mL, 1:1) and $Co(OAc)_2·4H_2O$ (12.7 mg, 10 mol %) were placed in a 10 mL cell. Electrocatalysis was performed at 40 °C with a constant current of 4 mA maintained for 15 h. The wind turbine was stopped, and the reaction mixture was diluted with CH_2Cl_2 (2.0 mL). The graphite felt anode was washed with CH_2Cl_2 (3 × 5.0 mL) in an ultrasonic cleaner (3 x 3 min). The combined washings were added to the reaction mixture and the combined phases were washed with H_2O (15 mL). The aqueous phase was extracted

with CH_2Cl_2 (4 × 10 mL). The crude extracts were then dried over Na_2SO_4. Evaporation of the solvent and subsequent column chromatography on silica gel afforded the corresponding product **3aa** (128 mg, 82%) as a white solid. The slightly diminished yield can be reasoned by changes in the current, due to inconsistent power supply by the turbine.

Characterization Data

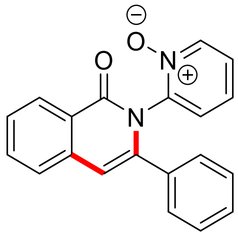

2-[1-Oxo-3-phenylisoquinolin-2(1*H*)-yl]-pyridine-1-oxide (3aa)

The general procedure **A** was followed using benzamide **1a** (107 mg, 0.50 mmol) and alkyne **2a** (102 mg, 1.00 mmol). Purification by column chromatography on silica gel (CH_2Cl_2/acetone 3:1) yielded **3aa** (145 mg, 92%) as a white solid. M. p.: 223–225 °C. ^{1}H-NMR (300 MHz, $CDCl_3$): δ = 8.46–8.37 (m, 1H), 8.23–8.14 (m, 1H), 7.70 (ddd, *J* = 8.0, 7.1, 1.4 Hz, 1H), 7.53 (dd, *J* = 7.2, 0.7 Hz, 1H), 7.50 (ddd, *J* = 8.2, 7.1, 1.3 Hz, 1H), 7.44–7.35 (m, 2H), 7.28–7.16 (m, 3H), 7.16–7.08 (m, 2H), 7.06 (ddd, *J* = 8.5, 6.9, 1.1 Hz, 1H), 6.61 (s, 1H). ^{13}C-NMR (126 MHz, $CDCl_3$): δ = 162.0 (C_q), 145.3 (C_q), 142.7 (C_q), 139.8 (CH), 136.9 (C_q), 134.7 (C_q), 133.2 (CH), 128.9 (CH), 128.2 (CH), 128.0 (CH), 127.8 (CH), 127.5 (CH), 126.9 (CH), 126.2 (CH), 125.3 (CH), 124.9 (CH), 124.8 (C_q), 107.9 (CH). IR (ATR): 3074, 1658, 1482, 1425, 1382, 1257, 1143, 890, 518 cm^{-1}. MS (EI) *m/z* (relative intensity): 314 (20) $[M]^+$, 298 (50), 269 (60), 194 (30), 181 (100), 165 (30), 78.0 (93). HR-MS (EI) *m/z* calc. for $C_{20}H_{14}N_2O_2$ $[M]^+$: 314.1055, found: 314.1052. The analytical data correspond with those reported in the literature.[2]

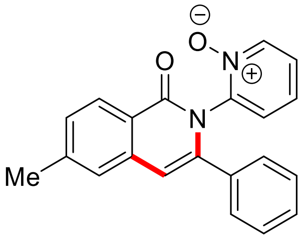

2-[6-Methyl-1-oxo-3-phenylisoquinolin-2(1*H*)-yl]-pyridine-1-oxide (3ba)

A modified procedure **A** was followed using benzamide **1b** (114 mg, 0.50 mmol), alkyne **2a** (102 mg, 1.00 mmol), $Co(OAc)_3$ (11.3 mg, 10 mol %). The reaction cell was mounted in a closed ultrasonic cleaner. The sonication and electrolysis were started and stopped simultaneously. Purification by column chromatography on silica gel (CH_2Cl_2/acetone 3:1) yielded **3ba** (120 mg, 73%) as a white solid. M. p. = 222–223 °C. ^{1}H-NMR (400 MHz, $CDCl_3$):

δ = 8.31 (d, J = 8.1 Hz, 1H), 8.24–8.18 (m, 1H), 7.43–7.36 (m, 2H), 7.35–7.29 (m, 2H), 7.26–7.18 (m, 3H), 7.16–7.10 (m, 2H), 7.09–7.02 (m, 1H), 6.53 (s, 1H), 2.48 (s, 3H). ^{13}C-NMR (101 MHz, $CDCl_3$): δ = 162.0 (C_q), 145.8 (C_q), 144.2 (C_q), 142.8 (C_q), 140.2 (CH), 137.3 (C_q), 135.1 (C_q), 129.1 (CH), 128.8 (CH), 128.5 (CH), 128.4 (CH), 128.1 (CH), 127.9 (CH), 126.3 (CH), 125.5 (CH), 125.2 (CH), 122.8 (C_q), 108.1 (CH), 22.0 (CH_3). IR (ATR): 3064, 1665, 1620, 1479, 1261, 902, 763, 725 cm^{-1}. MS (EI) *m/z* (relative intensity): 328 (20) $[M]^+$, 312.1 (15), 283 (15), 208 (25), 181 (100), 165 (15), 78 (65). HR-MS (EI) *m/z* calc. for $C_{21}H_{16}N_2O_2$ $[M]^+$: 328.1212, found: 328.1206. The analytical data correspond with those reported in the literature.[2]

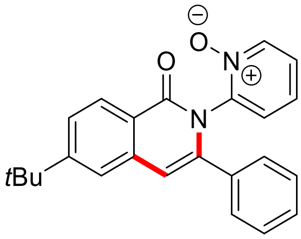

2-[6-(*tert*-Butyl)-1-oxo-3-phenylisoquinolin-2(1*H*)-yl]-pyridine-1-oxide (3ca)

The general procedure **A** was followed using benzamide **1c** (135 mg, 0.50 mmol) and alkyne **2a** (102 mg, 1.00 mmol). Purification by column chromatography on silica gel (CH_2Cl_2/acetone 3:1) yielded **3ca** (128 mg, 69%) as a white solid. M. p.: 248–250 °C. ^{1}H-NMR (300 MHz, $CDCl_3$): δ = 8.39–8.33 (m, 1H), 8.24–8.19 (m, 1H), 7.57 (dd, J = 8.5, 1.9 Hz, 1H), 7.54–7.52 (m, 1H), 7.43–7.37 (m, 2H), 7.26–7.18 (m, 3H), 7.17–7.10 (m, 2H), 7.09–7.03 (m, 1H), 6.60 (s, 1H), 1.40 (s, 9H). ^{13}C-NMR (126 MHz, $CDCl_3$): δ = 161.9 (C_q), 157.1 (C_q), 145.7 (C_q), 142.6 (C_q), 140.1 (CH), 137.1 (C_q), 135.1 (C_q), 129.1 (CH), 128.3 (CH), 128.3 (CH), 128.1 (CH), 127.8 (CH), 125.4 (CH), 125.3 (CH), 125.0 (CH), 122.8 (C_q), 122.6 (CH), 108.6 (CH), 35.5 (C_q), 31.3 (CH_3). IR (ATR): 1681, 1617, 1606, 1488, 1270, 939, 764, 698 cm^{-1}. MS (EI) *m/z* (relative intensity): 370 (15) $[M]^+$, 325 (10), 250 (12), 235 (15), 181 (100), 78 (40). HR-MS (EI) *m/z* calc. for $C_{24}H_{22}N_2O_2$ $[M]^+$: 370.1681, found: 370.1683. The analytical data correspond with those reported in the literature.[2]

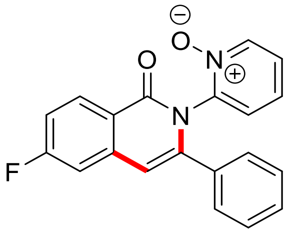

2-[6-Fluoro-1-oxo-3-phenylisoquinolin-2(1*H*)-yl]-pyridine-1-oxide (3da)

The general procedure **A** was followed using benzamide **1d** (116 mg, 0.50 mmol) and alkyne **2a** (102 mg, 1.00 mmol). Purification by column chromatography on silica gel (CH_2Cl_2/acetone 6:1) yielded **3da** (109 mg, 66%) as a white solid. M. p.: 191–192 °C. ^{1}H-NMR (300 MHz, $CDCl_3$): δ = 8.44 (dd, J = 9.6, 5.7 Hz, 1H), 8.21 (dd, J = 6.4, 1.6 Hz, 1H), 7.40 (dd, J = 7.8, 1.8 Hz, 2H), 7.31–7.26 (m, 1H), 7.25–7.14 (m, 5H), 7.14–7.11 (m, 1H), 7.10–7.03 (m, 1H), 6.54 (s, 1H). ^{13}C-NMR (125 MHz, $CDCl_3$): δ = 166.1 (d, $^1J_{C–F}$ = 254.1 Hz, C_q), 161.4 (C_q), 145.4 (C_q), 144.3 (C_q), 140.2 (CH), 139.6 (d, $^3J_{C–F}$ = 10.6 Hz, C_q), 134.7 (C_q), 131.9 (d, $^3J_{C–F}$ = 10.2 Hz, CH), 129.5 (CH), 128.5 (CH), 128.0 (CH), 127.8 (CH), 125.7 (CH), 125.3 (CH), 121.7 (d, $^4J_{C–F}$ = 1.8 Hz, C_q), 115.8 (d, $^2J_{C–F}$ = 23.4 Hz, CH), 111.5 (d, $^2J_{C–F}$ = 22.1 Hz, CH), 107.5 (d, $^4J_{C–F}$ = 3.1 Hz, CH). ^{19}F-NMR (376 MHz, $CDCl_3$): δ = –104.7 (td, J = 8.9, 5.7 Hz). IR (ATR): 1666, 1610, 1506, 1479, 1421, 1270, 866, 758 cm^{-1}. MS (EI) *m/z* (relative intensity): 332 (55) [M]$^+$, 315 (10), 287 (15), 212 (25), 181 (100), 78 (65). HR-MS (EI) *m/z* calc. for $C_{20}H_{13}FN_2O_2$ [M+H]$^+$: 332.0961, found: 332.0966. The analytical data correspond with those reported in the literature.[2]

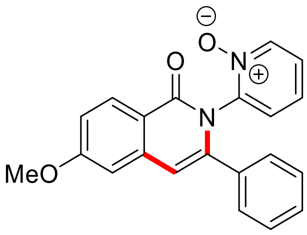

2-[6-Methoxy-1-oxo-3-phenylisoquinolin-2(1*H*)-yl]-pyridine-1-oxide (3ea)

The general procedure **A** was followed using benzamide **1e** (122 mg, 0.50 mmol) and alkyne **2a** (102 mg, 1.00 mmol). Purification by column chromatography on silica gel (CH_2Cl_2/acetone 3:1) yielded **3ea** (119 mg, 69%) as a white solid. M. p.: 201–202 °C. ^{1}H-NMR (400 MHz, $CDCl_3$): δ = 8.38–8.29 (m, 1H), 8.26–8.11 (m, 1H), 7.44–7.34 (m, 2H), 7.26–7.17 (m, 3H), 7.17–7.09 (m, 2H), 7.08–7.02 (m, 2H), 6.91 (d, J = 2.4 Hz, 1H), 6.52 (s, 1H), 3.92 (s, 3H). ^{13}C-NMR (125 MHz, $CDCl_3$): δ = 163.8 (C_q), 161.6 (C_q), 145.8 (C_q), 143.4 (C_q), 140.2 (CH), 139.4 (C_q), 135.1 (C_q), 130.7 (CH), 129.2 (CH), 128.3 (CH), 128.0 (CH), 127.9 (CH), 125.5 (CH), 125.1 (CH), 118.8 (C_q), 116.4 (CH), 108.0 (CH), 107.7 (CH), 55.7 (CH_3). IR (ATR): 1656, 1604, 1486, 1378, 1247, 1145, 767, 692 cm^{-1}. MS (EI) *m/z* (relative intensity): 344 (20) [M$^+$], 299 (10), 255 (15), 224 (20), 181 (100), 135 (10), 78 (45). HR-MS (EI) *m/z* calc. for

$C_{21}H_{16}N_2O_3$ [M$^+$] 344.1161, found 344.1149. The analytical data correspond with those reported in the literature.[3]

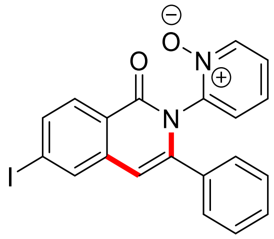

2-[6-Iodo-1-oxo-3-phenylisoquinolin-2(1*H*)-yl]-pyridine-1-oxide (3fa)

The general procedure **A** was followed using benzamide **1f** (170 mg, 0.50 mmol) and alkyne **2a** (102 mg, 1.00 mmol). Purification by column chromatography on silica gel (CH_2Cl_2/acetone 6:1) yielded **3fa** (91.4 mg, 41%) as a white solid. M. p.: 251–252 °C. ^{1}H-NMR (400 MHz, $CDCl_3$): δ = 8.22 (ddd, *J* = 6.5, 1.5, 0.7 Hz, 1H), 8.11 (dd, *J* = 8.5, 0.6 Hz, 1H), 7.96 (d, *J* = 1.6 Hz, 1H), 7.80 (dd, *J* = 8.5, 1.7 Hz, 1H), 7.41–7.35 (m, 2H), 7.30–7.26 (m, 1H), 7.26–7.21 (m, 2H), 7.18–7.11 (m, 2H), 7.10–7.05 (m, 1H), 6.49 (s, 1H). ^{13}C-NMR (101 MHz, $CDCl_3$): δ = 161.9 (C_q), 145.4 (C_q), 144.1 (C_q), 140.2 (CH), 138.6 (C_q), 136.3 (CH), 135.3 (CH), 134.7 (C_q), 130.1 (CH), 129.5 (CH), 128.5 (CH), 128.0 (CH), 127.8 (CH), 125.7 (CH), 125.2 (CH), 124.3 (C_q), 106.8 (CH), 101.6 (C_q). IR (ATR): 1666, 1618, 1597, 1578, 1271, 898, 761, 726 cm^{-1}. MS (EI) *m/z* (relative intensity): 440 (20) [M]$^+$, 395 (15), 320 (20), 268 (10), 181 (100), 78 (50). HR-MS (EI) *m/z* calc. for $C_{20}H_{13}IN_2O_2$ [M]$^+$: 440.0022, found: 440.0024. The analytical data correspond with those reported in the literature.[2]

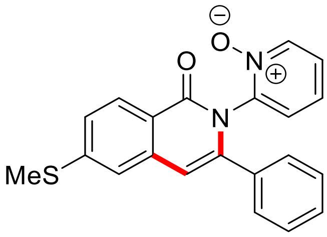

2-[6-(Methylthio)-1-oxo-3-phenylisoquinolin-2(1*H*)-yl]-pyridine-1-oxide (3ga)

The general procedure **A** was followed using benzamide **1g** (130 mg, 0.50 mmol) and alkyne **2a** (102 mg, 1.00 mmol). Purification by column chromatography on silica gel (CH_2Cl_2/acetone 3:1) yielded **3ga** (110 mg, 61%) as a white solid. M. p.: 218–220 °C. ^{1}H-NMR (500 MHz, $CDCl_3$): δ = 8.28 (dd, *J* = 8.4, 1.8 Hz, 1H), 8.25–8.14 (m, 1H), 7.42–7.36 (m, 2H), 7.32 (dd, *J* = 8.4, 1.8 Hz, 1H), 7.28 (d, *J* = 1.9 Hz, 1H), 7.26–7.19 (m, 3H), 7.18–7.11 (m, 2H), 7.11–7.02

(m, 1H), 6.51 (s, 1H), 2.57 (s, 3H). ^{13}C-NMR (126 MHz, $CDCl_3$): δ = 161.8 (C_q), 146.4 (C_q), 145.6 (C_q), 143.6 (C_q), 140.2 (CH), 137.6 (C_q), 134.9 (C_q), 129.3 (CH), 128.7 (CH), 128.4 (CH), 128.0 (CH), 127.9 (CH), 125.6 (CH), 125.4 (CH), 124.8 (CH), 121.7 (C_q), 121.2 (CH), 107.6 (CH), 15.0 (CH_3). IR (ATR): 1663, 1587, 1431, 1372, 1269, 916, 730, 703 cm^{-1}. MS (EI) *m/z* (relative intensity): 360 (20) [M]$^+$, 315 (10), 240 (20), 193 (10), 181 (100), 78 (45). HR-MS (EI) *m/z* calc. for $C_{21}H_{16}N_2O_2S$ [M]$^+$: 360.0932, found: 360.0925.

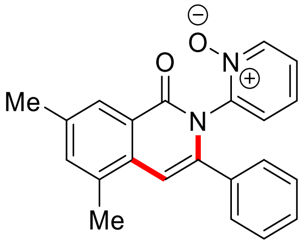

2-[5,7-Dimethyl-1-oxo-3-phenylisoquinolin-2(1*H*)-yl]-pyridine-1-oxide (3ha)

A modified general procedure **A** was followed using benzamide **1h** (121 mg, 0.50 mmol), alkyne **2a** (102 mg, 1.00 mmol) and $Co(OAc)_3$ (11.3 mg, 10 mol %). Purification by column chromatography on silica gel (CH_2Cl_2/acetone 3:1) yielded **3ha** (108 mg, 63%) as a white solid. M. p.: 238–239 °C. ^{1}H-NMR (400 MHz, $CDCl_3$): δ = 8.20 (dd, *J* = 6.9, 1.5 Hz, 1H), 8.11 (s, 1H), 7.45–7.39 (m, 2H), 7.37 (s, 1H), 7.26–7.20 (m, 3H), 7.16–7.11 (m, 2H), 7.06 (ddd, *J* = 8.2, 7.1, 1.4 Hz, 1H), 6.68 (d, *J* = 0.8 Hz, 1H), 2.51 (s, 3H), 2.45 (s, 3H). ^{13}C-NMR (125 MHz, $CDCl_3$): δ = 162.3 (C_q), 145.9 (C_q), 141.3 (C_q), 140.2 (CH), 137.0 (C_q), 135.9 (CH), 135.5 (C_q), 133.7 (C_q), 133.6 (C_q), 129.1 (CH), 128.3 (CH), 128.2 (CH), 127.8 (CH), 126.1 (CH), 125.5 (CH), 125.3 (C_q), 125.0 (CH), 105.1 (CH), 21.6 (CH_3), 19.0 (CH_3). IR (ATR): 1709, 1668, 1489, 1251, 851, 756, 700, 578 cm^{-1}. MS (EI) *m/z* (relative intensity): 342 (10) [M]$^+$, 326 (10), 297 (10), 222 (25), 207 (15), 181 (100), 78 (60). HR-MS (ESI) *m/z* calc. for $C_{22}H_{19}N_2O_2$ [M+H]$^+$: 343.1441, found: 343.1444. The analytical data correspond with those reported in the literature.[2]

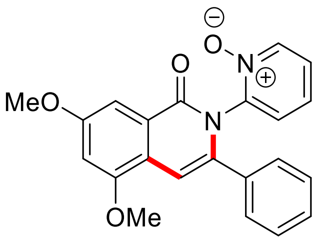

2-[5,7-Dimethoxy-1-oxo-3-phenylisoquinolin-2(1*H*)-yl]-pyridine-1-oxide (3ia)

The general procedure **A** was followed using benzamide **1i** (137 mg, 0.50 mmol) and alkyne **2a** (102 mg, 1.00 mmol). Purification by column chromatography on silica gel (CH_2Cl_2/acetone 3:1) yielded **3ia** (146 mg, 78%) as a white solid. M. p.: 254–255 °C. ^{1}H-NMR (400 MHz, $CDCl_3$): δ = 8.23 (dd, *J* = 6.6, 1.3 Hz, 1H), 7.44 (dd, *J* = 2.4, 0.6 Hz, 1H), 7.41–7.37 (m, 2H), 7.24–7.17 (m, 3H), 7.17–7.11 (m, 1H), 7.11–7.02 (m, 2H), 6.94 (d, *J* = 0.7 Hz, 1H), 6.74 (d, *J* = 2.4 Hz, 1H), 3.91 (s, 3H), 3.90 (s, 3H). ^{13}C-NMR (101 MHz, $CDCl_3$): δ = 161.6 (C_q), 159.8 (C_q), 156.2 (C_q), 146.0 (C_q), 140.2 (CH), 139.7 (C_q), 135.5 (C_q), 128.9 (CH), 128.3 (CH), 128.2 (CH), 127.6 (CH), 126.7 (C_q), 125.5 (CH), 125.1 (CH), 122.9 (C_q), 104.0 (CH), 102.7 (CH), 99.8 (CH), 56.0 (CH_3), 55.9 (CH_3). IR (ATR): 1665, 1606, 1489, 1429, 1351, 1041, 788, 750 cm^{-1}. MS (EI) *m/z* (relative intensity): 374 (10) $[M]^+$, 358 (10), 269 (10), 254 (10), 181 (100), 78 (45). HR-MS (EI) *m/z* calc. for $C_{22}H_{18}N_2O_4$ $[M]^+$: 374.1267, found: 374.1259. The analytical data correspond with those reported in the literature.[2]

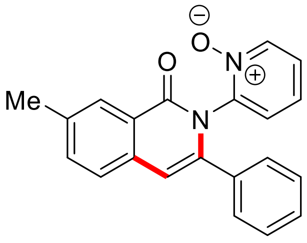

3ja

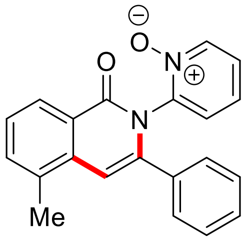

3ja'

2-[7-Methyl-1-oxo-3-phenylisoquinolin-2(1*H*)-yl]-pyridine-1-oxide (3ja) and 2-[5-Methyl-1-oxo-3-phenylisoquinolin-2(1*H*)-yl]-pyridine-1-oxide (3ja')

The general procedure **A** was followed using benzamide **1j** (114 mg, 0.50 mmol) and alkyne **2a** (102 mg, 1.00 mmol). Purification by column chromatography on silica gel (CH_2Cl_2/acetone 3:1) yielded **3ja/3ja'** (130 mg, 83%) as a white solid. M. p.: 218–220 °C. The ratio of **3ja/3ja'** (4:1) was determined by ^{1}H-NMR spectroscopy. Resonances are reported for **3ja**. ^{1}H-NMR (400 MHz, $CDCl_3$): δ = 8.24–8.29 (m, 2H), 7.55–7.50 (m, 1H), 7.47–7.44 (m, 1H), 7.41–7.37 (m, 2H), 7.25–7.20 (m, 3H), 7.14–7.10 (m, 2H), 7.09–7.03 (m, 1H), 6.57 (s, 1H), 2.49 (s, 3H). ^{13}C-NMR (101 MHz, $CDCl_3$): δ = 162.0 (C_q), 145.8 (C_q), 141.8 (C_q), 140.1 (CH), 137.4 (C_q), 135.1 (C_q), 134.8 (CH), 134.8 (C_q), 129.1 (CH), 128.3 (CH), 128.1 (CH), 128.1 (CH), 127.8 (CH), 126.4 (CH), 125.5 (CH), 125.1 (CH), 125.0 (C_q), 108.1 (CH), 21.6 (CH_3). IR (ATR): 1664, 1492, 1430, 1385, 1277, 847, 758, 704 cm^{-1}. MS (EI) *m/z* (relative intensity): 328 (20) $[M]^+$, 283 (10), 208 (35), 193 (10), 181 (100), 78 (65). HR-MS (EI) *m/z* calc. for $C_{21}H_{16}N_2O_2$ $[M]^+$: 328.1212, found: 328.1221.

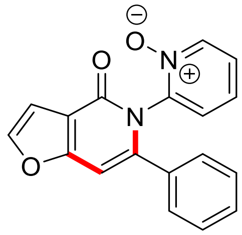

3ka

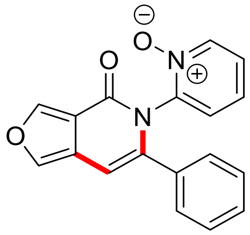

3ka'

2-[4-Oxo-6-phenylfuro[3,2-c]pyridin-5(4*H*)-yl]-pyridine-1-oxide (3ka) and 2-[4-Oxo-6-phenylfuro[3,4-c]pyridin-5(4*H*)-yl]-pyridine 1-oxide (3ka')

The general procedure **A** was followed using 2-(furan-3-carboxamido)pyridine 1-oxide **1k** (102 mg, 0.50 mmol) and alkyne **2a** (102 mg, 1.00 mmol). Purification by column chromatography on silica gel (CH_2Cl_2/acetone 5:1) yielded **3ka**/**3ka'** (79.0 mg, 52%) as a white solid. The ratio of **3ka**/**3ka'** (10:1) was determined by ^{1}H-NMR spectroscopy. Resonances are reported for **3ka**. M. p. = 199–200 °C. ^{1}H-NMR (600 MHz, $CDCl_3$): δ = 8.23–8.13 (m, 1H), 7.55–7.52 (m, 1H), 7.39–7.36 (m, 2H), 7.26–7.25 (m, 1H), 7.23–7.20 (m, 2H), 7.16–7.11 (m, 2H), 7.09–7.04 (m, 1H), 7.02–7.00 (m, 1H), 6.78 (s, 1H). ^{13}C-NMR (126 MHz, $CDCl_3$): δ = 159.8 (C_q), 158.4 (C_q), 145.6 (C_q), 145.1 (C_q), 143.6 (CH), 139.9 (CH), 134.4 (C_q), 129.3 (CH), 128.2 (CH), 127.1 (CH), 127.8 (CH), 125.5 (CH), 125.0 (CH), 114.9 (C_q), 107.8 (CH), 98.2 (CH). IR (ATR): 3112, 1675, 1574, 1488, 1266, 730, 590, 517 cm^{-1}. MS (EI) *m/z* (relative intensity): 304 (35) $[M]^+$, 288 (70), 259 (45), 231 (35), 181 (100), 78 (60). HR-MS (ESI) *m/z* calc. for $C_{18}H_{13}N_2O_3$ $[M+H]^+$: 305.0921, found: 305.0922.

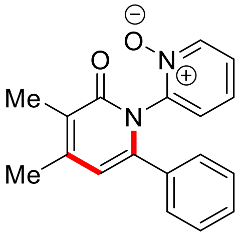

3,4-Dimethyl-2-oxo-6-phenyl-2*H*-[1,2'-bipyridine]-1'-oxide (3la)

The general procedure **A** was followed using alkene **1l** (96.0 mg, 0.50 mmol) and alkyne **2a** (102 mg, 1.00 mmol). Purification by column chromatography on silica gel (CH_2Cl_2/acetone 1:1) yielded **3la** (92.1 mg, 63%) as a white solid. M. p.: 203–204 °C. ^{1}H-NMR (300 MHz, $CDCl_3$): δ = 8.21 (ddd, *J* = 6.4, 1.0, 0.4 Hz, 1H), 7.36–7.30 (m, 2H), 7.25–7.21 (m, 2H), 7.20–7.16 (m, 1H), 7.15–7.10 (m, 1H), 7.08–7.03 (m, 2H), 6.15 (s, 1H), 2.25 (s, 3H), 2.15 (s, 3H). ^{13}C-NMR (125 MHz, $CDCl_3$): δ = 162.1 (C_q), 147.3 (C_q), 145.8 (C_q), 144.3 (C_q), 140.1 (CH),

134.5 (C_q), 129.2 (CH), 128.3 (CH), 127.8 (CH), 127.4 (CH), 125.6 (C_q), 125.4 (CH), 125.1 (CH), 111.3 (CH), 20.3 (CH_3), 12.7 (CH_3). IR (ATR): 1659, 1558, 1488, 1434, 1258, 851, 767, 701 cm^{-1}. MS (EI) *m/z* (relative intensity): 292 (35) $[M]^+$, 276 (20), 198 (100), 181 (42), 170 (40), 78 (70). HR-MS (EI) *m/z* calc. for $C_{18}H_{16}N_2O_2$ $[M]^+$: 292.1212, found: 292.1200. The analytical data correspond with those reported in the literature.[2]

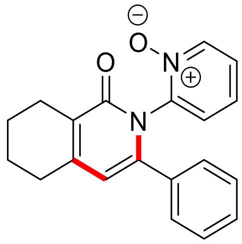

2-[1-Oxo-3-phenyl-5,6,7,8-tetrahydroisoquinolin-2(1*H*)-yl]-pyridine-1-oxide (3ma)

The general procedure **A** was followed using alkene **1m** (109 mg, 0.50 mmol) and alkyne **2a** (102 mg, 1.00 mmol). Purification by column chromatography on silica gel (CH_2Cl_2/acetone 2:1) yielded **3ma** (87.0 mg, 55%) as a colorless oil. ^{1}H-NMR (400 MHz, $CDCl_3$): δ = 8.19 (dd, *J* = 6.3, 0.8 Hz, 1H), 7.35–7.30 (m, 2H), 7.24–7.16 (m, 3H), 7.15–7.10 (m, 1H), 7.09–7.02 (m, 2H), 6.05 (s, 1H), 2.66–2.50 (m, 4H), 1.83–1.73 (m, 4H). ^{13}C-NMR (101 MHz, $CDCl_3$): δ = 161.9 (C_q), 148.5 (C_q), 145.7 (C_q), 144.3 (C_q), 140.1 (CH), 134.7 (C_q), 129.2 (CH), 128.4 (CH), 127.8 (CH), 127.5 (CH), 126.7 (C_q), 125.5 (CH), 125.2 (CH), 110.2 (CH), 29.7 (CH_2), 23.5 (CH_2), 22.1 (CH_2), 22.0 (CH_2). IR (ATR): 3021, 1655, 1481, 1431, 1249, 905, 840, 728 cm^{-1}. MS (EI) *m/z* (relative intensity): 318 (30) $[M]^+$, 301 (10), 224 (100), 195 (20), 181 (60), 78 (60). HR-MS (EI) *m/z* calc. for $C_{20}H_{18}N_2O_2$ $[M]^+$: 318.1368, found: 318.1363. The analytical data correspond with those reported in the literature.[2]

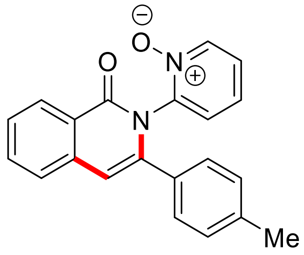

2-[1-Oxo-3-(*p*-tolyl)isoquinolin-2(1*H*)-yl]-pyridine-1-oxide (3ab)

The general procedure **A** was followed using benzamide **1a** (107 mg, 0.50 mmol) and alkyne **2b** (116 mg, 1.00 mmol). Purification by column chromatography on silica gel (CH_2Cl_2/acetone 2:1) yielded **3ab** (164 mg, 99%) as a white solid. M. p.: 232–234 °C. ^{1}H-NMR (400 MHz,

$CDCl_3$): δ = 8.42 (d, J = 8.0 Hz, 1H), 8.22 (d, J = 6.2 Hz, 1H), 7.69 (ddd, J = 8.0, 7.2, 1.4 Hz, 1H), 7.56–7.52 (m, 1H), 7.48 (ddd, J = 8.2, 7.2, 1.2 Hz, 1H), 7.29 (d, J = 8.1 Hz, 2H), 7.19–7.10 (m, 2H), 7.10–7.04 (m, 1H), 7.02 (dd, J = 7.8, 0.7 Hz, 2H), 6.58 (s, 1H), 2.27 (s, 3H). ^{13}C-NMR (101 MHz, $CDCl_3$): δ = 162.2 (C_q), 145.8 (C_q), 142.9 (C_q), 140.2 (CH), 139.2 (C_q), 137.3 (C_q), 133.4 (CH), 132.2 (C_q), 129.1 (CH), 128.5 (CH), 127.9 (CH), 127.8 (CH), 127.1 (CH), 126.4 (CH), 125.5 (CH), 125.2 (CH), 125.0 (C_q), 108.1 (CH), 21.4 (CH_3). IR (ATR): 3066, 1657, 1621, 1481, 1422, 1261, 1141, 815, 759 cm^{-1}. MS (EI) *m/z* (relative intensity): 328.1 (18) $[M]^+$, 312.1 (100), 283.1 (90), 269.1 (29), 195.1 (50), 78.0 (50).. HR-MS (EI) *m/z* calc. for $C_{21}H_{16}N_2O_2$ $[M]^+$: 328.1212, found: 328.1210. The analytical data correspond with those reported in the literature.[2]

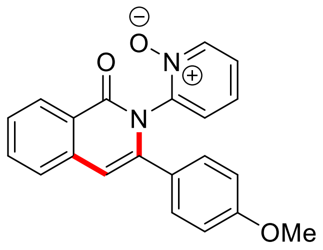

2-[3-(4-Methoxyphenyl)-1-oxoisoquinolin-2(1*H*)-yl]-pyridine 1-oxide (3ac)

The general procedure **A** was followed using benzamide **1a** (107 mg, 0.50 mmol) and alkyne **2c** (132 mg, 1.00 mmol). Purification by column chromatography on silica gel (CH_2Cl_2/acetone 3:1) yielded **3ac** (123 mg, 71%) as a white solid. M. p.: 232–233 °C. ^{1}H-NMR (300 MHz, $CDCl_3$): δ = 8.41 (dd, J = 8.0, 0.5 Hz, 1H), 8.21 (d, J = 6.2 Hz, 1H), 7.68 (ddd, J = 8.0, 7.1, 1.4 Hz, 1H), 7.56–7.51 (m, 1H), 7.48 (ddd, J = 8.3, 7.1, 1.2 Hz, 1H), 7.36–7.30 (m, 2H), 7.20–7.04 (m, 3H), 6.78–6.66 (m, 2H), 6.57 (s, 1H), 3.74 (s, 3H). ^{13}C-NMR (126 MHz, $CDCl_3$): δ = 162.1 (C_q), 160.1 (C_q), 145.9 (C_q), 142.5 (C_q), 140.2 (CH), 137.3 (C_q), 133.4 (CH), 129.4 (CH), 128.4 (CH), 127.8 (CH), 127.4 (C_q), 127.0 (CH), 126.3 (CH), 125.4 (CH), 125.2 (CH), 124.9 (C_q), 113.7 (CH), 107.9 (CH), 55.4 (CH_3). IR (ATR): 3068, 1668, 1625, 1511, 1249, 1028, 761, 531 cm^{-1}. MS (EI) *m/z* (relative intensity): 344.1 (26) $[M]^+$, 328.1 (74), 299.1 (61), 211.1 (100), 78.0 (87). HR-MS (EI) *m/z* calc. for $C_{21}H_{16}N_2O_3$ $[M]^+$: 344.1161, found: 344.1167. The analytical data correspond with those reported in the literature.[2]

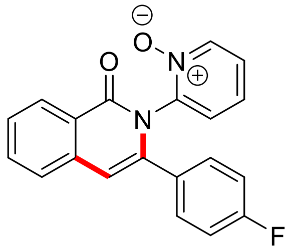

2-[3-(4-Fluorophenyl)-1-oxoisoquinolin-2(1*H*)-yl]-pyridine 1-oxide (3ad)

The general procedure **A** was followed using benzamide **1a** (107 mg, 0.50 mmol) and alkyne **2d** (120 mg, 1.00 mmol). Purification by column chromatography on silica gel (CH_2Cl_2/acetone 3:1) yielded **3ad** (156 mg, 94%) as a white solid. M. p.: 244–245 °C. ^{1}H-NMR (400 MHz, $CDCl_3$): δ = 8.42 (dd, J = 8.0, 1.2 Hz, 1H), 8.20 (dd, J = 6.3, 0.7 Hz, 1H), 7.70 (ddd, J = 8.0, 7.2, 1.4 Hz, 1H), 7.55 (dd, J = 7.8, 0.8 Hz, 1H), 7.51 (ddd, J = 8.3, 7.2, 1.2 Hz, 1H), 7.45–7.36 (m, 2H), 7.21–7.14 (m, 2H), 7.14–7.07 (m, 1H), 6.96–6.87 (m, 2H), 6.58 (s, 1H). ^{13}C-NMR (101 MHz, $CDCl_3$): δ = 163.2 (d, $^1J_{C–F}$ = 249.6 Hz, C_q), 162.1 (C_q), 145.6 (C_q), 141.7 (C_q), 140.3 (CH), 137.1 (C_q), 133.6 (CH), 131.1 (d, $^4J_{C–F}$ = 3.4 Hz, C_q), 130.2 (d, $^3J_{C–F}$ = 8.4 Hz, CH), 128.6 (CH), 127.9 (CH), 127.4 (CH), 126.5 (CH), 125.7 (CH), 125.3 (CH), 125.1 (C_q), 115.5 (d, $^2J_{C–F}$ = 21.8 Hz, CH), 108.4 (CH). ^{19}F-NMR (376 MHz, $CDCl_3$) δ = –(111.30–111.40) (m). IR (ATR): 3076, 1660, 1481, 1262, 1141, 891, 761, 692 cm^{-1}. MS (EI) *m/z* (relative intensity): 332 (20) [M]$^+$, 316 (35), 287 (50), 212 (36), 199 (100), 183 (38), 78.0 (80). HR-MS (EI) *m/z* calc. for $C_{20}H_{13}FN_2O_2$ [M]$^+$: 332.0961, found: 332.0964. The analytical data correspond with those reported in the literature.[2]

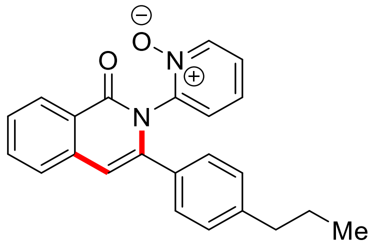

2-[1-Oxo-3-(4-propylphenyl)isoquinolin-2(1*H*)-yl]pyridine 1-oxide (3ae)

The general procedure **A** was followed using benzamide **1a** (107 mg, 0.50 mmol) and alkyne **2e** (148 mg, 1.00 mmol). Purification by column chromatography on silica gel (CH_2Cl_2/acetone 3:1) yielded **3ae** (154 mg, 87%) as a white solid. M. p.: 167–169 °C. ^{1}H-NMR (400 MHz, $CDCl_3$): δ = 8.42 (dd, J = 8.0, 0.6 Hz, 1H), 8.20 (dd, J = 6.4, 0.9 Hz, 1H), 7.68 (ddd, J = 7.9, 7.2, 1.4 Hz, 1H), 7.54 (dd, J = 7.8, 0.6 Hz, 1H), 7.48 (ddd, J = 8.3, 7.2, 1.2 Hz, 1H), 7.32–7.27

(m, 2H), 7.17–7.09 (m, 2H), 7.09–6.98 (m, 3H), 6.60 (s, 1H), 2.54–2.46 (m, 2H), 1.56 (sept, *J* = 7.4 Hz, 2H), 0.85 (t, *J* = 7.3 Hz, 3H). ^{13}C-NMR (101 MHz, $CDCl_3$): δ = 162.2 (C_q), 145.9 (C_q), 143.9 (C_q), 142.9 (C_q), 140.2 (CH), 137.3 (C_q), 133.4 (CH), 132.3 (C_q), 128.5 (CH), 128.4 (CH), 127.9 (CH), 127.8 (CH), 127.1 (CH), 126.4 (CH), 125.4 (CH), 125.1 (CH), 125.0 (C_q), 108.0 (CH), 37.7 (CH_2), 24.3 (CH_2), 13.7 (CH_3). IR (ATR): 1660, 1481, 1377, 1261, 889, 758, 692, 517 cm^{-1}. MS (EI) *m/z* (relative intensity): 356 (20) [M]$^+$, 340 (40), 311 (45), 223 (100), 78.0 (63). HR-MS (EI) *m/z* calc. for $C_{23}H_{20}N_2O_2$ [M]$^+$: 356.1525, found: 356.1532. The analytical data correspond with those reported in the literature.[2]

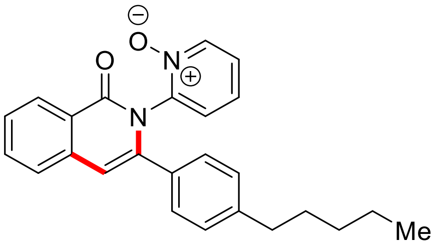

2-[1-Oxo-3-(4-pentylphenyl)isoquinolin-2(1*H*)-yl]-pyridine-1-oxide (3af)

The general procedure **A** was followed using benzamide **1a** (107 mg, 0.50 mmol) and alkyne **2f** (172 mg, 1.00 mmol). Purification by column chromatography on silica gel (CH_2Cl_2/acetone 5:1) yielded **3af** (126 mg, 66%) as a yellow oil. ^{1}H-NMR (300 MHz, $CDCl_3$): δ = 8.46–8.39 (m, 1H), 8.21 (dd, *J* = 6.3, 0.6 Hz, 1H), 7.69 (ddd, *J* = 8.3, 7.1, 1.4 Hz, 1H), 7.54 (d, *J* = 7.8 Hz, 1H), 7.49 (ddd, *J* = 8.3, 7.1, 1.2 Hz, 1H), 7.32–7.26 (m, 2H), 7.19–7.10 (m, 2H), 7.10–7.04 (m, 1H), 7.02 (dd, *J* = 8.3, 1.9 Hz, 2H), 6.60 (s, 1H), 2.52 (t, *J* = 7.5 Hz, 2H), 1.53 (p, *J* = 7.5 Hz, 2H), 1.32–1.17 (m, 4H), 0.86 (t, *J* = 7.0 Hz, 3H). ^{13}C-NMR (126 MHz, $CDCl_3$): δ = 162.1 (C_q), 145.8 (C_q), 144.1 (C_q), 142.8 (C_q), 140.1 (CH), 137.2 (C_q), 133.4 (CH), 132.2 (C_q), 128.5 (CH), 128.3 (CH), 127.9 (CH), 127.8 (CH), 127.0 (CH), 126.3 (CH), 125.3 (CH), 125.1 (CH), 125.0 (C_q), 108.0 (CH), 35.7 (CH_2), 31.5 (CH_2), 30.9 (CH_2), 22.6 (CH_2), 14.2 (CH_3). IR (ATR): 3057, 1666, 1481, 1430, 1260, 1023, 822, 756 cm^{-1}. MS (EI) *m/z* (relative intensity): 384 (25) [M]$^+$, 368 (75), 339 (40), 311 (40), 251 (100), 78.0 (62). HR-MS (EI) *m/z* calc. for $C_{25}H_{24}N_2O_2$ [M]$^+$: 384.1838, found: 384.1840.

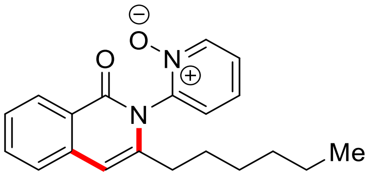

2-[3-Hexyl-1-oxoisoquinolin-2(1*H*)-yl]-pyridine-1-oxide (3ag)

The general procedure **A** was followed using benzamide **1a** (107 mg, 0.50 mmol) and alkyne **2g** (110 mg, 1.00 mmol). Purification by column chromatography on silica gel (CH_2Cl_2/acetone 3:1) yielded **3ag** (102 mg, 63%) as a white solid. M. p.: 138–140 °C. ^{1}H-NMR (400 MHz, $CDCl_3$): δ = 8.40 (dd, J = 5.0, 2.9 Hz, 1H), 8.35–8.31 (m, 1H), 7.64 (ddd, J = 8.3, 7.1, 1.4 Hz, 1H), 7.50–7.44 (m, 2H), 7.44–7.36 (m, 3H), 6.44 (s, 1H), 2.43–2.09 (m, 2H), 1.62–1.45 (m, 2H), 1.31–1.11 (m, 6H), 0.84 (t, J = 6.9 Hz, 3H). ^{13}C-NMR (101 MHz, $CDCl_3$): δ = 162.8 (C_q), 144.6 (C_q), 142.7 (C_q), 140.8 (CH), 137.6 (C_q), 133.3 (CH), 128.3 (CH), 128.2 (CH), 126.5 (CH), 126.1 (CH), 125.9 (CH), 125.6 (CH), 124.6 (C_q), 105.1 (CH), 32.1 (CH_2), 31.5 (CH_2), 28.9 (CH_2), 27.7 (CH_2), 22.5 (CH_2), 14.1 (CH_3). IR (ATR): 2927, 1630, 1426, 1396, 1265, 876, 570, 493 cm^{-1}. MS (EI) *m/z* (relative intensity): 328 (30) [M]$^+$, 306 (48), 251 (45), 234 (100), 78.0 (61). HR-MS (EI) *m/z* calc. for $C_{20}H_{22}N_2O_2$ [M]$^+$: 322.1681, found: 322.1684.

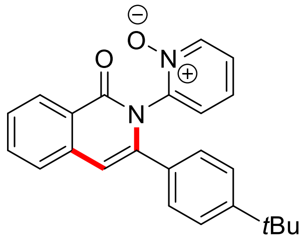

2-{3-[4-(*tert*-Butyl)phenyl]-1-oxoisoquinolin-2(1*H*)-yl}-pyridine-1-oxide (3ah)

The general procedure **A** was followed using benzamide **1a** (107 mg, 0.50 mmol) and alkyne **2h** (158 mg, 1.00 mmol). Purification by column chromatography on silica gel (CH_2Cl_2/acetone 4:1) yielded **3ah** (119 mg, 51%) as a white solid. M. p. = 162–163 °C. ^{1}H-NMR (400 MHz, $CDCl_3$): δ = 8.41 (dd, J = 8.0, 0.7 Hz, 1H), 8.28–8.17 (m, 1H), 7.69 (ddd, J = 8.2, 7.1, 1.2 Hz, 1H), 7.54 (dd, J = 8.0, 1.1 Hz, 1H), 7.49 (ddd, J = 8.1, 7.1, 1.1 Hz, 1H), 7.35–7.28 (m, 2H), 7.25–7.20 (m, 2H), 7.18–7.12 (m, 2H), 7.12–7.04 (m, 1H), 6.60 (s, 1H), 1.24 (s, 9H). ^{13}C-NMR (126 MHz, $CDCl_3$): δ = 162.1 (C_q), 152.3 (C_q), 145.7 (C_q), 142.8 (C_q), 140.1 (CH), 137.3 (C_q), 133.4 (CH), 132.0 (C_q), 128.5 (CH), 127.9 (CH), 127.7 (CH), 127.0 (CH), 126.3 (CH), 125.4 (CH), 125.2 (CH), 125.1 (CH), 125.0 (C_q), 108.1 (CH), 34.8 (C_q), 31.3 (CH_3). IR (ATR): 3056, 1678, 1621, 1478, 1432, 1272, 876, 758 cm^{-1}. MS (EI) *m/z* (relative intensity): 370 (25) [M]$^+$, 269 (10), 237 (100), 194 (25), 78 (40), 57 (10). HR-MS (EI) *m/z* calc. for $C_{24}H_{22}N_2O_2$ [M]$^+$: 370.1681, found: 370.1680. The analytical data correspond with those reported in the literature.[2]

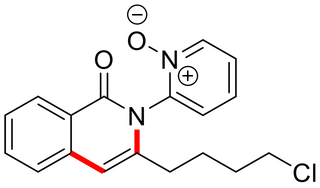

2-[3-(4-Chlorobutyl)-1-oxoisoquinolin-2(1*H*)-yl]-pyridine-1-oxide (3ai)

The general procedure **A** was followed using benzamide **1a** (107 mg, 0.50 mmol) and alkyne **2i** (117 mg, 1.00 mmol). Purification by column chromatography on silica gel (CH_2Cl_2/acetone 3:1) yielded **3ai** (107 mg, 65%) as a white solid. M. p.: 169–170 °C. ^{1}H-NMR (300 MHz, $CDCl_3$): δ = 8.39 (dd, *J* = 4.9, 3.0 Hz, 1H), 8.36–8.28 (m, 1H), 7.65 (ddd, *J* = 8.3, 7.1, 1.4 Hz, 1H), 7.52–7.45 (m, 2H), 7.45–7.41 (m, 1H), 7.41–7.36 (m, 2H), 6.45 (s, 1H), 3.55–3.38 (m, 2H), 2.44–2.15 (m, 2H), 1.82–1.59 (m, 4H). ^{13}C-NMR (125 MHz, $CDCl_3$): δ = 162.6 (C_q), 144.3 (C_q), 141.7 (C_q), 140.7 (CH), 137.3 (C_q), 133.3 (CH), 128.2 (CH), 128.1 (CH), 126.6 (CH), 126.2 (CH), 125.8 (CH), 125.7 (CH), 124.6 (C_q), 105.3 (CH), 44.6 (CH_2), 31.9 (CH_2), 31.4 (CH_2), 24.9 (CH_2). IR (ATR): 3092, 1631, 1561, 1490, 1424, 1259, 823, 768 cm^{-1}. MS (EI) *m/z* (relative intensity): 328 (25) [M]$^+$, 311 (40), 251 (50), 234 (100), 171 (30), 78.0 (72). HR-MS (EI) *m/z* calc. for $C_{18}H_{17}ClN_2O_2$ [M]$^+$: 328.0979, found: 328.0971. The analytical data correspond with those reported in the literature.[2]

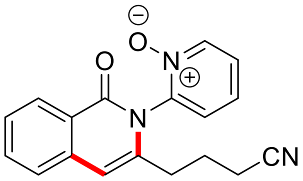

2-[3-(3-Cyanobutyl)-1-oxoisoquinolin-2(1*H*)-yl]-pyridine-1-oxide (3aj)

The general procedure **A** was followed using benzamide **1a** (107 mg, 0.50 mmol) and alkyne **2j** (117 mg, 1.00 mmol). Purification by column chromatography on silica gel (CH_2Cl_2/acetone 3:1) yielded **3aj** (120 mg, 79%) as a white solid. M. p.: 176–177 °C. ^{1}H-NMR (300 MHz, $CDCl_3$): δ = 8.42–8.36 (m, 1H), 8.33 (dd, *J* = 8.1, 1.3 Hz, 1H), 7.67 (ddd, *J* = 8.3, 7.1, 1.4 Hz, 1H), 7.53–7.47 (m, 2H), 7.46–7.44 (m, 1H), 7.44–7.37 (m, 2H), 6.47 (s, 1H), 2.56–2.43 (m, 2H), 2.42–2.26 (m, 2H), 1.99–1.74 (m, 2H). ^{13}C-NMR (125 MHz, $CDCl_3$): δ = 162.5 (C_q), 144.1 (C_q), 140.8 (CH), 139.7 (C_q), 136.9 (C_q), 133.5 (CH), 128.3 (CH), 128.0 (CH), 127.0 (CH), 126.5 (CH), 125.9 (CH), 125.9 (CH), 124.8 (C_q), 118.9 (C_q), 106.1 (CH), 31.1 (CH_2), 23.6 (CH_2), 16.7 (CH_2). IR (ATR): 2921, 1630, 1596, 1428, 1247, 890, 766, 751 cm^{-1}. MS (EI)

m/z (relative intensity): 305 (26) [M]$^+$, 265 (37), 249 (100), 234 (68), 171 (63), 78.0 (66). HR-MS (EI) *m/z* calc. for $C_{18}H_{15}N_3O_2$ [M]$^+$: 305.1164, found: 305.1156.

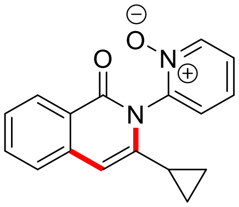

2-[3-Cyclopropyl-1-oxoisoquinolin-2(1*H*)-yl]-pyridine-1-oxide (3ak)

The general procedure **A** was followed using benzamide **1a** (107 mg, 0.50 mmol) and alkyne **2k** (66.1 mg, 1.00 mmol). Purification by column chromatography on silica gel (CH_2Cl_2/acetone 3:1) yielded **3ak** (80.2 mg, 57%) as a white solid. M. p.: 224–226 °C. ^{1}H-NMR (300 MHz, $CDCl_3$): δ = 8.40 (dd, J = 5.0, 2.9 Hz, 1H), 8.34 (dd, J = 8.0, 0.6 Hz, 1H), 7.63 (ddd, J = 8.2, 7.1, 1.4 Hz, 1H), 7.51–7.44 (m, 2H), 7.44–7.40 (m, 1H), 7.40–7.35 (m, 2H), 6.38 (s, 1H), 1.59–1.44 (m, 1H), 1.00–0.89 (m, 1H), 0.70–0.46 (m, 3H). ^{13}C-NMR (126 MHz, $CDCl_3$): δ = 162.5 (C_q), 145.1 (C_q), 143.4 (C_q), 140.5 (CH), 137.4 (C_q), 133.2 (CH), 128.3 (CH), 128.0 (CH), 126.6 (CH), 125.9 (CH), 125.9 (CH), 125.5 (CH), 124.8 (C_q), 104.9 (CH), 13.3 (CH), 7.4 (CH_2), 5.4 (CH_2). IR (ATR): 1666, 1632, 1592, 1428, 1392, 1266, 758, 728 cm^{-1}. MS (EI) *m/z* (relative intensity): 278 (40) [M]$^+$, 261 (85), 234 (80), 193 (50), 145 (55), 78 (100). HR-MS (EI) *m/z* calc. for $C_{17}H_{14}N_2O_2$ [M]$^+$: 278.1055, found: 278.1057. The analytical data correspond with those reported in the literature.[2]

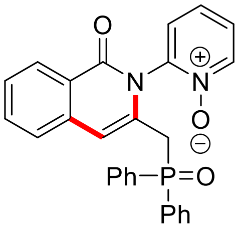

2-{3-[(Diphenylphosphoryl)methyl]-1-oxoisoquinolin-2(1*H*)-yl}pyridine 1-oxide (5)

The general procedure **B** was followed using benzamide **1a** (107 mg, 0.50 mmol) and allene **4** (144 mg, 0.60 mmol). Purification by column chromatography on silica gel (CH_2Cl_2/acetone 1:1 to CH_2Cl_2/MeOH 9:1) yielded **5** (206 mg, 455 µmol, 63%) as a white solid. M. p.: 129–130 °C. ^{1}H-NMR (600 MHz, $CDCl_3$): δ = 8.36–8.26 (m, 2H), 7.76–7.70 (m, 2H), 7.60–7.54 (m, 2H), 7.51–7.44 (m, 6H), 7.43–7.35 (m, 3H), 7.33–7.30 (m, 1H), 7.29–7.25 (m, 2H), 6.36 (d, J = 3.1 Hz, 1H), 3.74–3.61 (m, 1H), 3.16–3.01 (m, 1H). ^{13}C-NMR (126 MHz, $CDCl_3$): δ =

162.6 (C_q), 143.8 (C_q), 140.0 (CH), 136.7 (C_q), 133.3 (CH), 132.9 (d, $^2J_{C–P}$ = 7.9 Hz, C_q), 132.3 (d, $^4J_{C–P}$ = 2.7 Hz, CH), 132.2 (d, $^4J_{C–P}$ = 2.7 Hz, CH), 132.1 (d, $^1J_{C–P}$ = 100.1 Hz, C_q), 131.4 (d, $^2J_{C–P}$ = 9.1 Hz, CH), 131.1 (d, $^1J_{C–P}$ = 101.2 Hz, C_q), 130.6 (d, $^2J_{C–P}$ = 9.4 Hz, CH), 130.3 (CH), 128.9 (d, $^3J_{C–P}$ = 4.2 Hz, CH), 128.8 (d, $^3J_{C–P}$ = 4.2 Hz, CH), 128.2 (CH), 127.0 (CH), 126.1 (CH), 125.9 (CH), 125.8 (CH), 124.9 (C_q), 108.9 (d, $^3J_{C–P}$ = 7.0 Hz, CH), 34.5 (d, $^1J_{C–P}$ = 65.6 Hz, CH_2). $^{31}P\{^1H\}$-NMR (162 MHz, $CDCl_3$): δ = 27.6. IR (ATR): 3049, 1676, 1628, 1426, 1266, 1195, 720, 497 cm^{-1}. MS (EI) *m/z* (relative intensity): 452 (25) $[M]^+$, 453 (45), 319 (10), 251 (28), 234 (100), 201 (80), 77 (40). HR-MS (EI) *m/z* calc. for $C_{27}H_{21}N_2O_3P$ $[M]^+$: 452.1290, found: 452.1292. The analytical data correspond with those reported in the literature.[4]

Cyclic Voltammetry

The cyclic voltammetry measurements were carried out using a Metrohm Autolab PGSTAT204 workstation and following analysis was performed with Nova 2.1 software. For all experiments a glassy-carbon (GC) electrode (3 mm-diameter, disc-electrode) was used as the working electrode and a saturated calomel electrode (SCE) was used as the reference electrode. The measurements were recorded at a scan rate of 100 mVs^{-1}. The operation temperature was 298 K. All solutions were degassed via freeze-pump-thaw method prior to use and nitrogen was bubbled through the solutions for at least 5 min before the experiment was performed. The experiments were performed under inert conditions (constant flow of dry nitrogen).

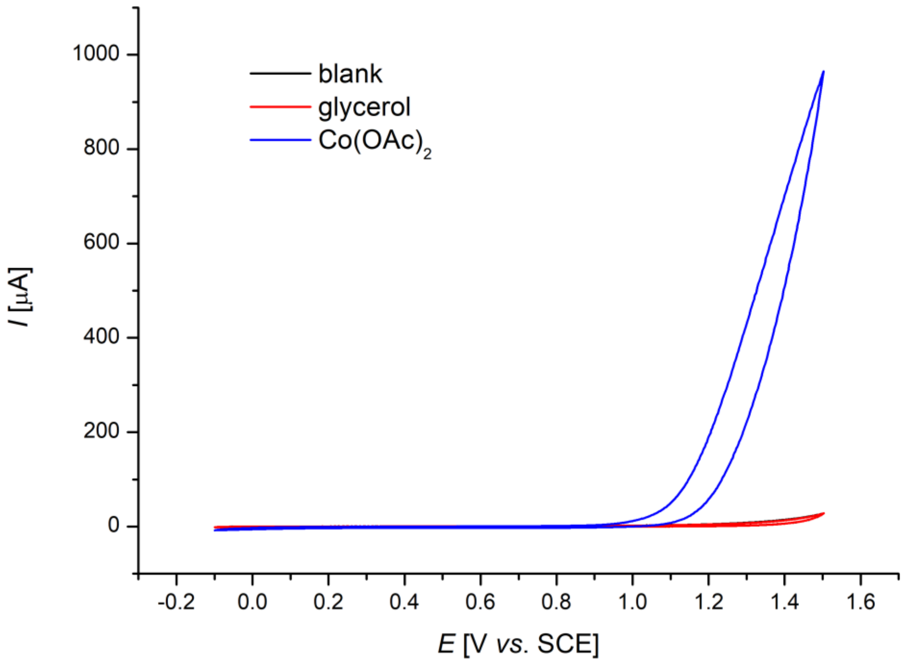

Figure S-4. Cyclic voltammograms at 100 mVs^{-1} using H_2O and NaOAc (0.2 M) as the electrolyte and a GC working electrode. (black) Blank, (red) glycerol (50 mM), (blue) $Co(OAc)_2$ (10 mM).

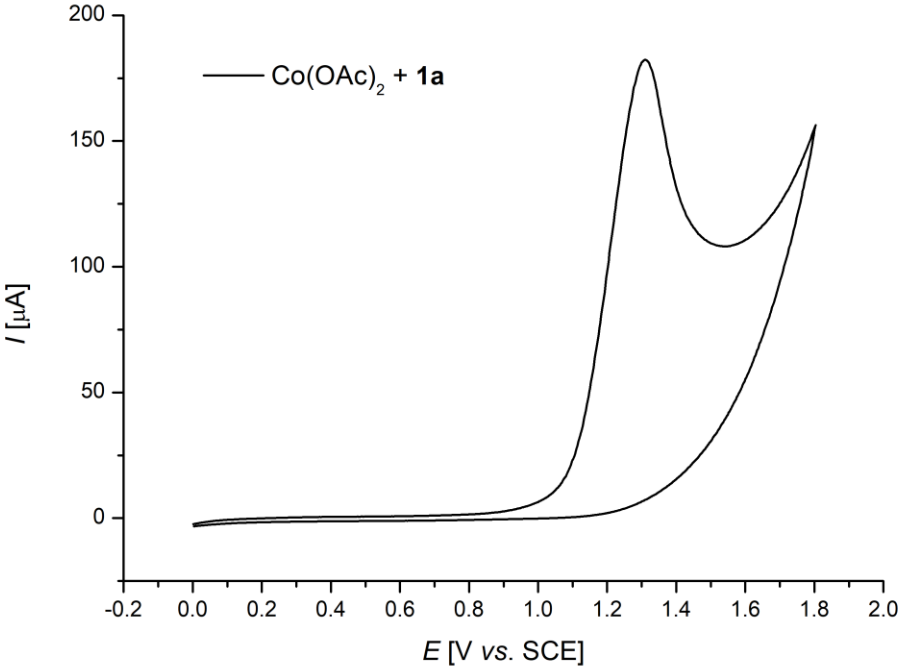

Figure S-5. Cyclic voltammogram at 100 mVs^{-1} using H_2O and NaOAc (0.2 M) as the electrolyte and a GC working electrode. $Co(OAc)_2$ (10 mM) and benzamide **1a** (10 mM).

References

[1] a) R. Mei, H. Wang, S. Warratz, S. A. Macgregor, L. Ackermann, *Chem. Eur. J.* **2016**, *22*, 6759-6763; b) S. S. Lande, C. D. Falk, J. K. Kochi, *J. Inorg. Nucl. Chem.* **1971**, *33*, 4101-4109.

[2] C. Tian, L. Massignan, T. H. Meyer, L. Ackermann, *Angew. Chem. Int. Ed.* **2018**, *57*, 2383-2387.

[3] X.-Q. Hao, C. Du, X. Zhu, P.-X. Li, J.-H. Zhang, J.-L. Niu, M.-P. Song, *Org. Lett.* **2016**, *18*, 3610-3613.

[4] T. H. Meyer, J. C. A. Oliveira, S. C. Sau, N. W. J. Ang, L. Ackermann, *ACS Catal.* **2018**, *8*, 9140-9147.

^{1}H- and ^{13}C-NMR Spectra

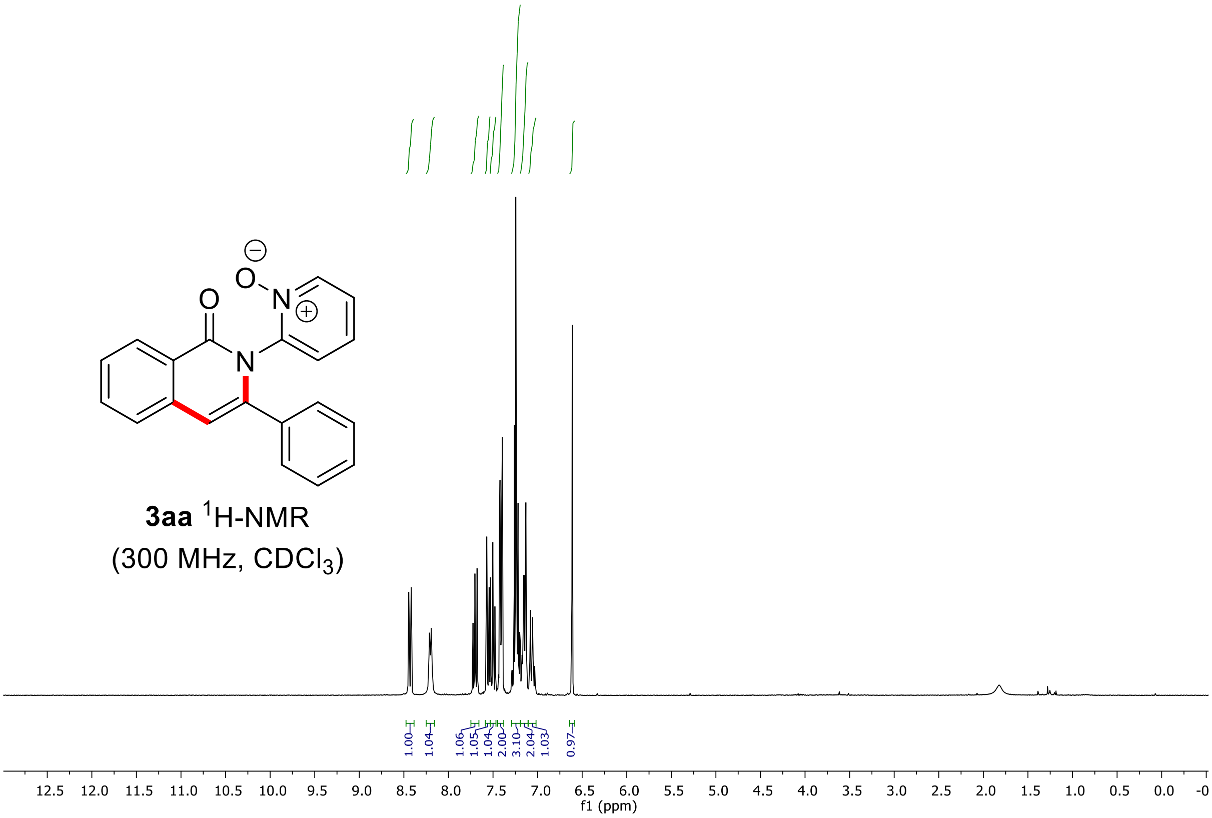

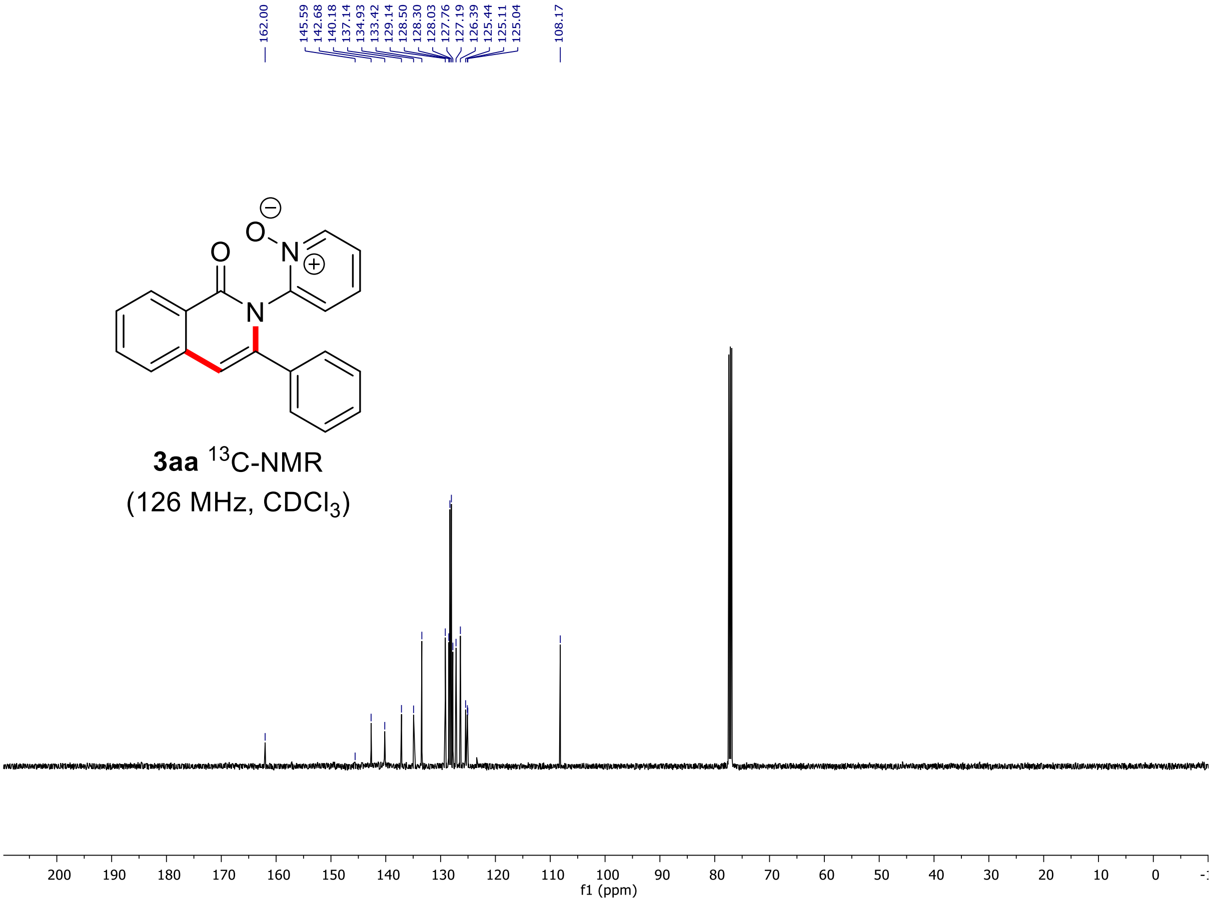

3ba ^{1}H-NMR
(400 MHz, $CDCl_3$)

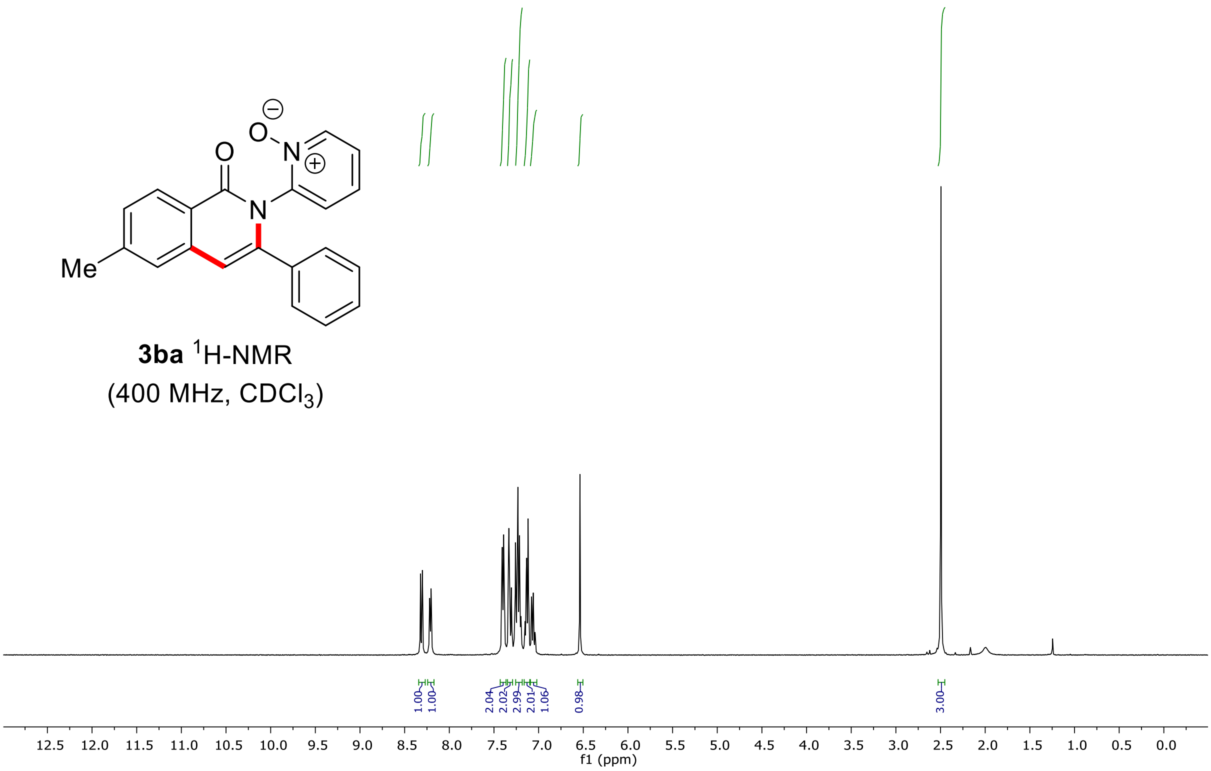

3ba ^{13}C-NMR
(101 MHz, $CDCl_3$)

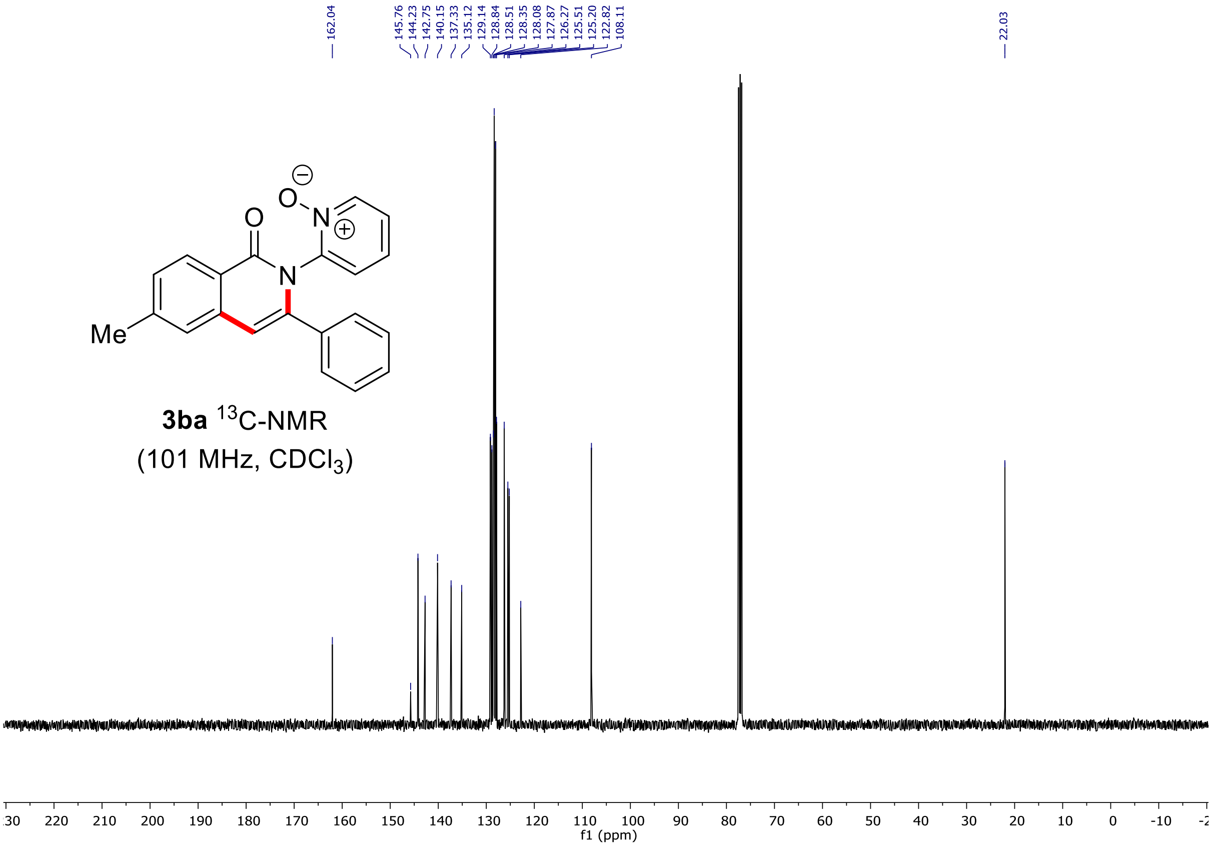

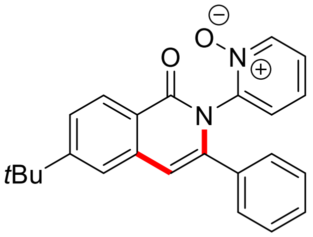

3ca ^{1}H-NMR
(300 MHz, $CDCl_3$)

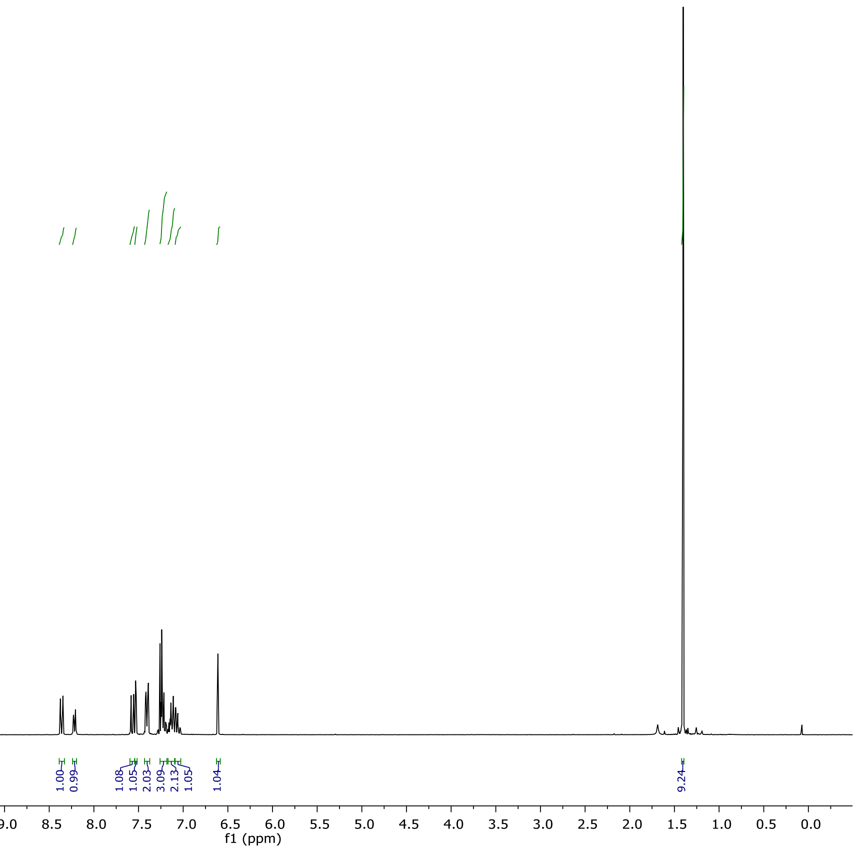

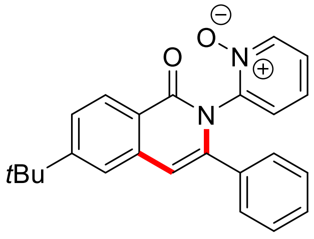

3ca ^{13}C-NMR
(126 MHz, $CDCl_3$)

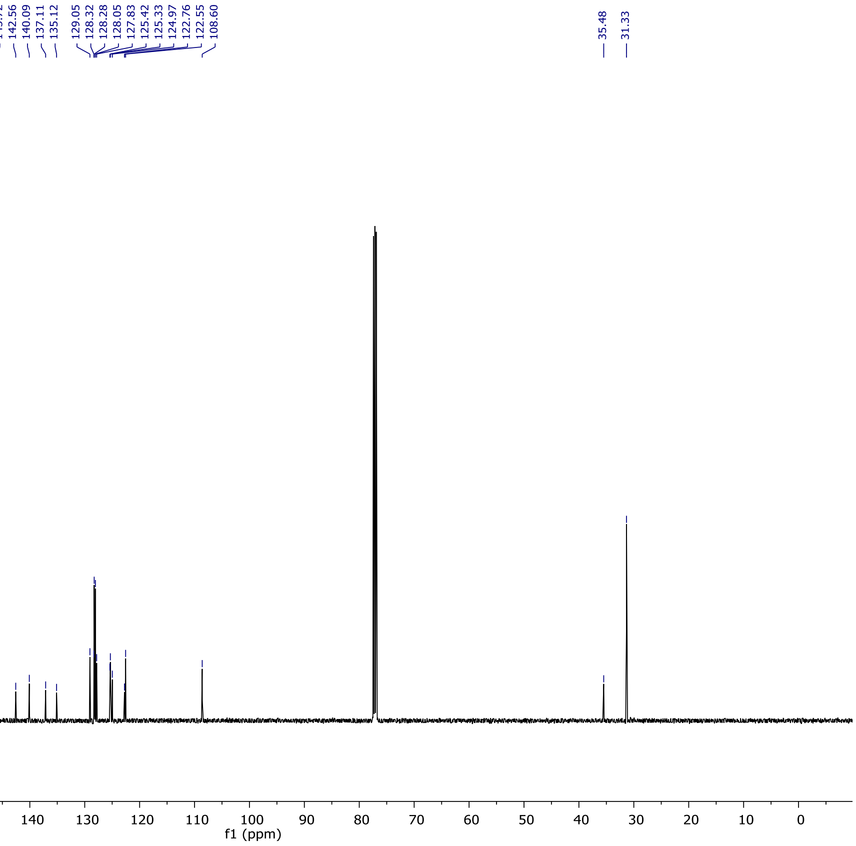

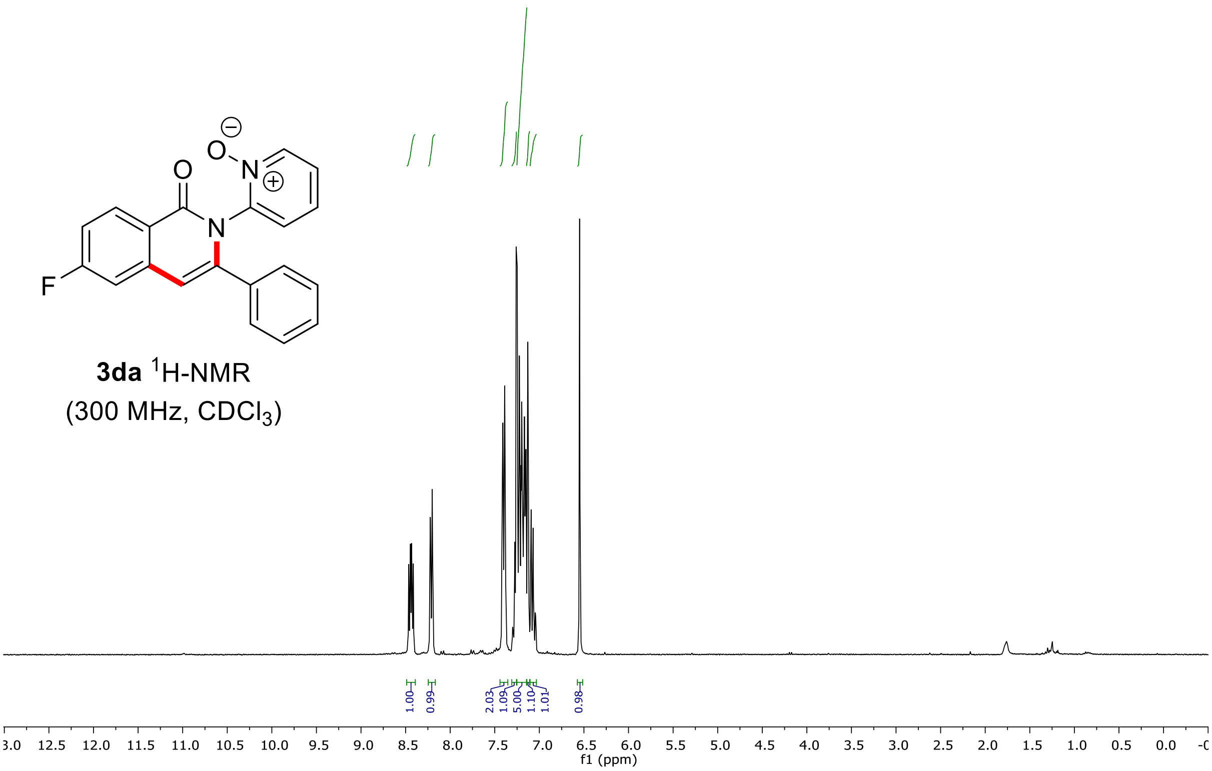
3da 1H-NMR
(300 MHz, CDCl3)
1.00
0.99
2.03
1.09
5.00
1.10
1.01
0.98
f1 (ppm)

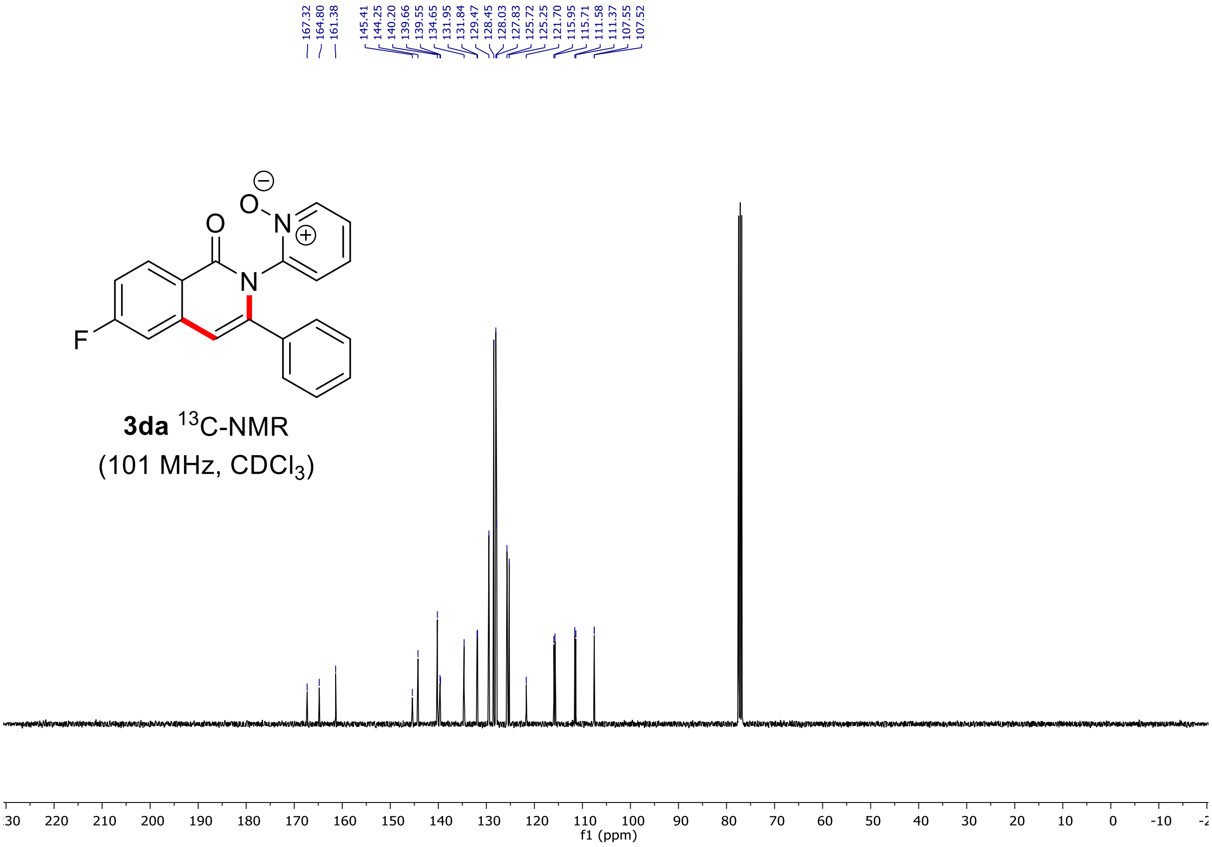
167.32
164.80
161.38
145.41
144.25
140.20
139.66
139.55
134.65
131.95
131.84
129.47
128.45
128.03
127.83
125.72
125.25
121.70
115.95
115.71
111.58
111.37
107.55
107.52
3da 13C-NMR
(101 MHz, CDCl3)
f1 (ppm)

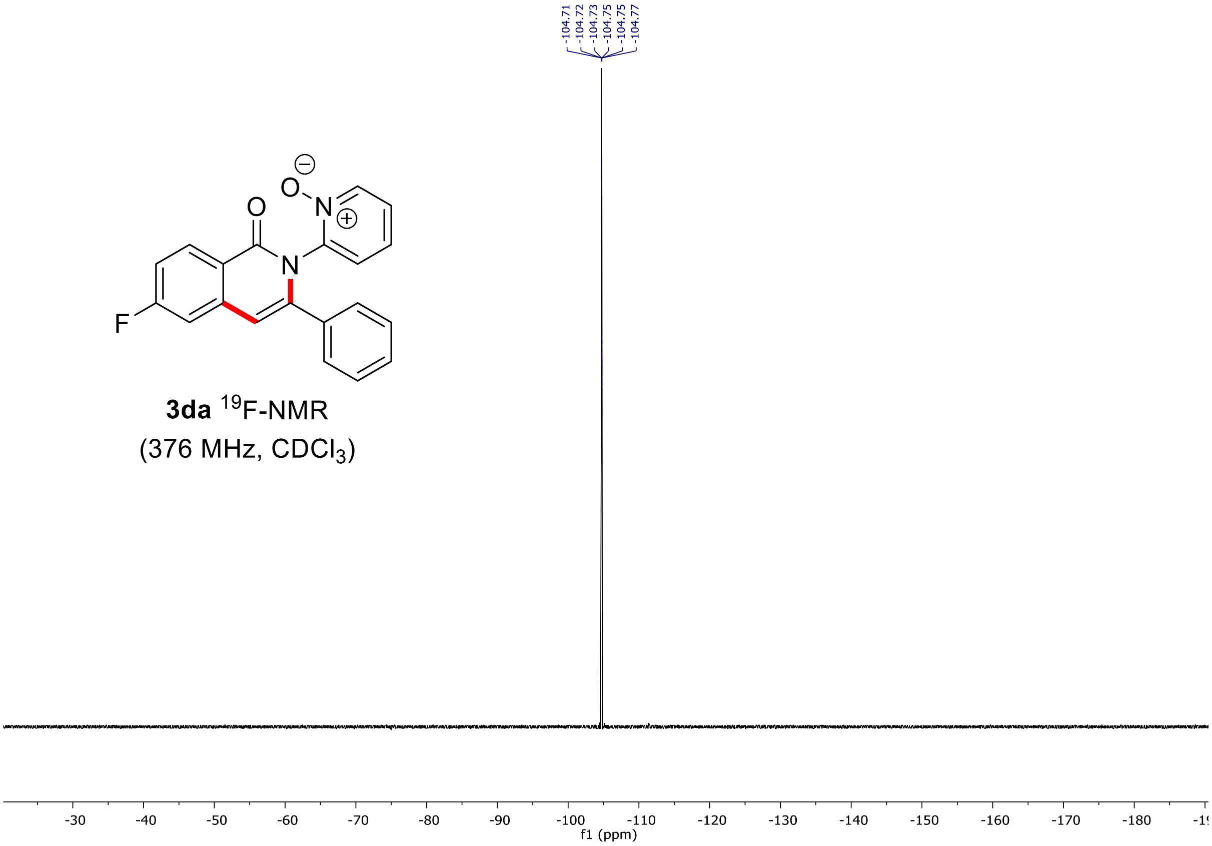
-104.71
-104.72
-104.73
-104.75
-104.75
-104.77
3da ^{19}F-NMR
(376 MHz, $CDCl_3$)
f1 (ppm)

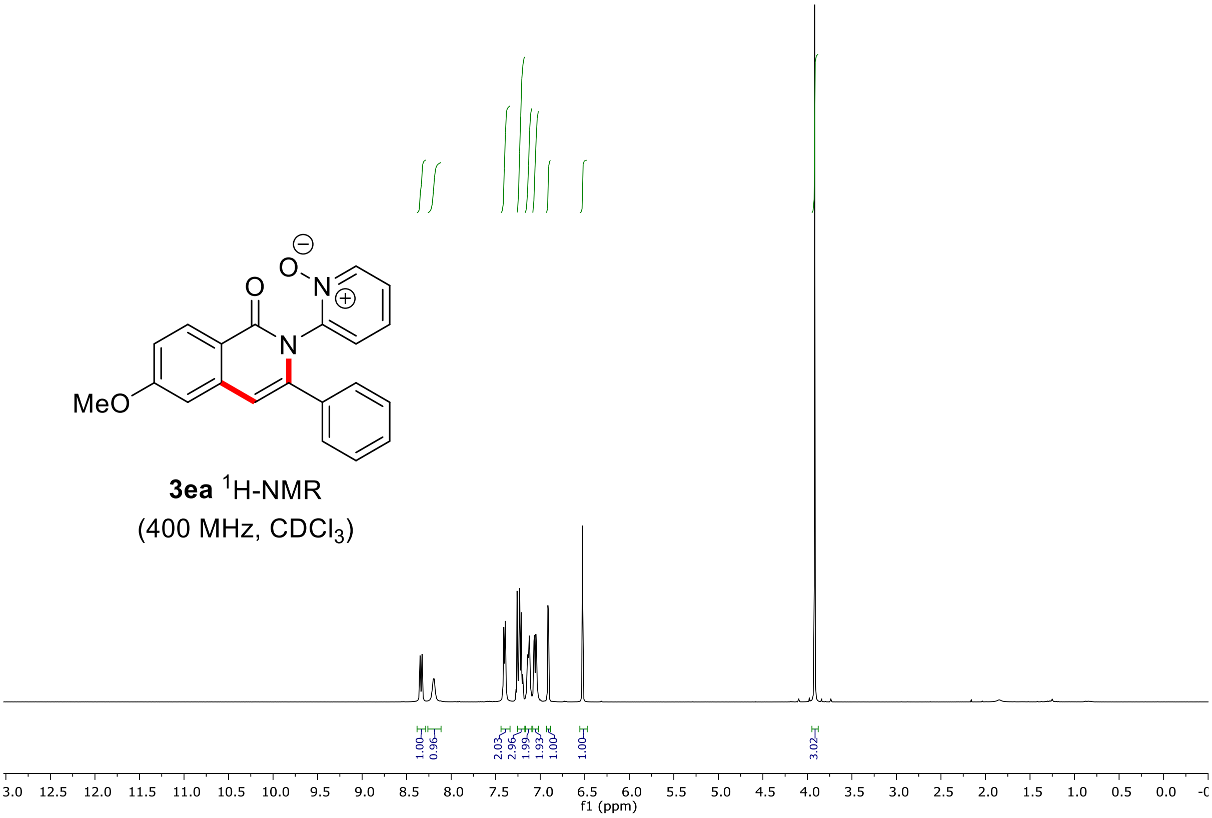
3ea ^{1}H-NMR
(400 MHz, $CDCl_3$)
1.00
0.96
2.03
2.96
1.99
1.93
1.00
1.00
3.02
f1 (ppm)

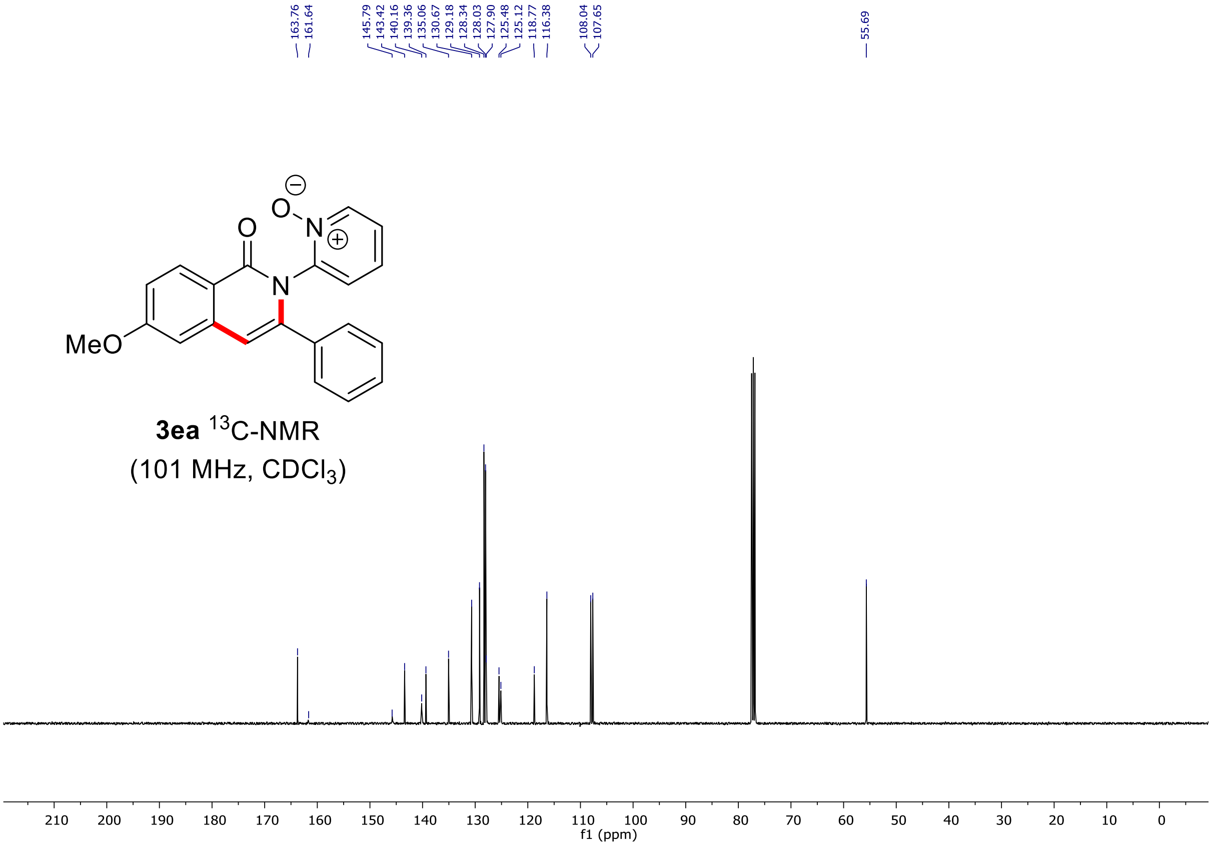
163.76
161.64
145.79
143.42
140.16
139.36
135.06
130.67
129.18
128.34
128.03
127.90
125.48
125.12
118.77
116.38
108.04
107.65
55.69
MeO
3ea 13C-NMR
(101 MHz, CDCl3)
f1 (ppm)

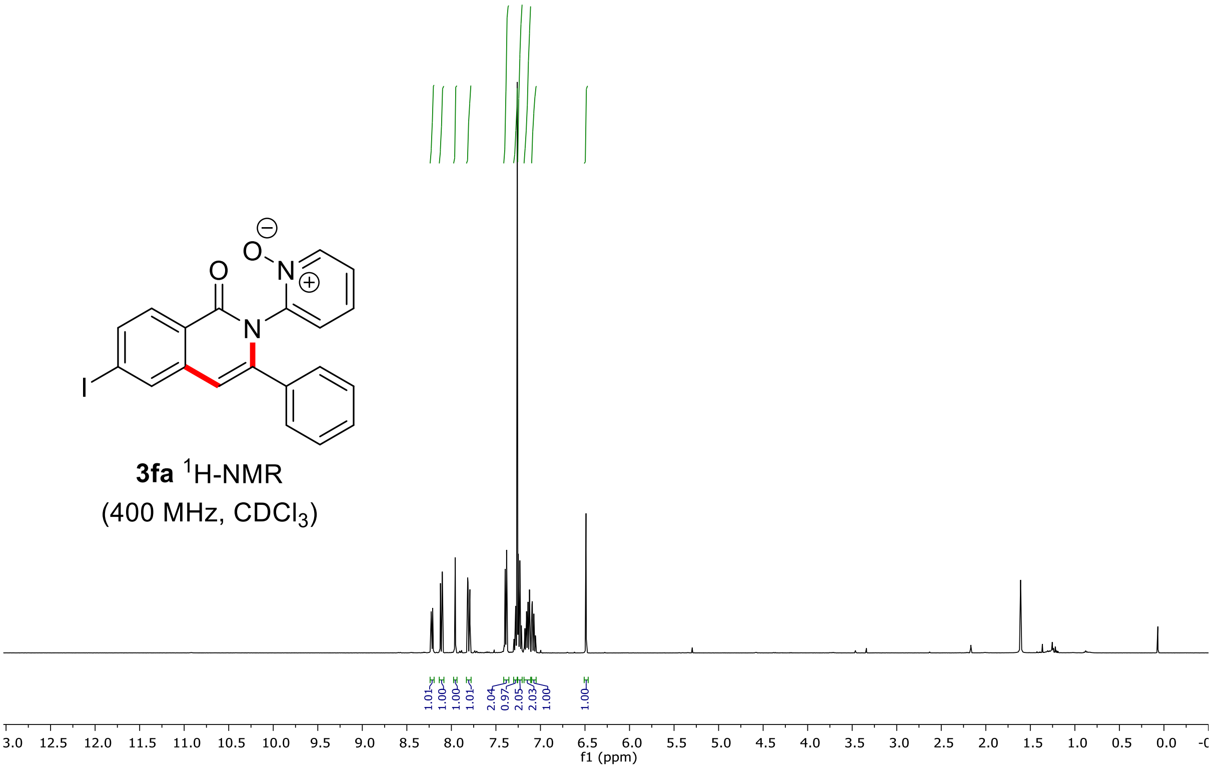
I
3fa 1H-NMR
(400 MHz, CDCl3)
1.01
1.00
1.00
1.01
2.04
0.97
2.05
2.03
1.00
1.00
f1 (ppm)

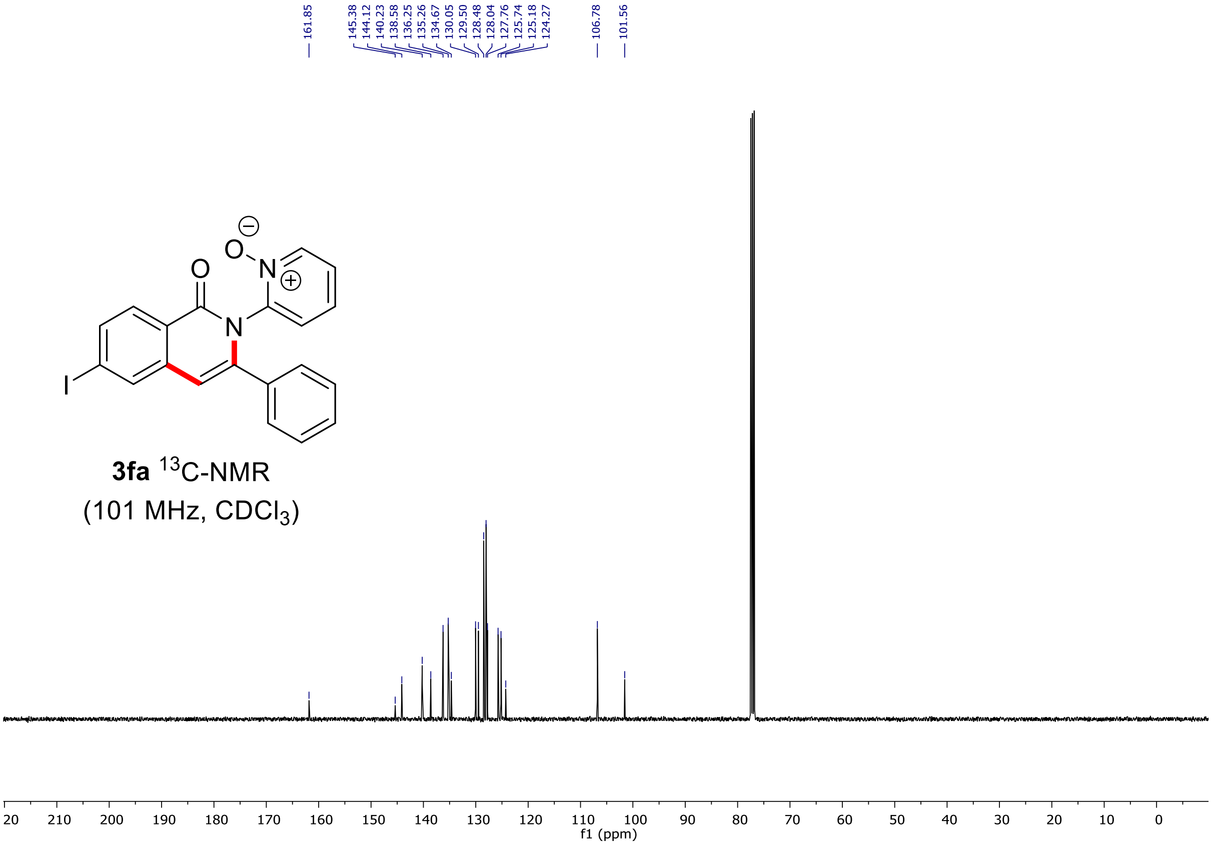

3fa ^{13}C-NMR
(101 MHz, $CDCl_3$)
161.85
145.38
144.12
140.23
138.58
136.25
135.26
134.67
130.05
129.50
128.48
128.04
127.76
125.74
125.18
124.27
106.78
101.56
f1 (ppm)

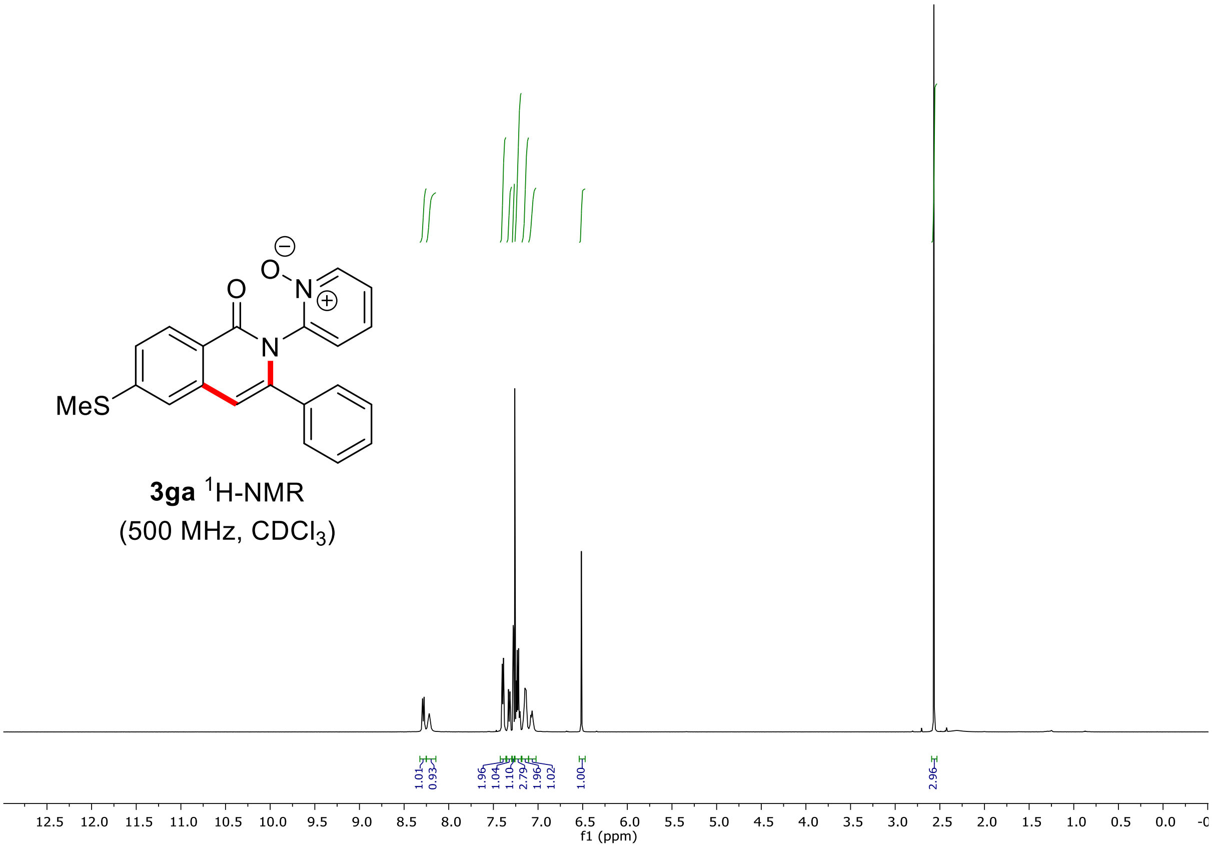

MeS
3ga ^{1}H-NMR
(500 MHz, $CDCl_3$)
1.01
0.93
1.96
1.04
1.10
2.79
1.96
1.02
1.00
2.96
f1 (ppm)

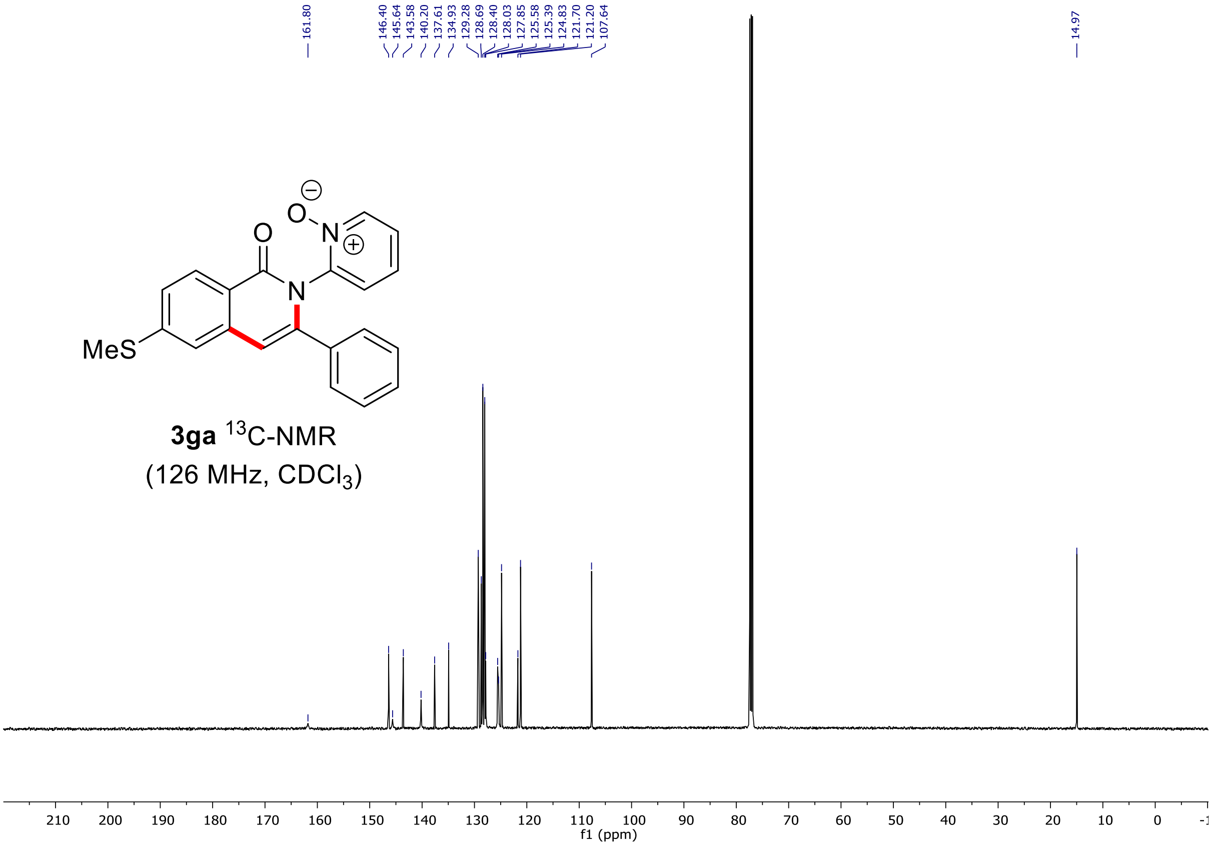

3ga ^{13}C-NMR
(126 MHz, $CDCl_3$)

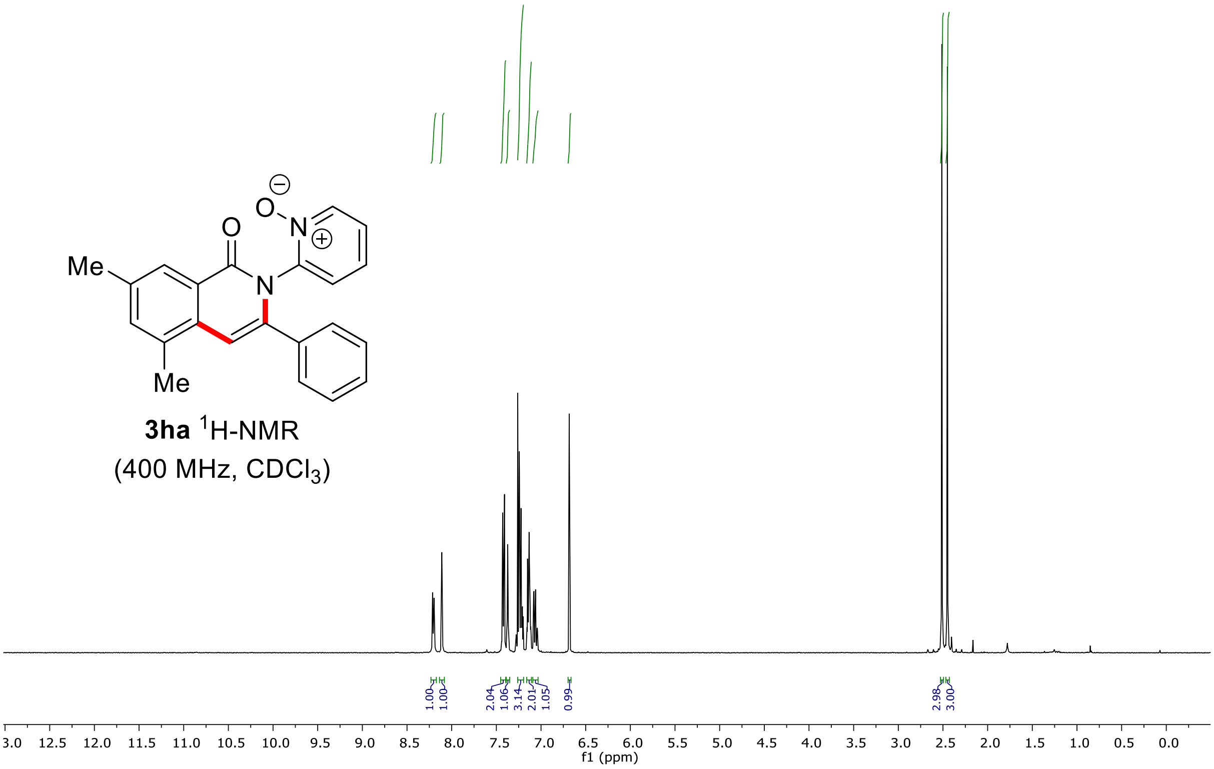

3ha ^{1}H-NMR
(400 MHz, $CDCl_3$)

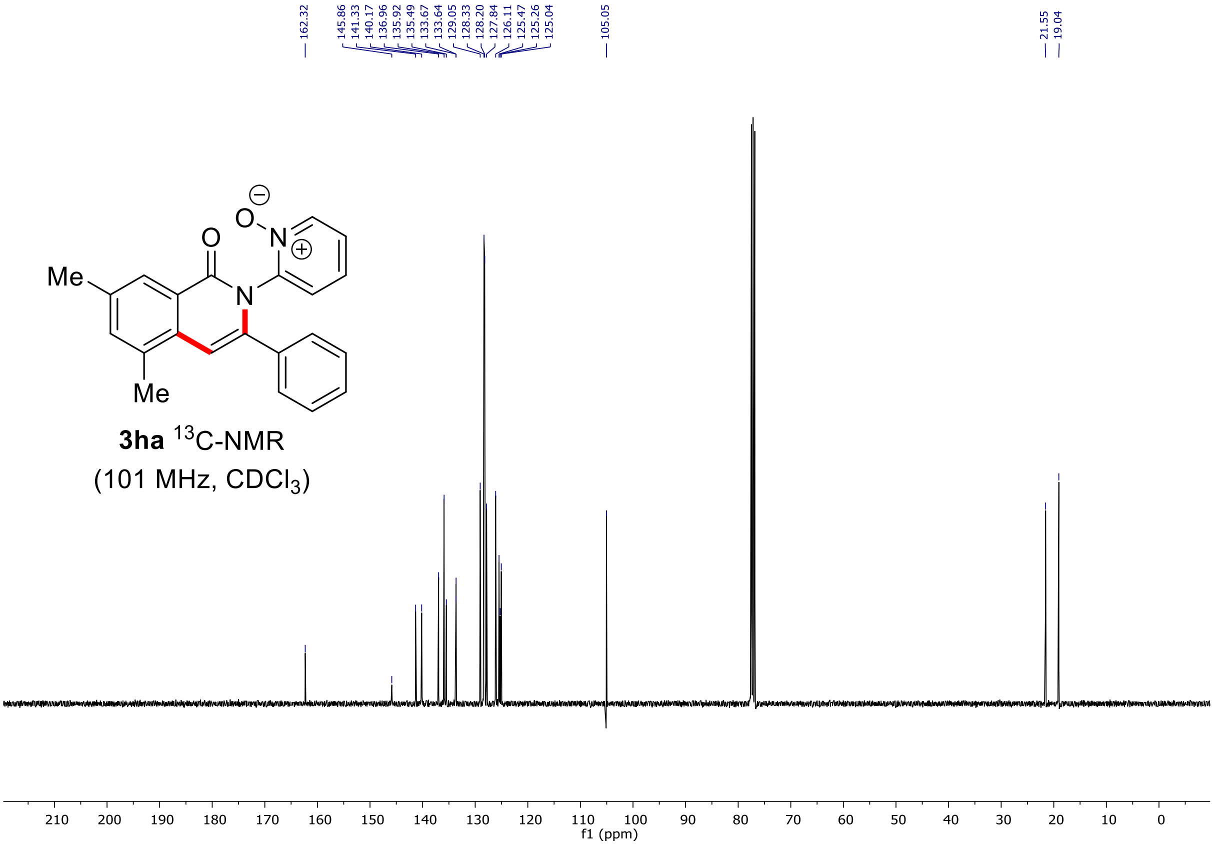

3ha ^{13}C-NMR
(101 MHz, $CDCl_3$)

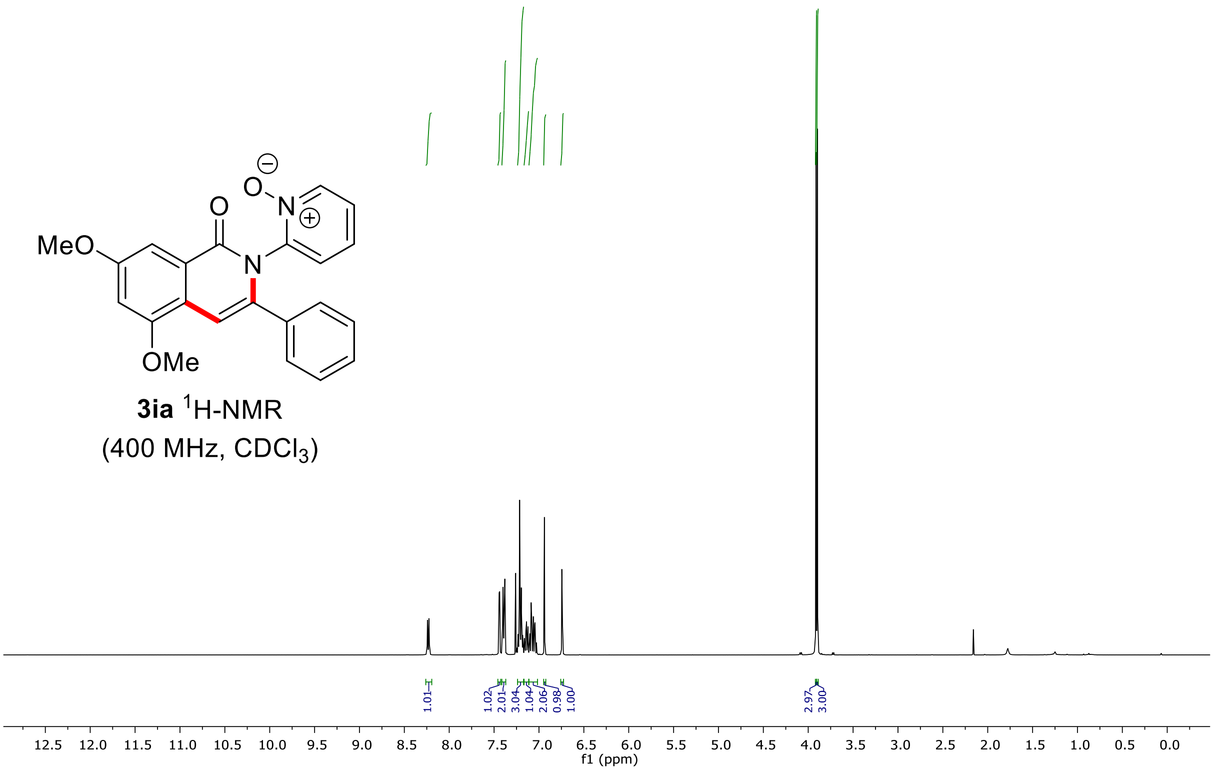

3ia ^{1}H-NMR
(400 MHz, $CDCl_3$)

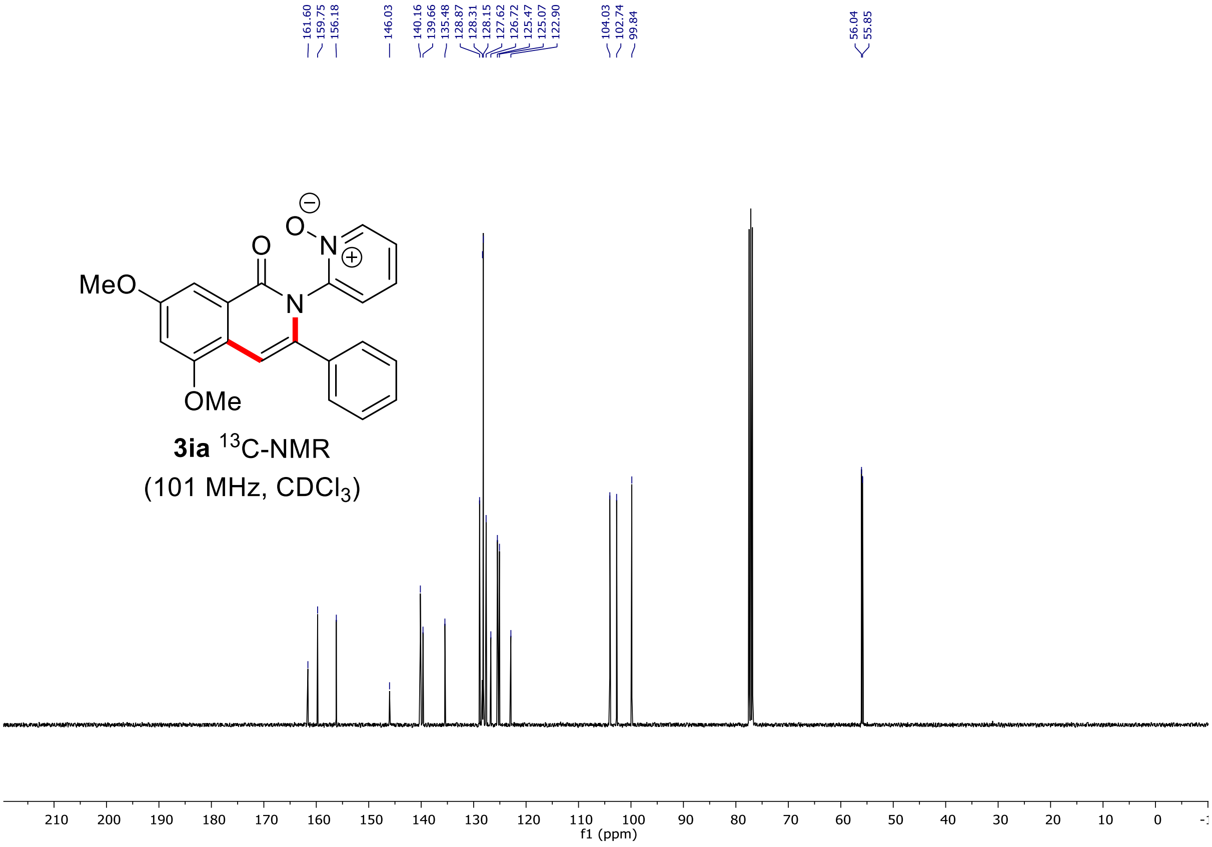

3ia ^{13}C-NMR
(101 MHz, $CDCl_3$)

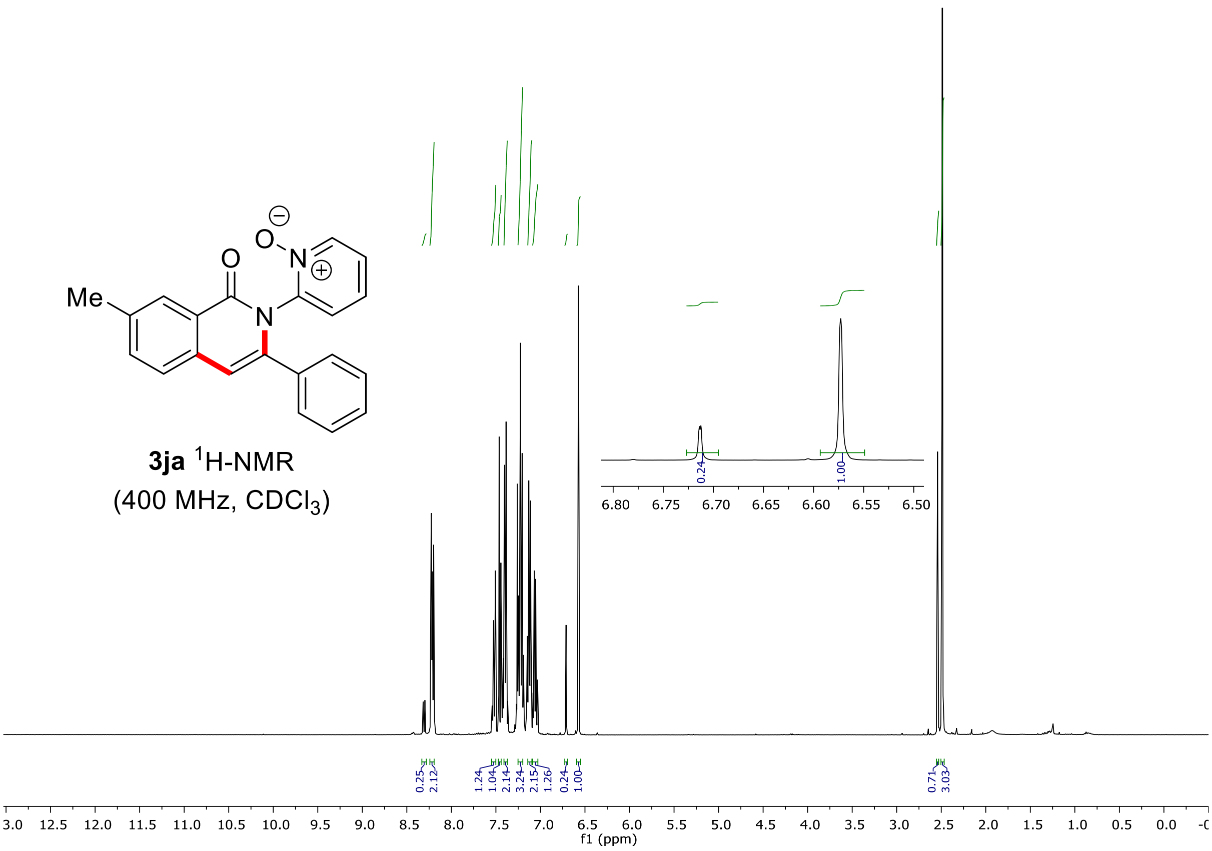

3ja ^{1}H-NMR
(400 MHz, $CDCl_3$)

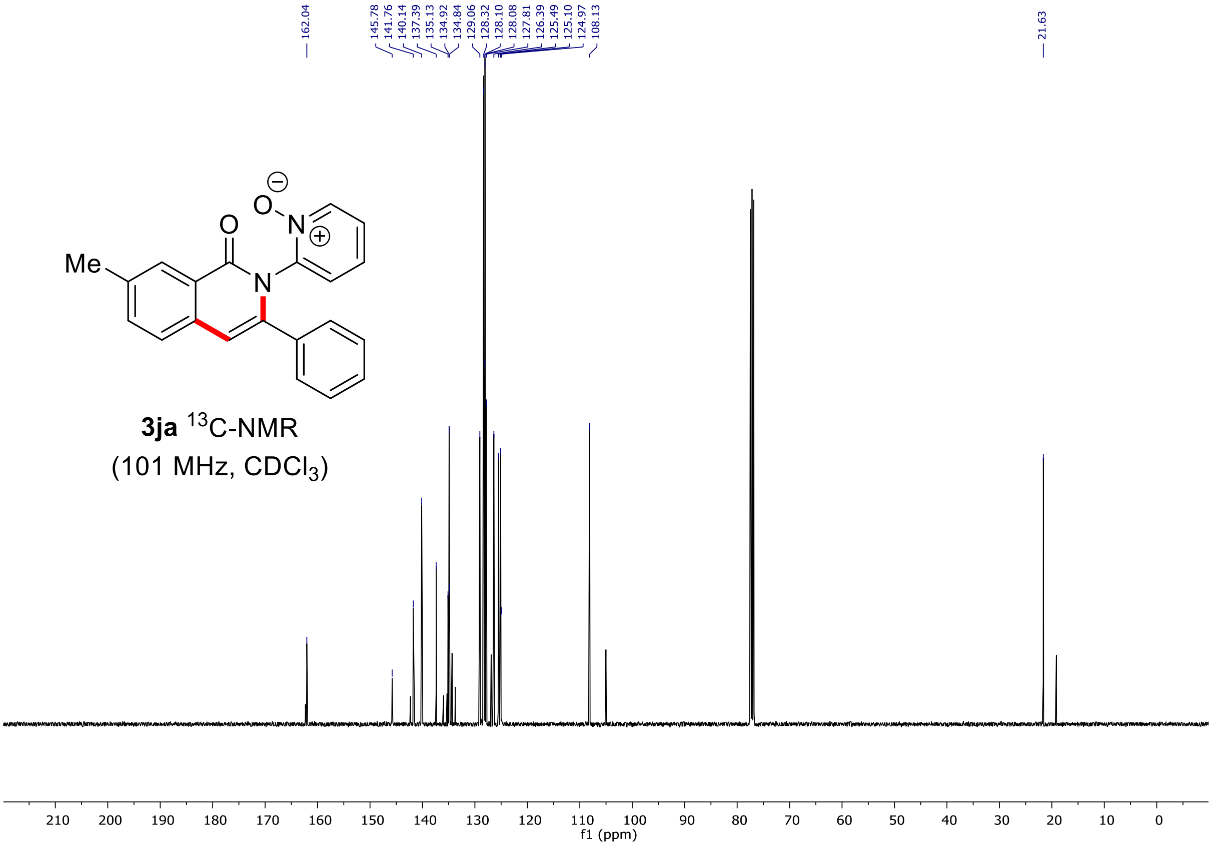
162.04
145.78
141.76
140.14
137.39
135.13
134.92
134.84
129.06
128.32
128.10
128.08
127.81
126.39
125.49
125.10
124.97
108.13
21.63
Me
O
N
3ja ^{13}C-NMR
(101 MHz, $CDCl_3$)
f1 (ppm)

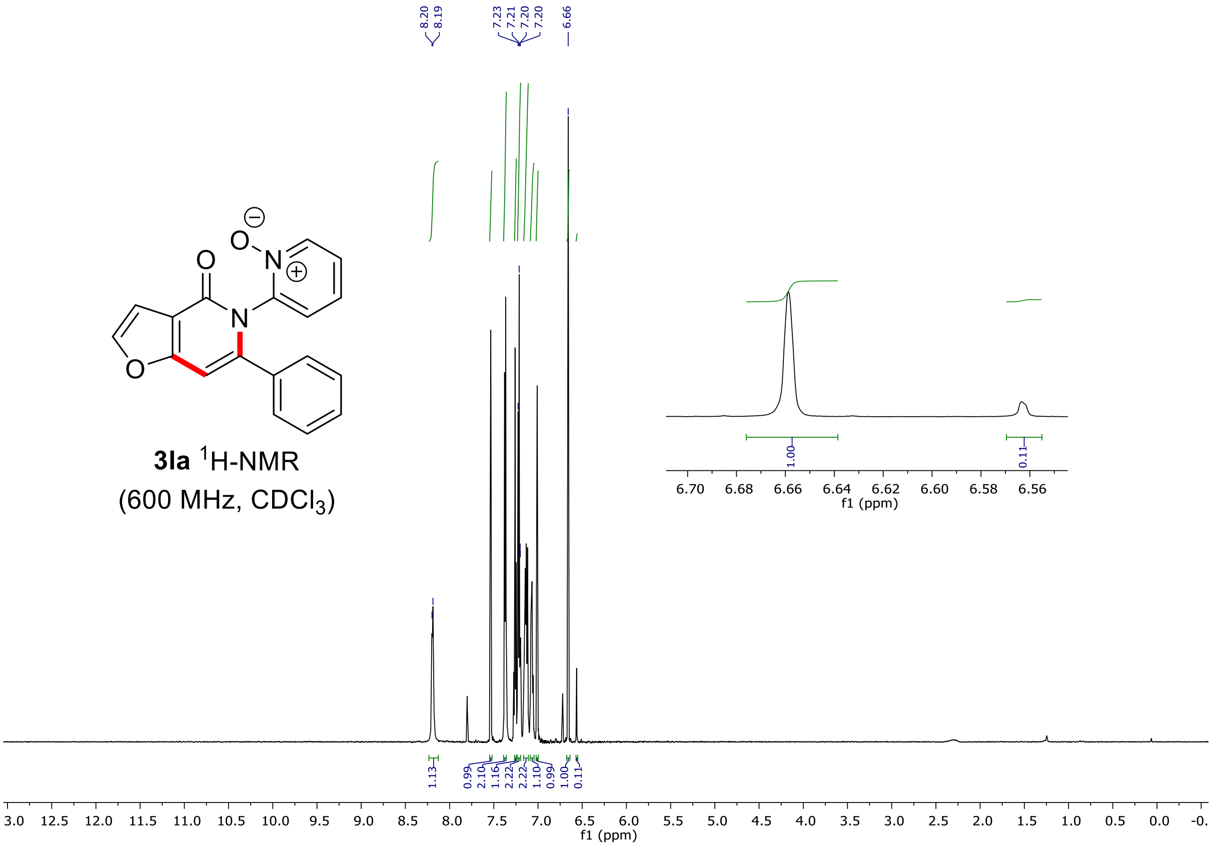
8.20
8.19
7.23
7.21
7.20
7.20
6.66
O
N
3la ^{1}H-NMR
(600 MHz, $CDCl_3$)
1.00
0.11
f1 (ppm)
1.13
0.99
2.10
1.16
2.22
2.22
1.10
0.99
1.00
0.11
f1 (ppm)

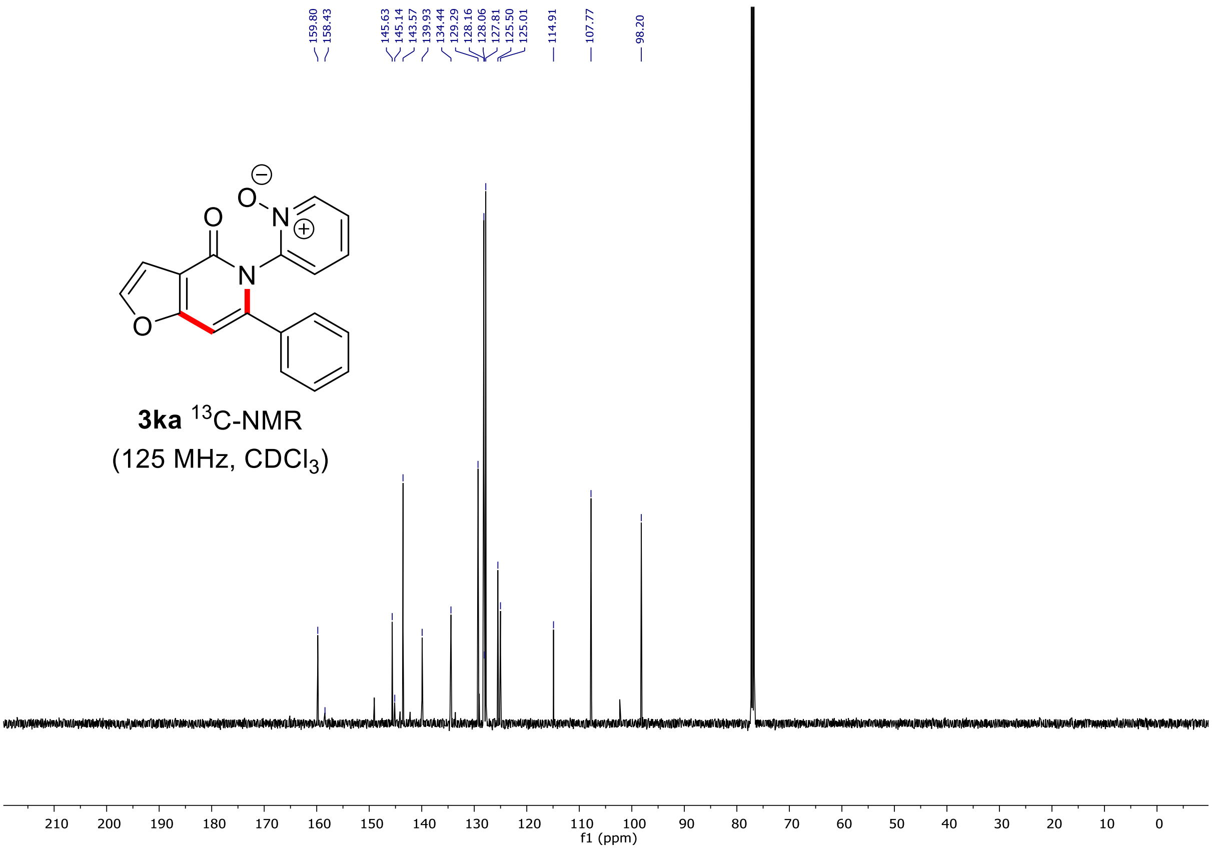

3ka ^{13}C-NMR
(125 MHz, CDCl$_3$)
159.80
158.43
145.63
145.14
143.57
139.93
134.44
129.29
128.16
128.06
127.81
125.50
125.01
114.91
107.77
98.20
f1 (ppm)

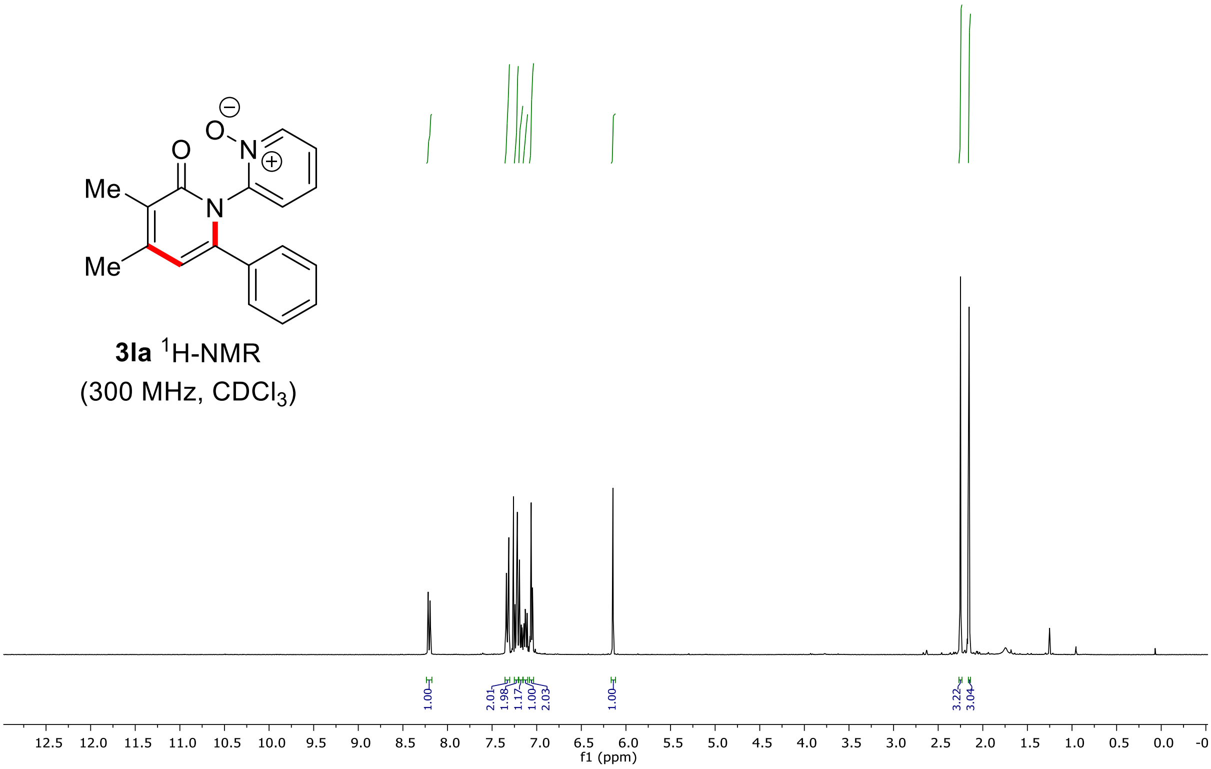

3la ^{1}H-NMR
(300 MHz, CDCl$_3$)
1.00
2.01
1.98
1.17
1.00
2.03
1.00
3.22
3.04
f1 (ppm)

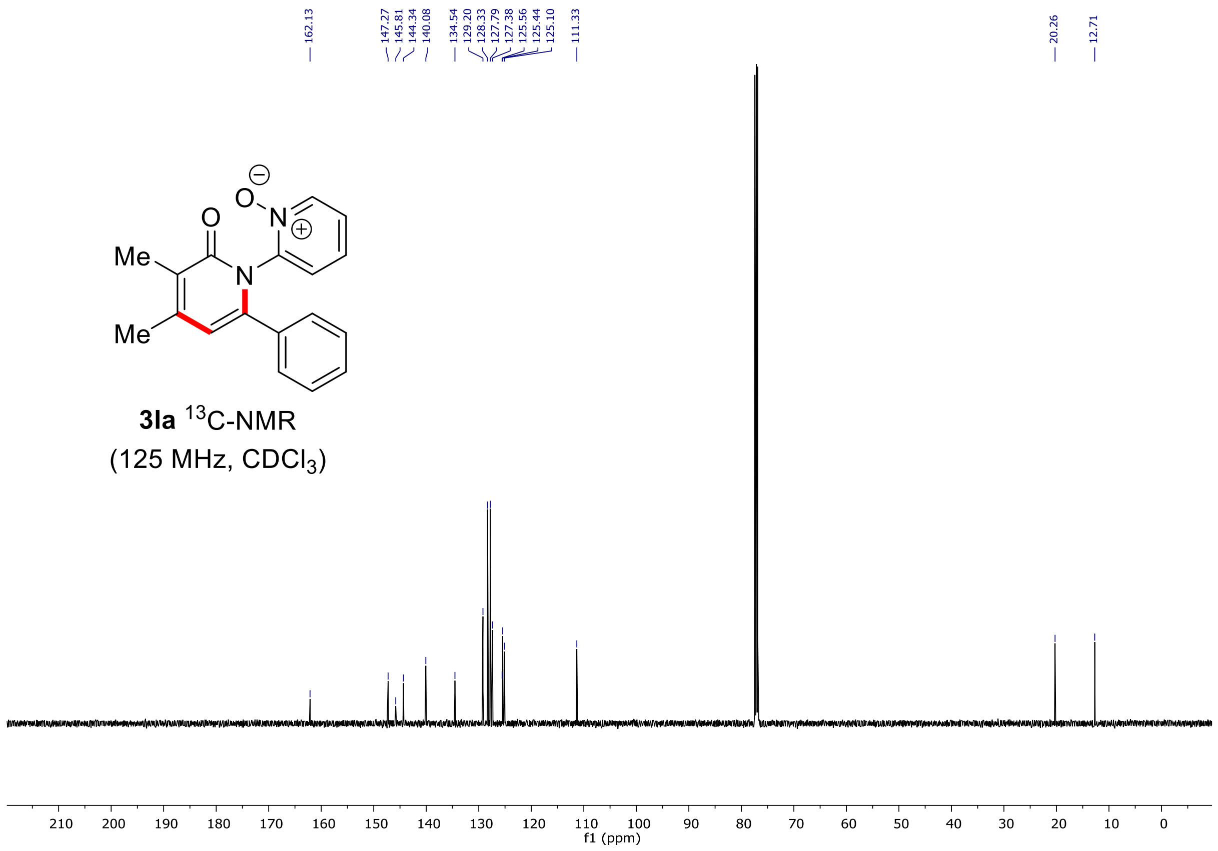

162.13
147.27
145.81
144.34
140.08
134.54
129.20
128.33
127.79
127.38
125.56
125.44
125.10
111.33
20.26
12.71
Me
Me
O
N
3la ^{13}C-NMR
(125 MHz, CDCl$_3$)
f1 (ppm)

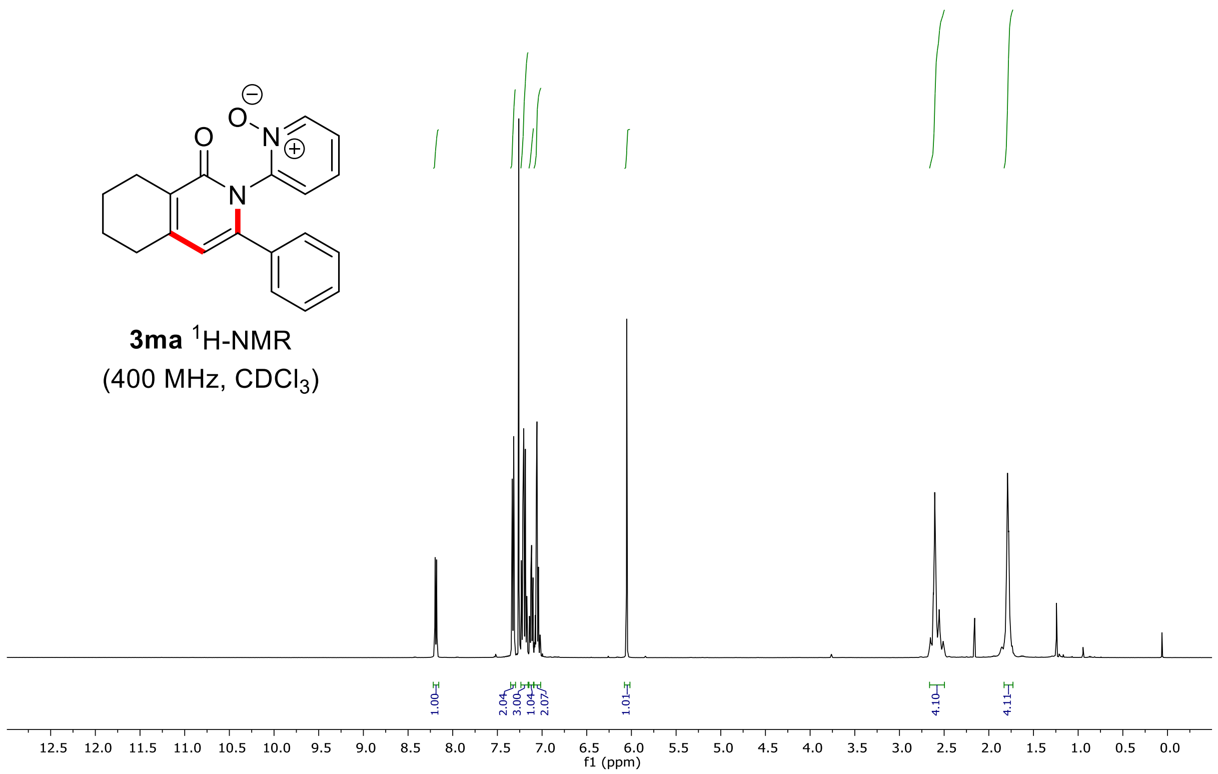

O
N
3ma ^{1}H-NMR
(400 MHz, CDCl$_3$)
1.00
2.04
3.00
1.04
2.07
1.01
4.10
4.11
f1 (ppm)

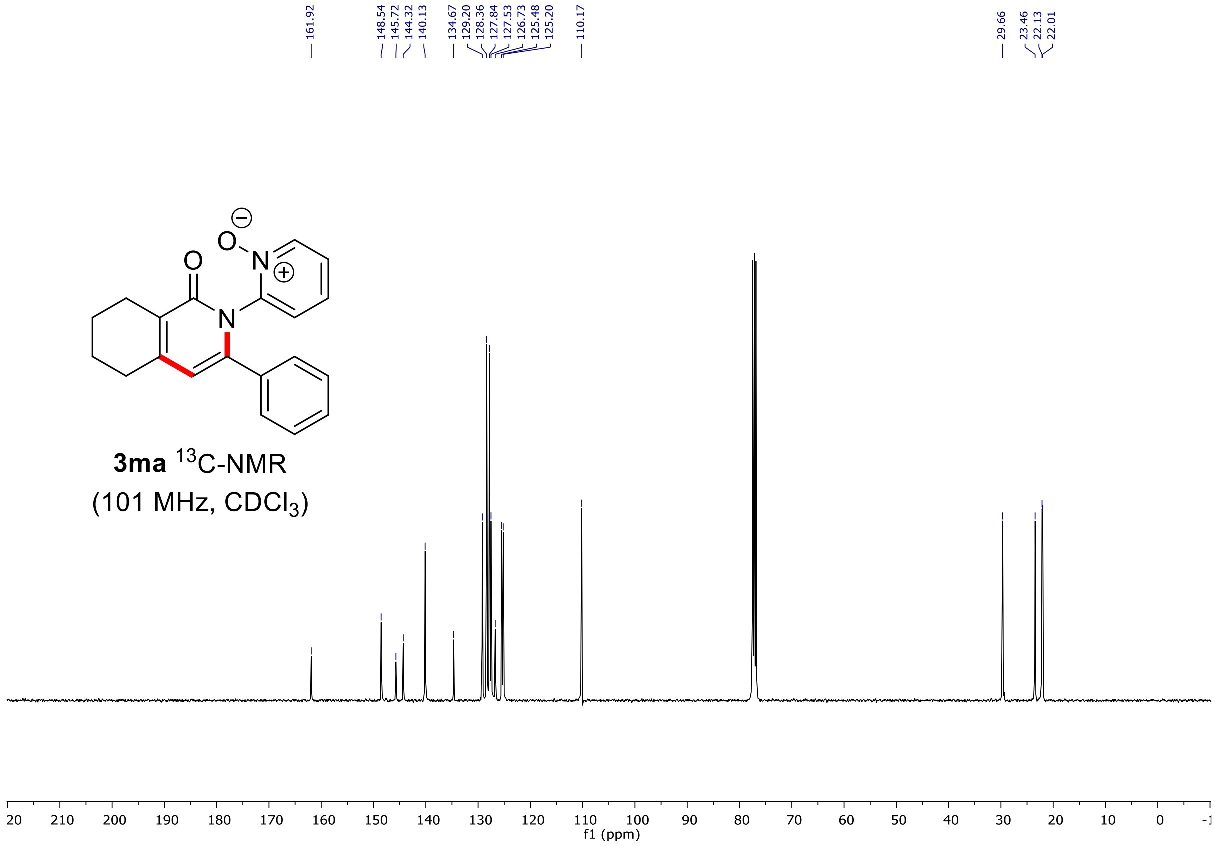

161.92
148.54
145.72
144.32
140.13
134.67
129.20
128.36
127.84
127.53
126.73
125.48
125.20
110.17
29.66
23.46
22.13
22.01
3ma ^{13}C-NMR
(101 MHz, $CDCl_3$)
f1 (ppm)

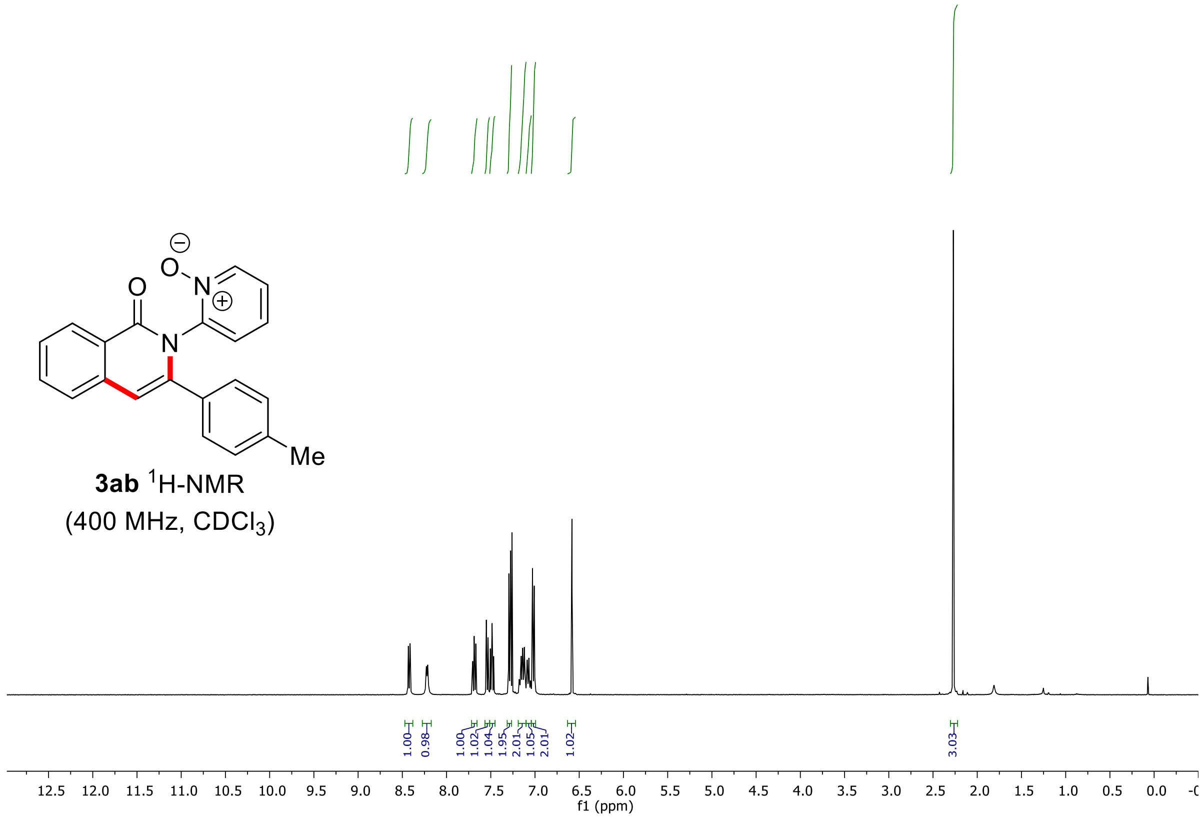

Me
3ab ^{1}H-NMR
(400 MHz, $CDCl_3$)
1.00
0.98
1.00
1.02
1.04
1.95
2.01
1.05
2.01
1.02
3.03
f1 (ppm)

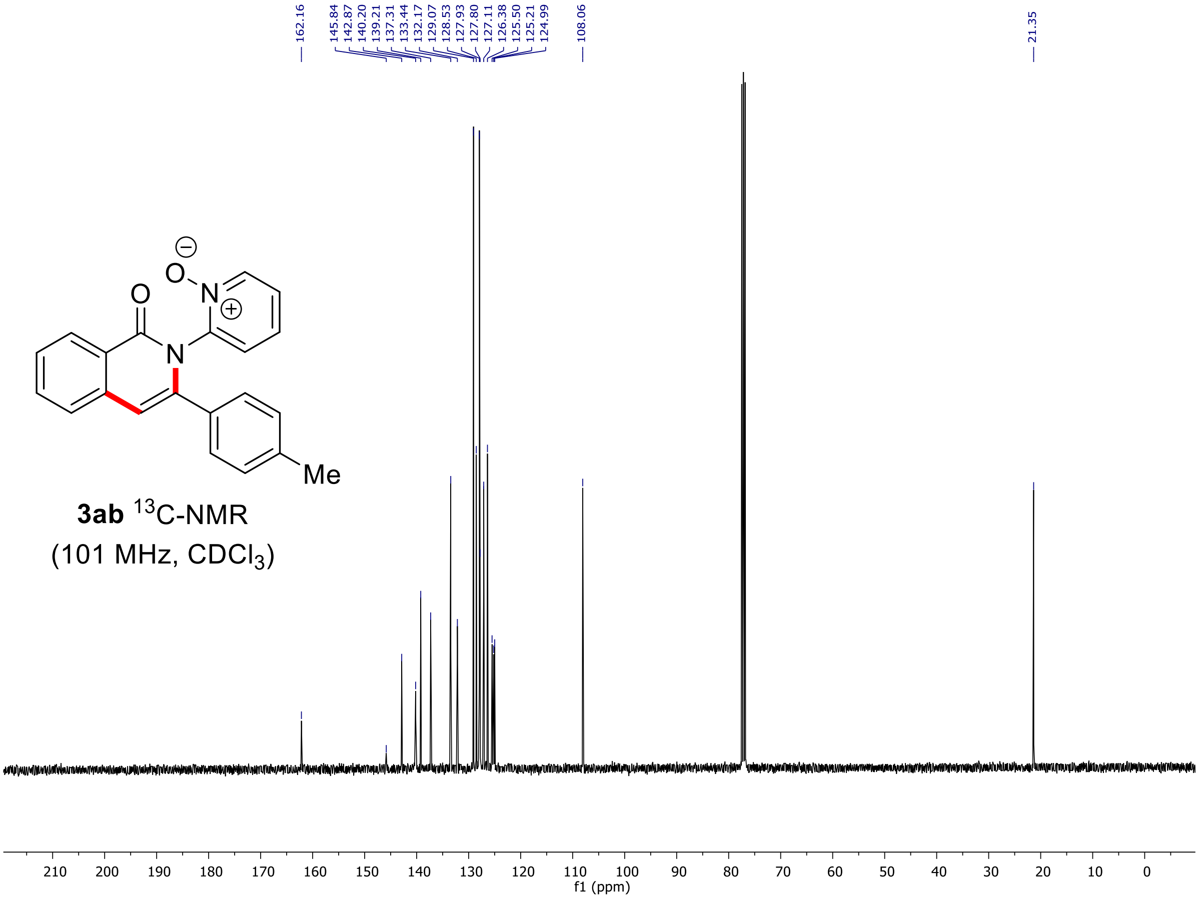
162.16
145.84
142.87
140.20
139.21
137.31
133.44
132.17
129.07
128.53
127.93
127.80
127.11
126.38
125.50
125.21
124.99
108.06
21.35
Me
3ab ^{13}C-NMR
(101 MHz, $CDCl_3$)
f1 (ppm)

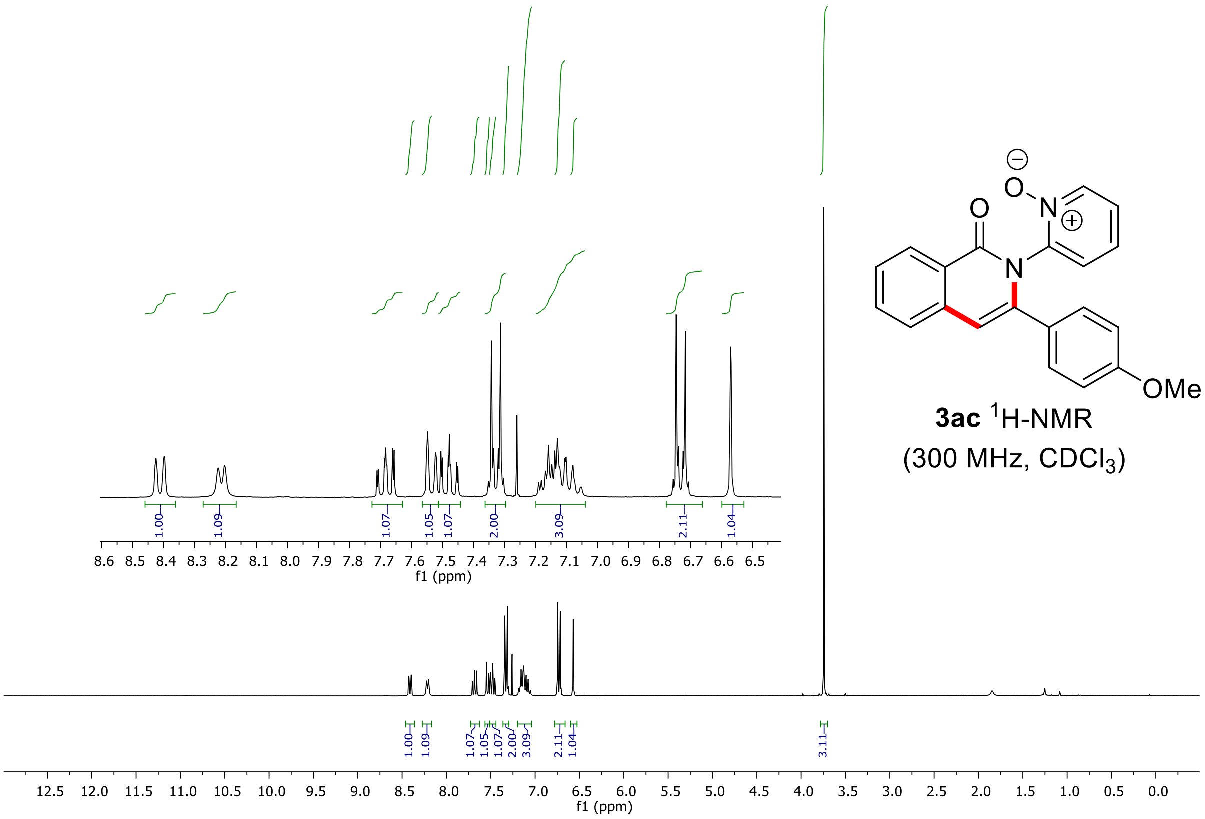
OMe
3ac ^{1}H-NMR
(300 MHz, $CDCl_3$)
1.00
1.09
1.07
1.05
1.07
2.00
3.09
2.11
1.04
3.11
f1 (ppm)

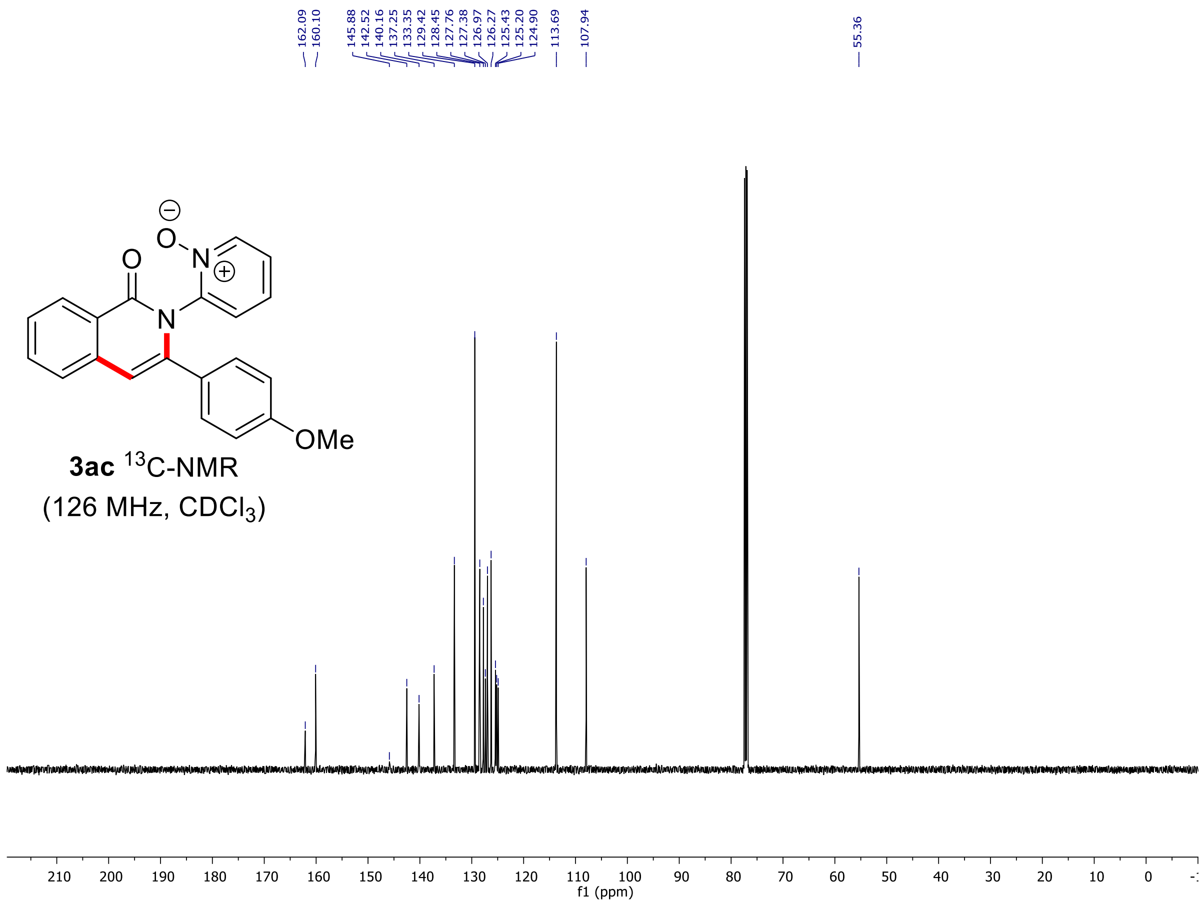
162.09
160.10
145.88
142.52
140.16
137.25
133.35
129.42
128.45
127.76
127.38
126.97
126.27
125.43
125.20
124.90
113.69
107.94
55.36
3ac ^{13}C-NMR
(126 MHz, CDCl$_3$)
f1 (ppm)

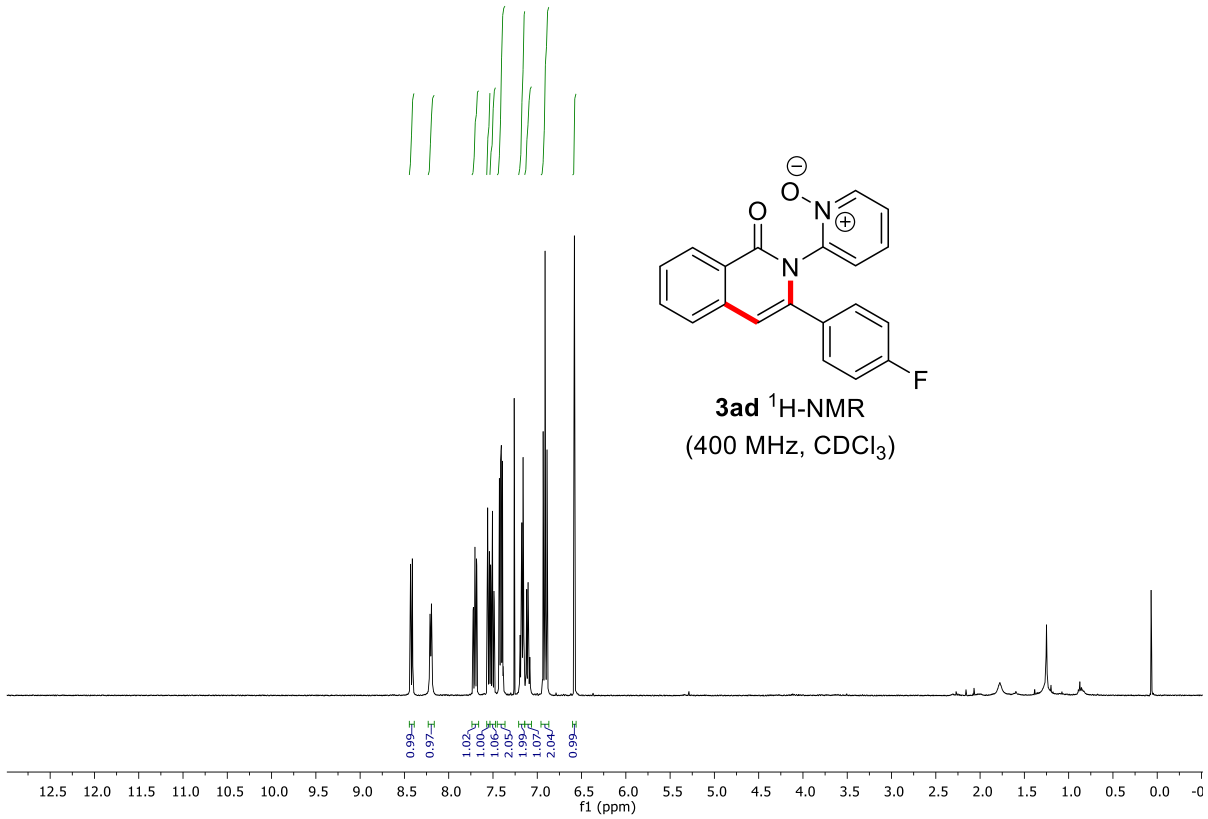
3ad ^{1}H-NMR
(400 MHz, CDCl$_3$)
0.99
0.97
1.02
1.00
1.06
2.05
1.99
1.07
2.04
0.99
f1 (ppm)

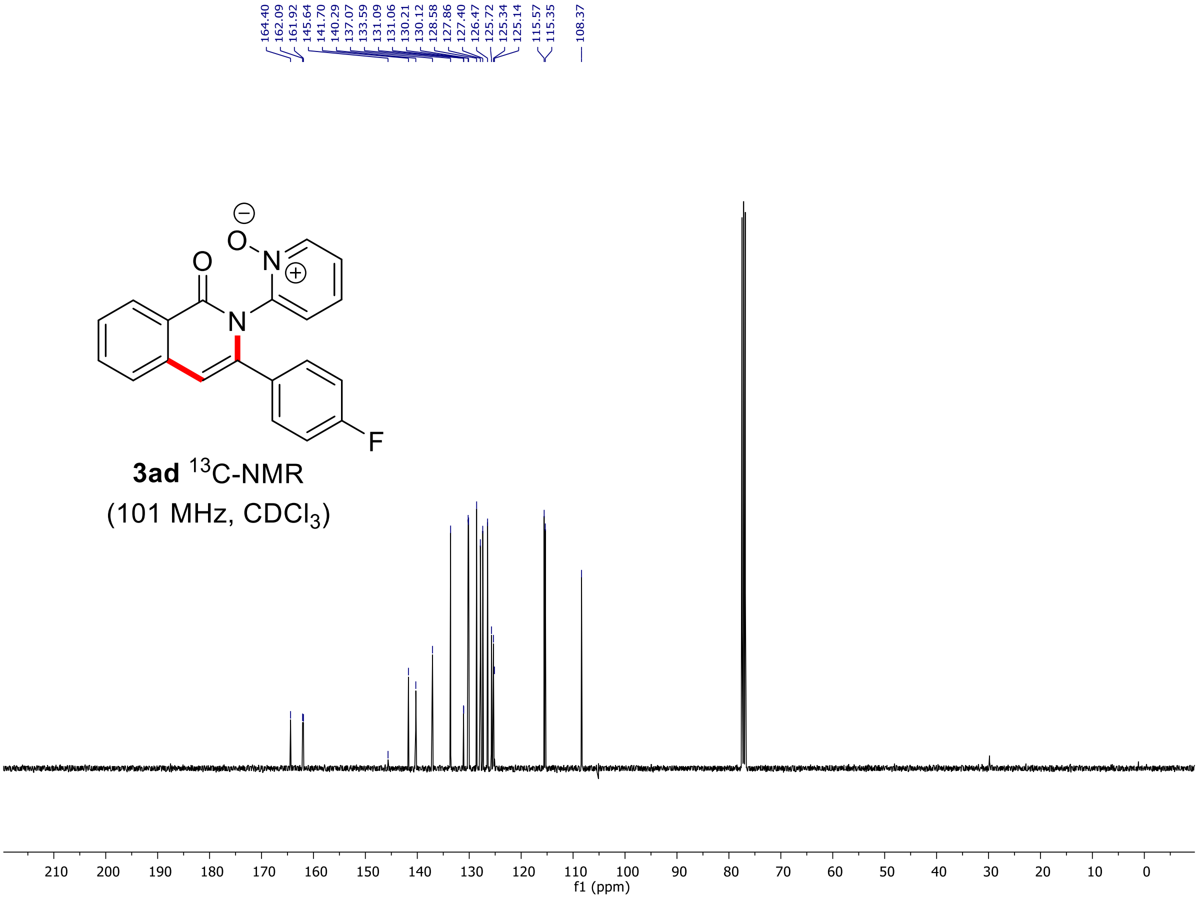

3ad ^{13}C-NMR
(101 MHz, $CDCl_3$)

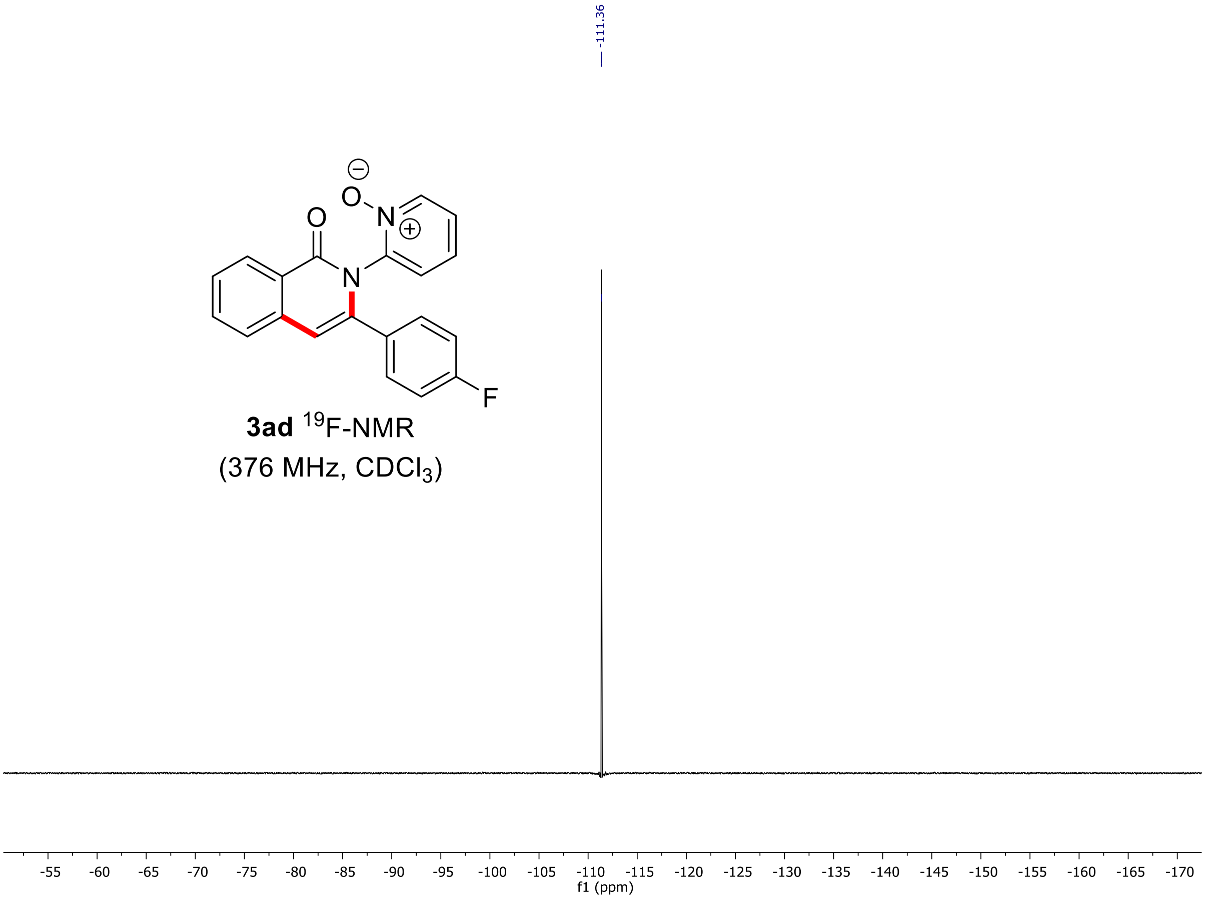

3ad ^{19}F-NMR
(376 MHz, $CDCl_3$)

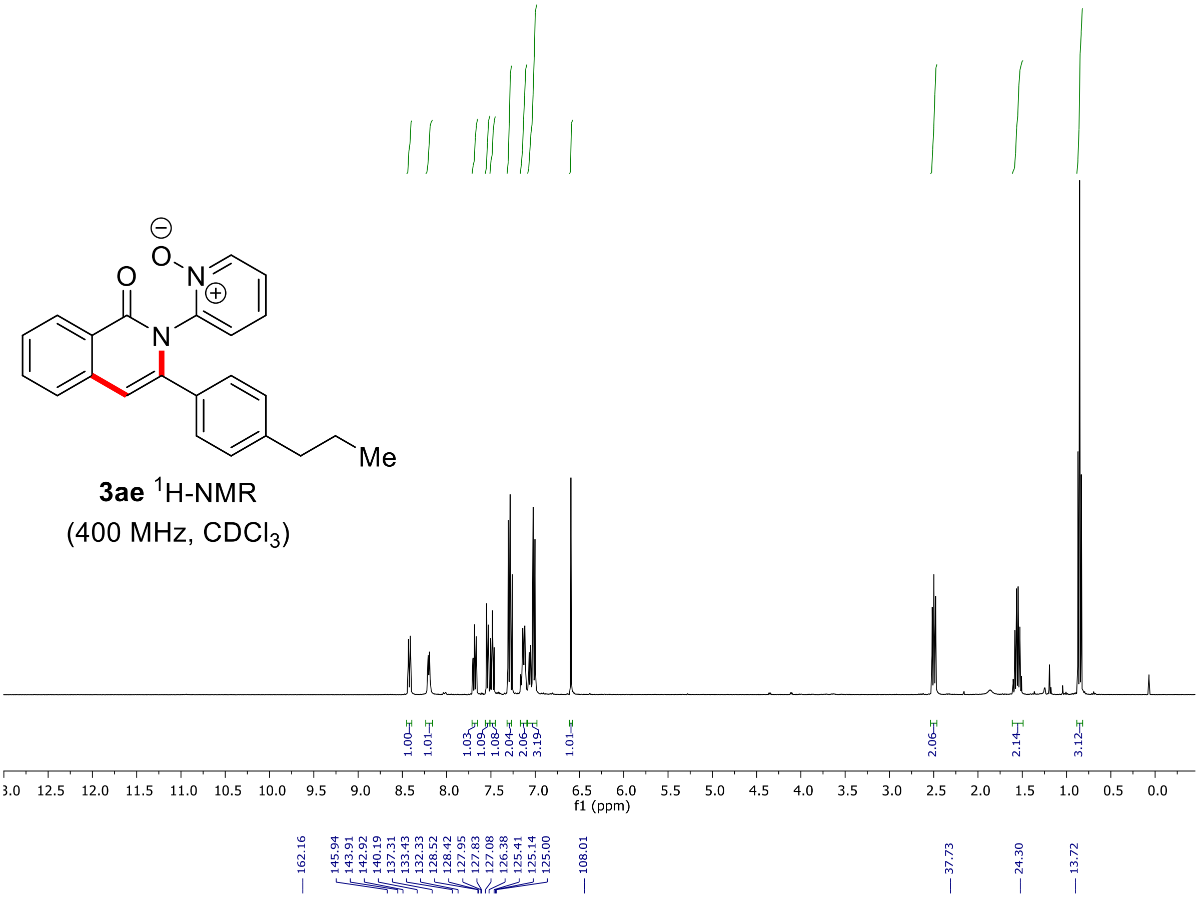
3ae ¹H-NMR
(400 MHz, CDCl3)
1.00
1.01
1.03
1.09
1.08
2.04
2.06
3.19
1.01
2.06
2.14
3.12
f1 (ppm)
162.16
145.94
143.91
142.92
140.19
137.31
133.43
132.33
128.52
128.42
127.95
127.83
127.08
126.38
125.41
125.14
125.00
108.01
37.73
24.30
13.72

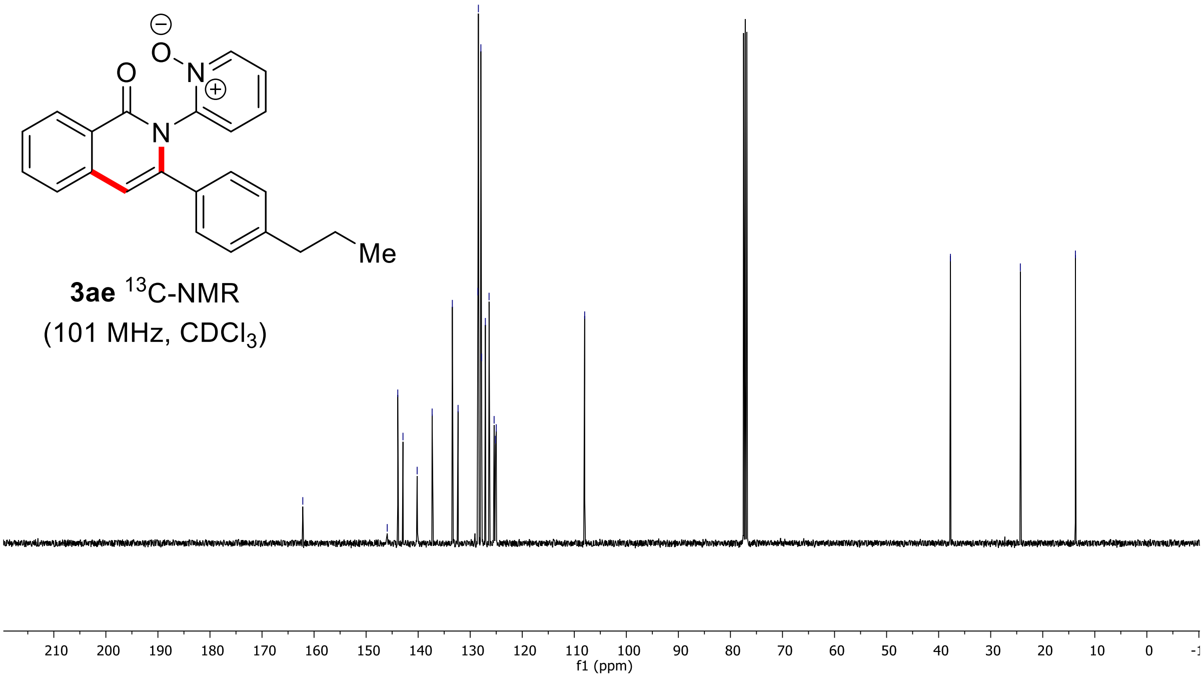
3ae ¹³C-NMR
(101 MHz, CDCl3)
f1 (ppm)

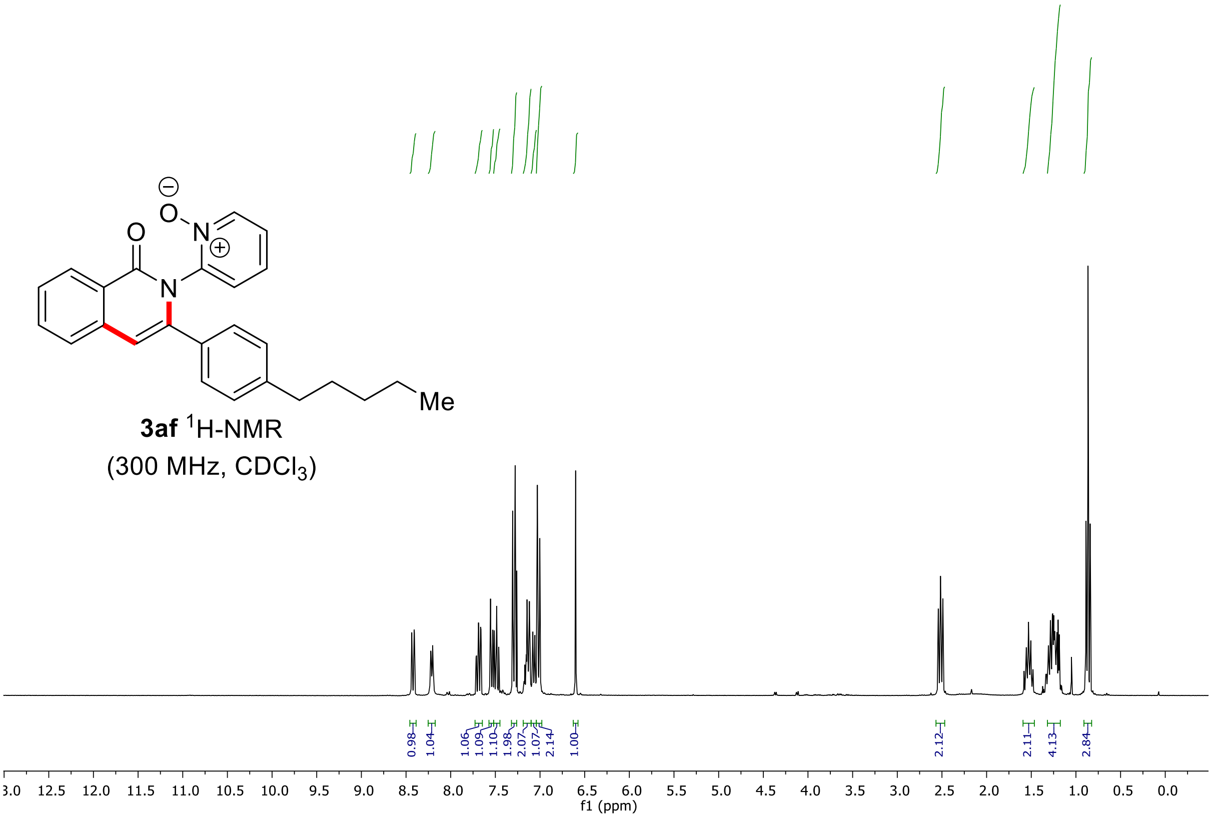

3af ^{1}H-NMR
(300 MHz, $CDCl_3$)

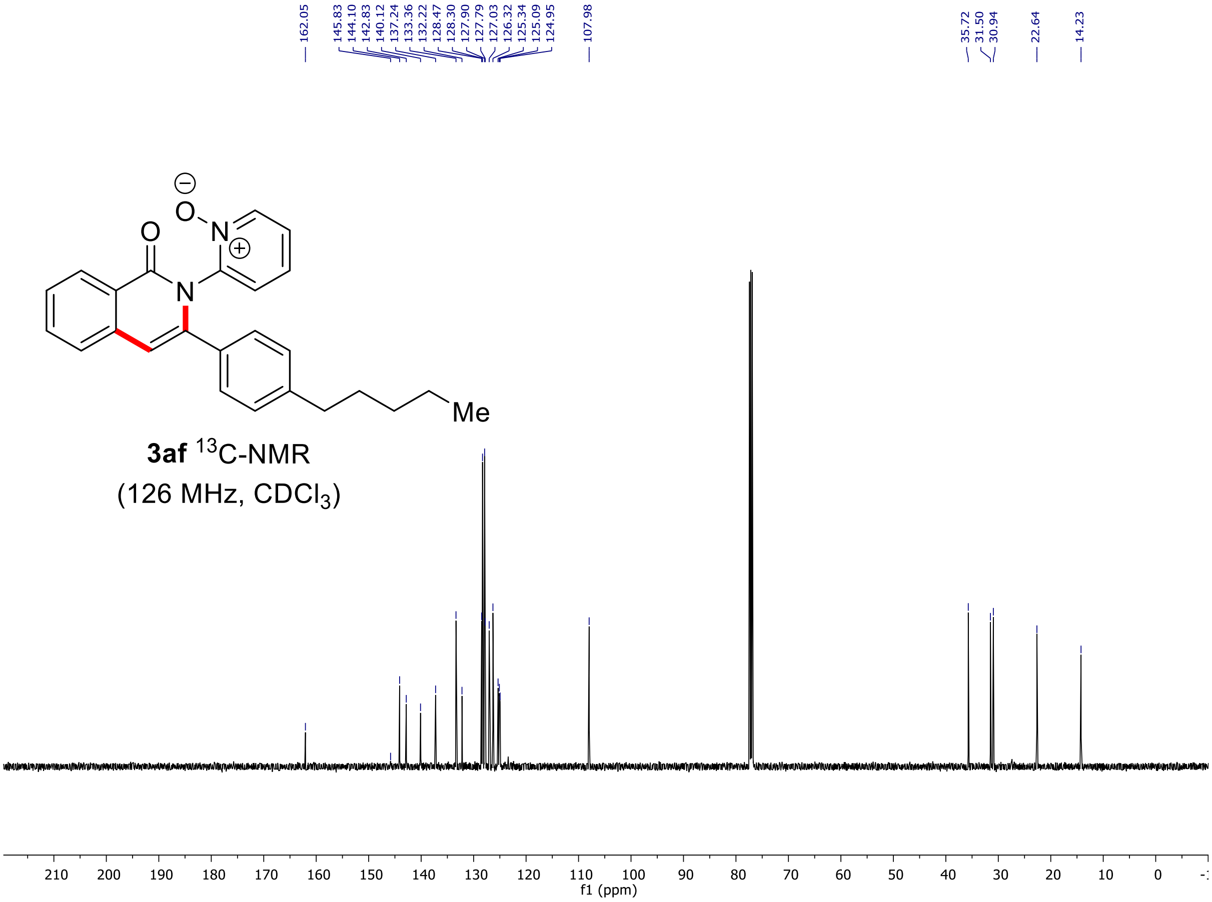

3af ^{13}C-NMR
(126 MHz, $CDCl_3$)

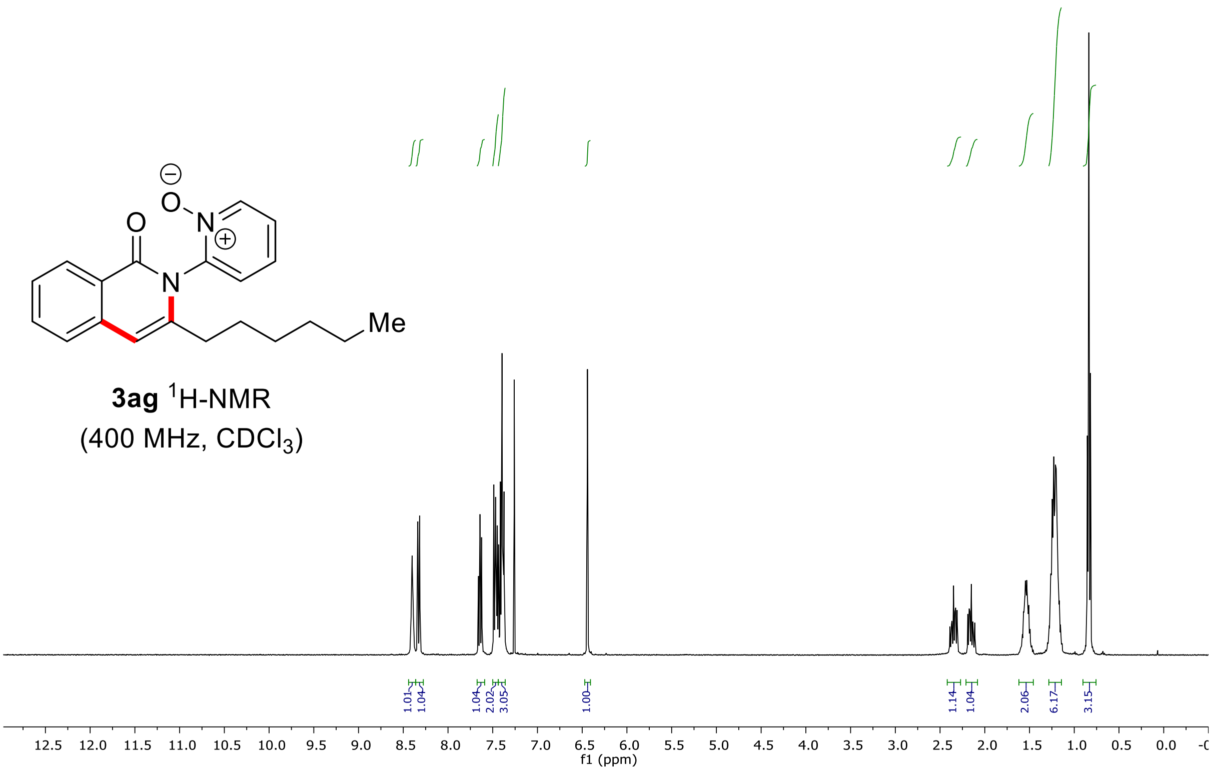
3ag ^{1}H-NMR
(400 MHz, $CDCl_3$)
O
N
Me
1.01
1.04
1.04
2.02
3.05
1.00
1.14
1.04
2.06
6.17
3.15
f1 (ppm)

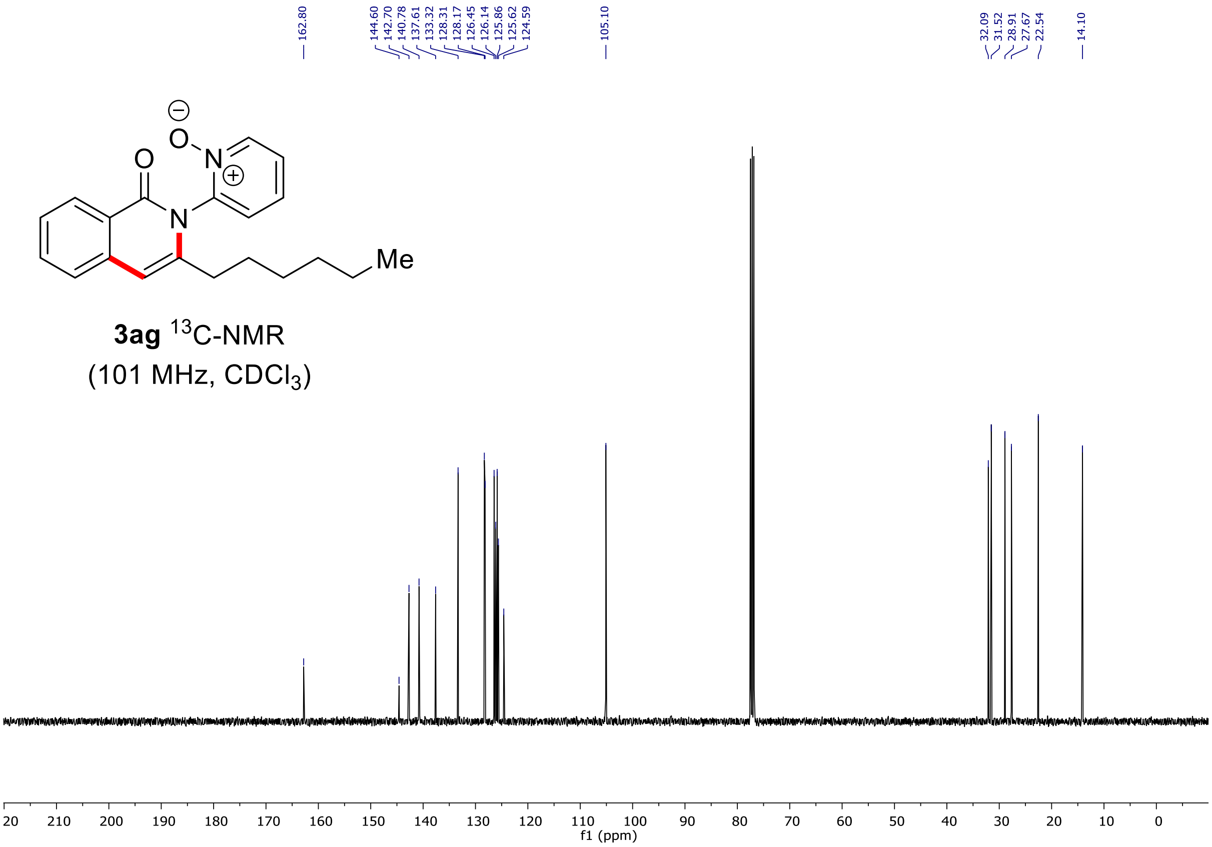
162.80
144.60
142.70
140.78
137.61
133.32
128.31
128.17
126.45
126.14
125.86
125.62
124.59
105.10
32.09
31.52
28.91
27.67
22.54
14.10
3ag ^{13}C-NMR
(101 MHz, $CDCl_3$)
O
N
Me
f1 (ppm)

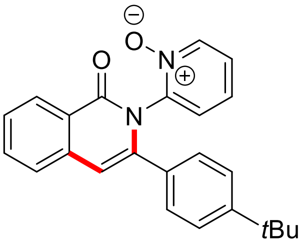

3ah ^{1}H-NMR
(400 MHz, $CDCl_3$)

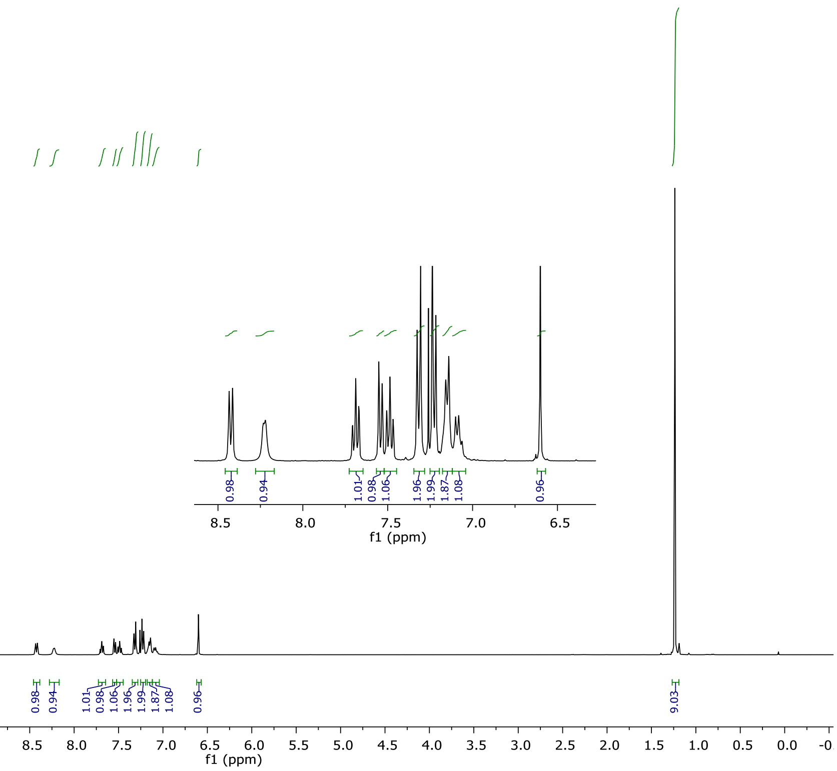

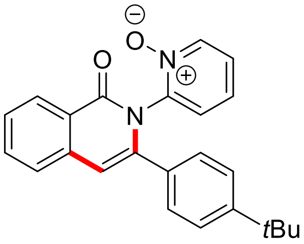

3ah ^{13}C-NMR
(126 MHz, $CDCl_3$)

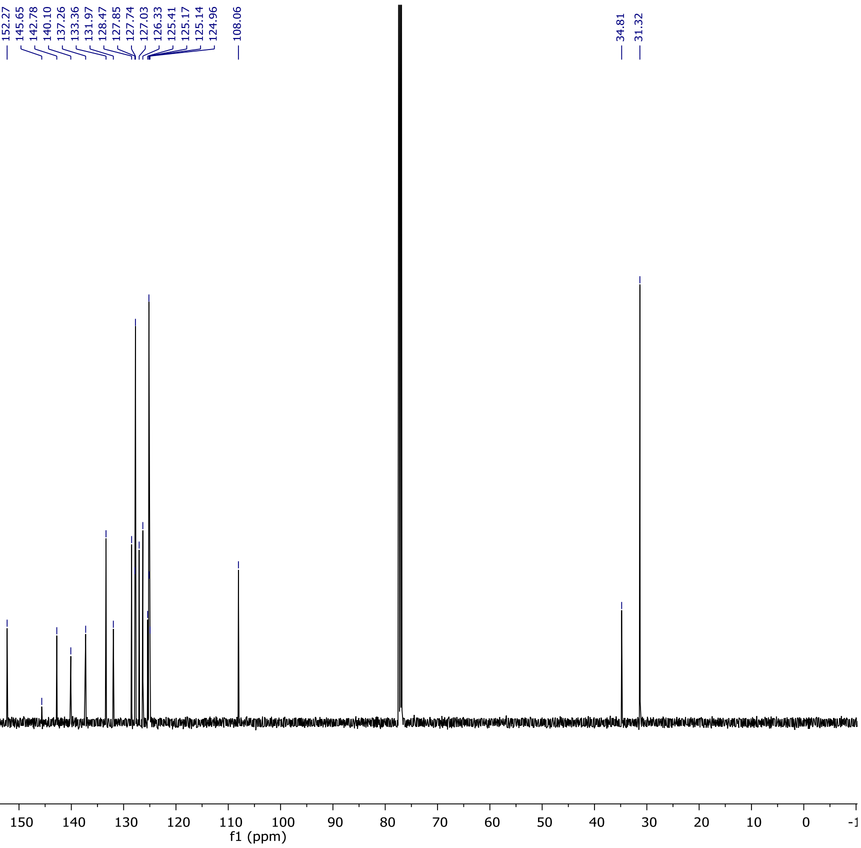

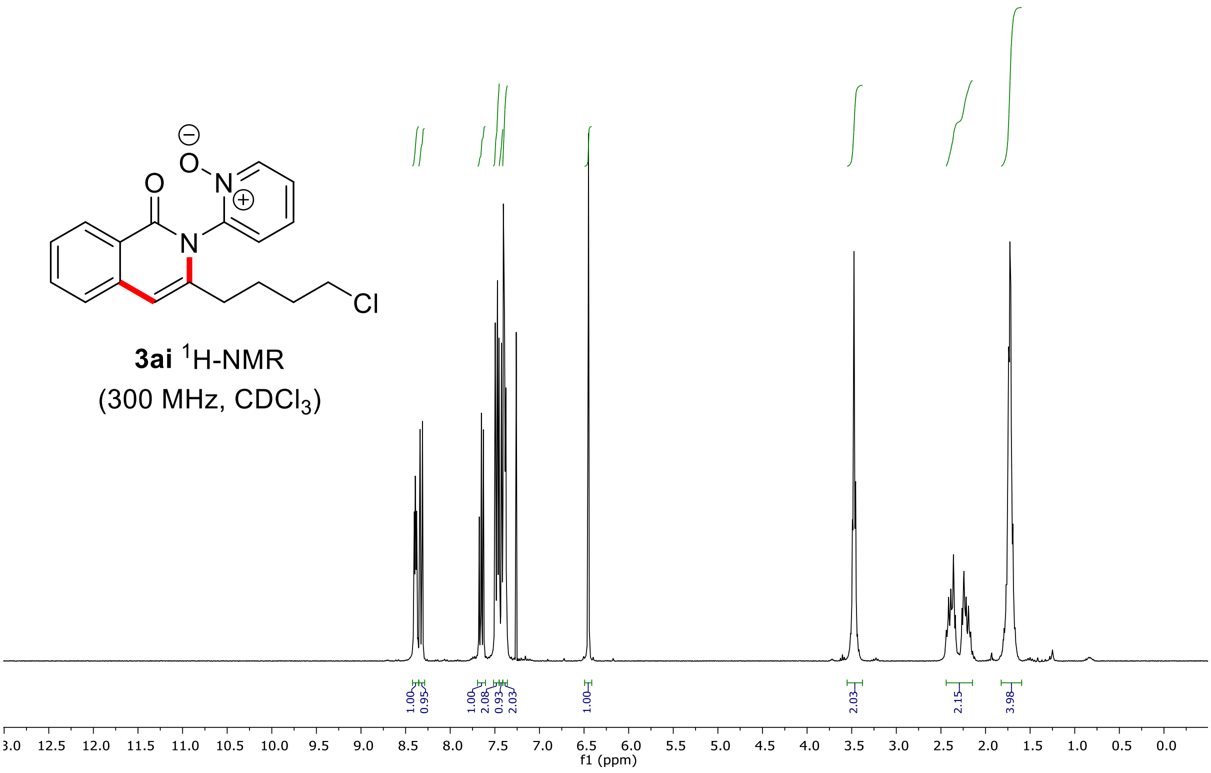

3ai ^{1}H-NMR
(300 MHz, $CDCl_3$)

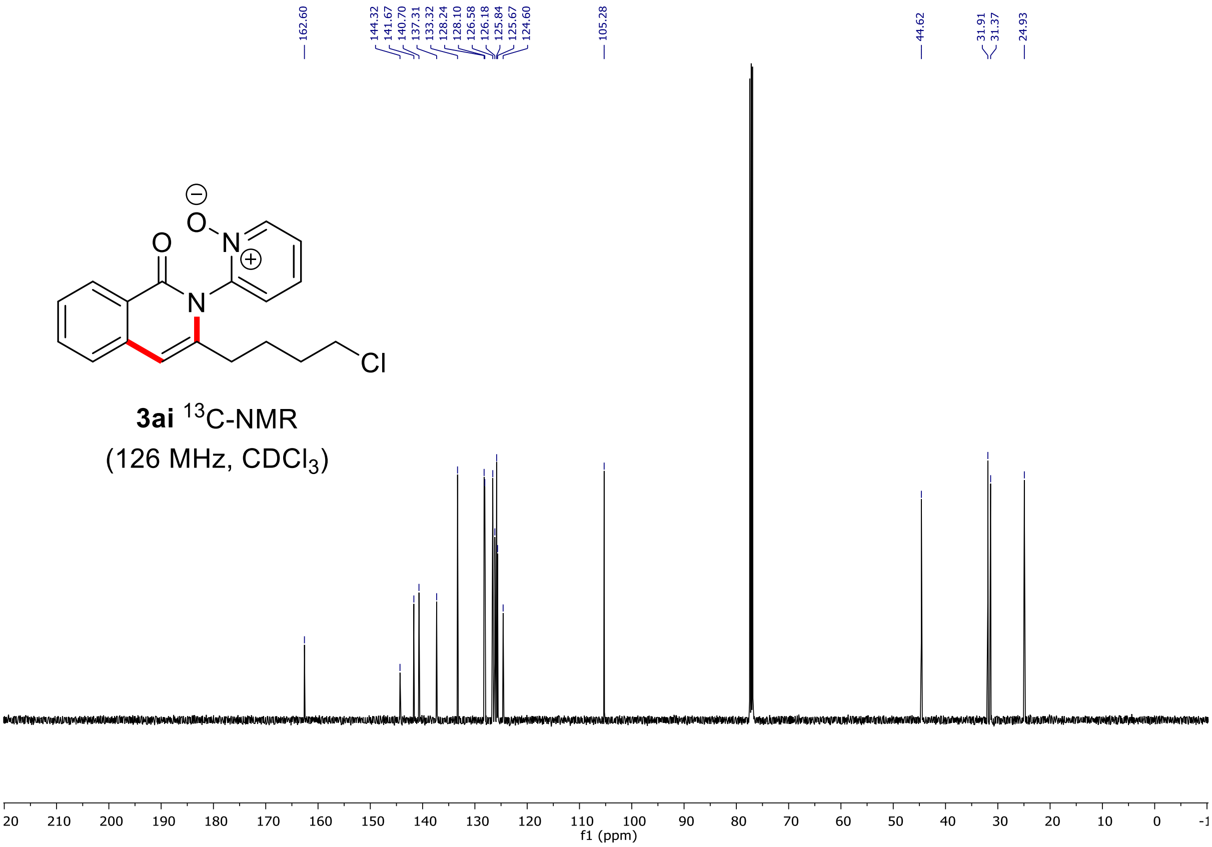

3ai ^{13}C-NMR
(126 MHz, $CDCl_3$)

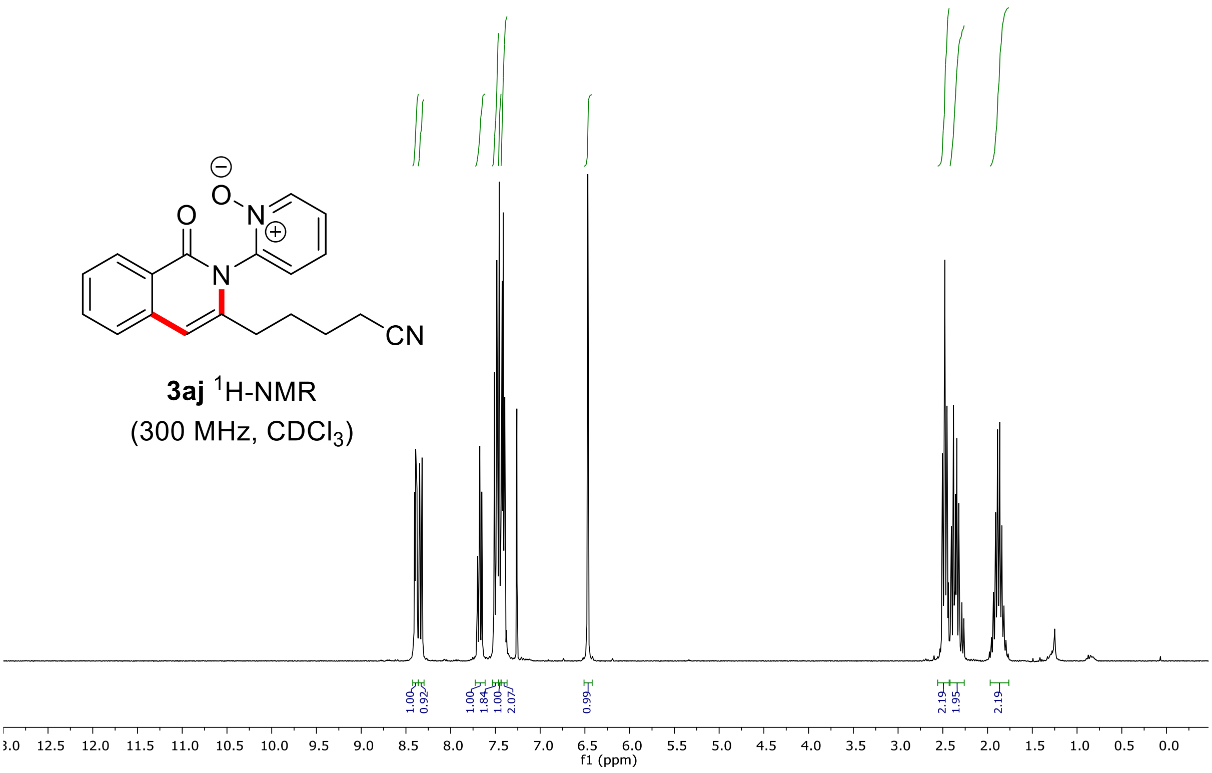
3aj ^{1}H-NMR
(300 MHz, $CDCl_3$)
CN
1.00
0.92
1.00
1.84
1.00
2.07
0.99
2.19
1.95
2.19
f1 (ppm)

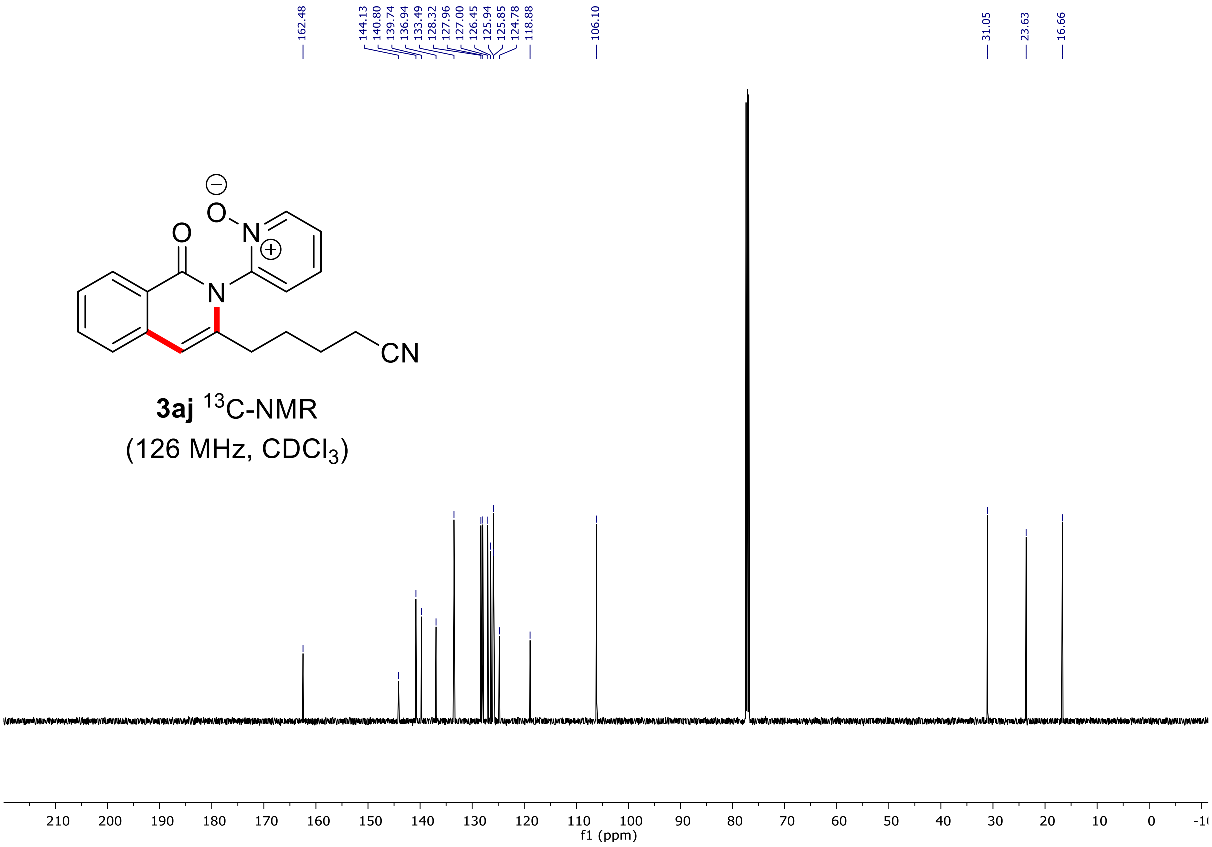
162.48
144.13
140.80
139.74
136.94
133.49
128.32
127.96
127.00
126.45
125.94
125.85
124.78
118.88
106.10
31.05
23.63
16.66
3aj ^{13}C-NMR
(126 MHz, $CDCl_3$)
CN
f1 (ppm)

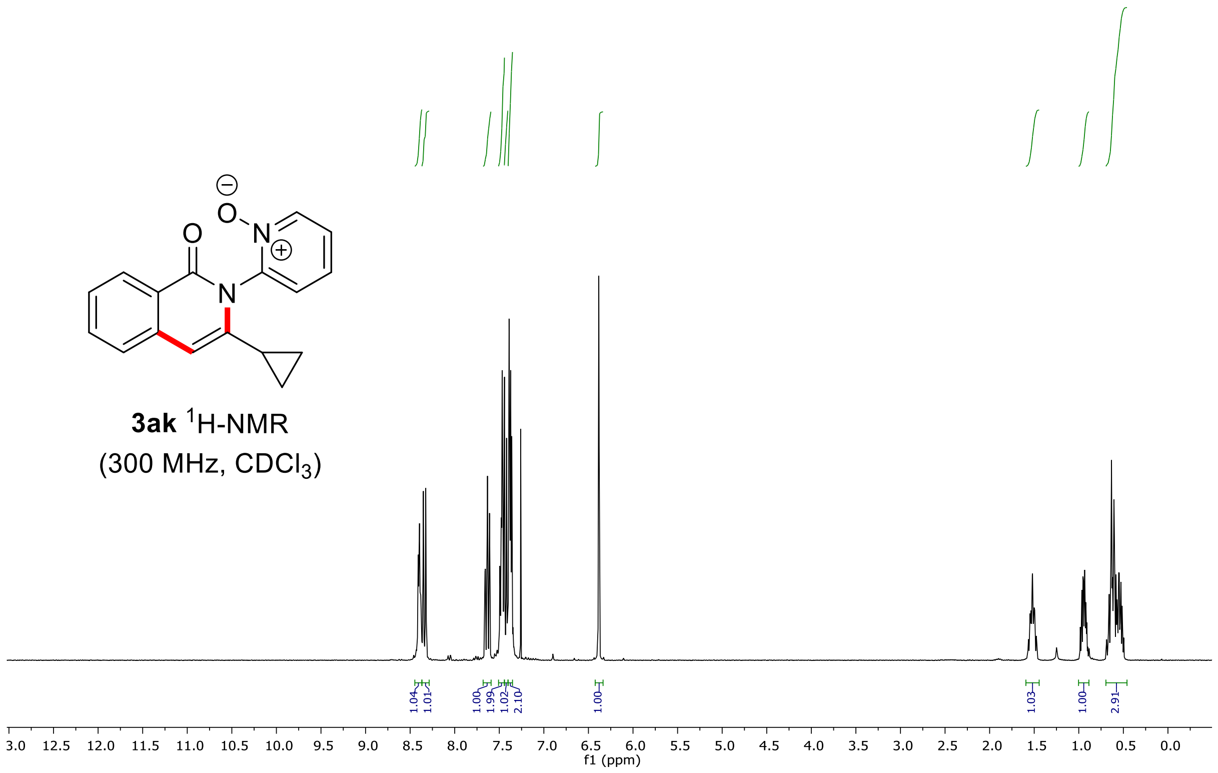

3ak ^{1}H-NMR
(300 MHz, CDCl$_3$)
1.04
1.01
1.00
1.99
1.02
2.10
1.00
1.03
1.00
2.91
f1 (ppm)

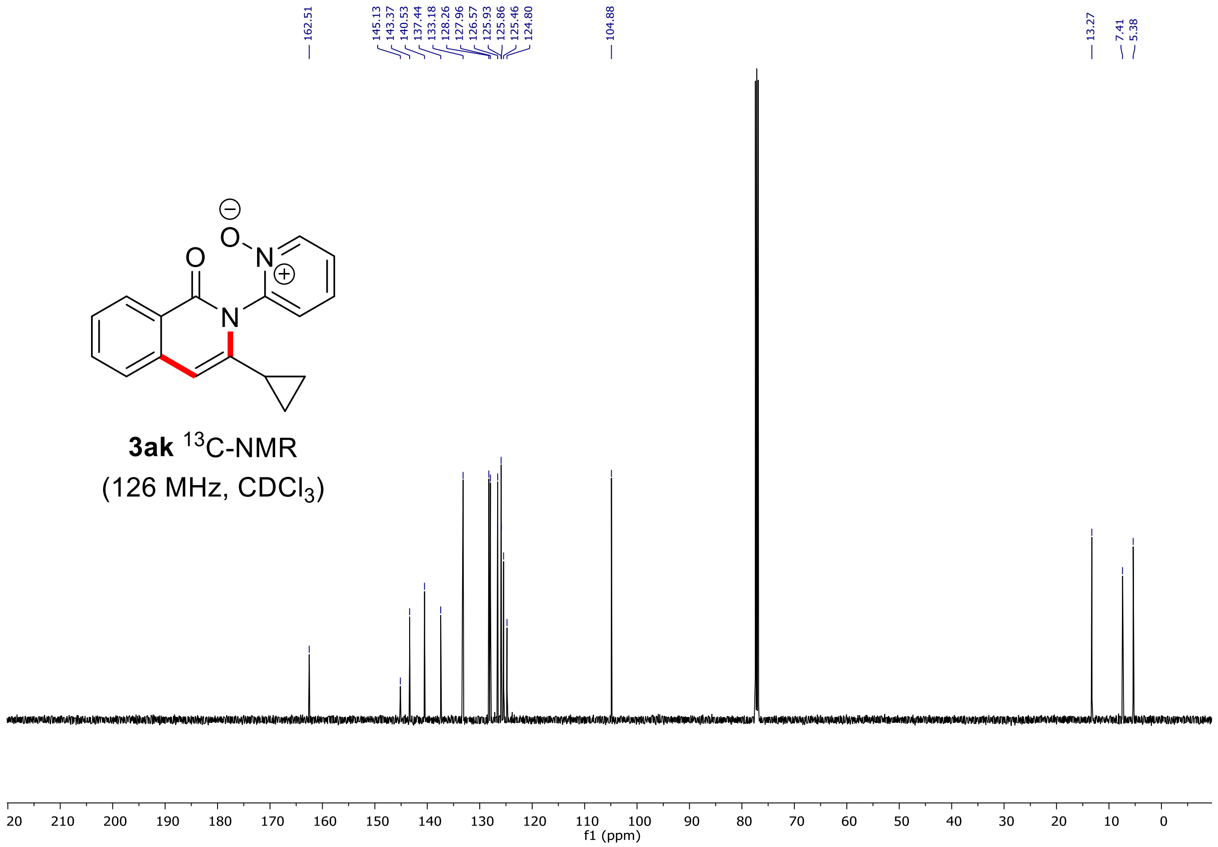

162.51
145.13
143.37
140.53
137.44
133.18
128.26
127.96
126.57
125.93
125.86
125.46
124.80
104.88
13.27
7.41
5.38
3ak ^{13}C-NMR
(126 MHz, CDCl$_3$)
f1 (ppm)

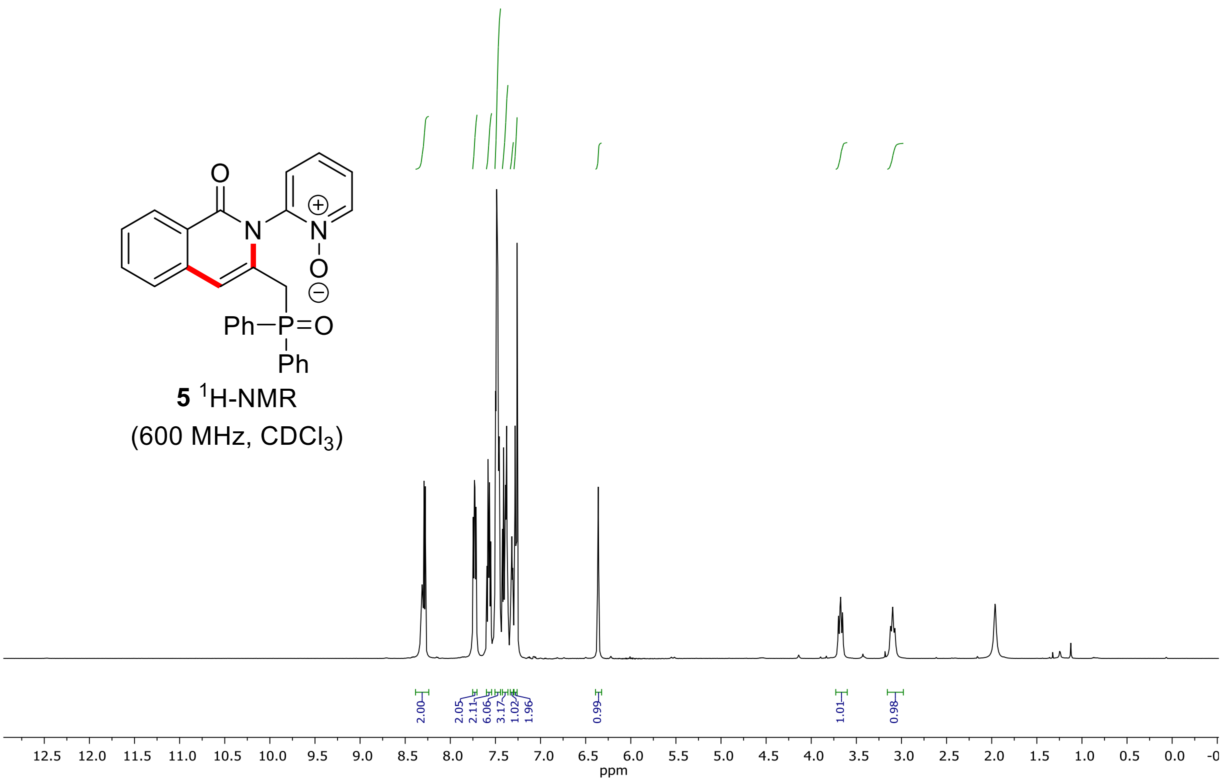
O
N
N
O
Ph−P=O
Ph
5 1H-NMR
(600 MHz, CDCl3)
2.00
2.05
2.11
6.06
3.17
1.02
1.96
0.99
1.01
0.98
12.5 12.0 11.5 11.0 10.5 10.0 9.5 9.0 8.5 8.0 7.5 7.0 6.5 6.0 5.5 5.0 4.5 4.0 3.5 3.0 2.5 2.0 1.5 1.0 0.5 0.0
ppm

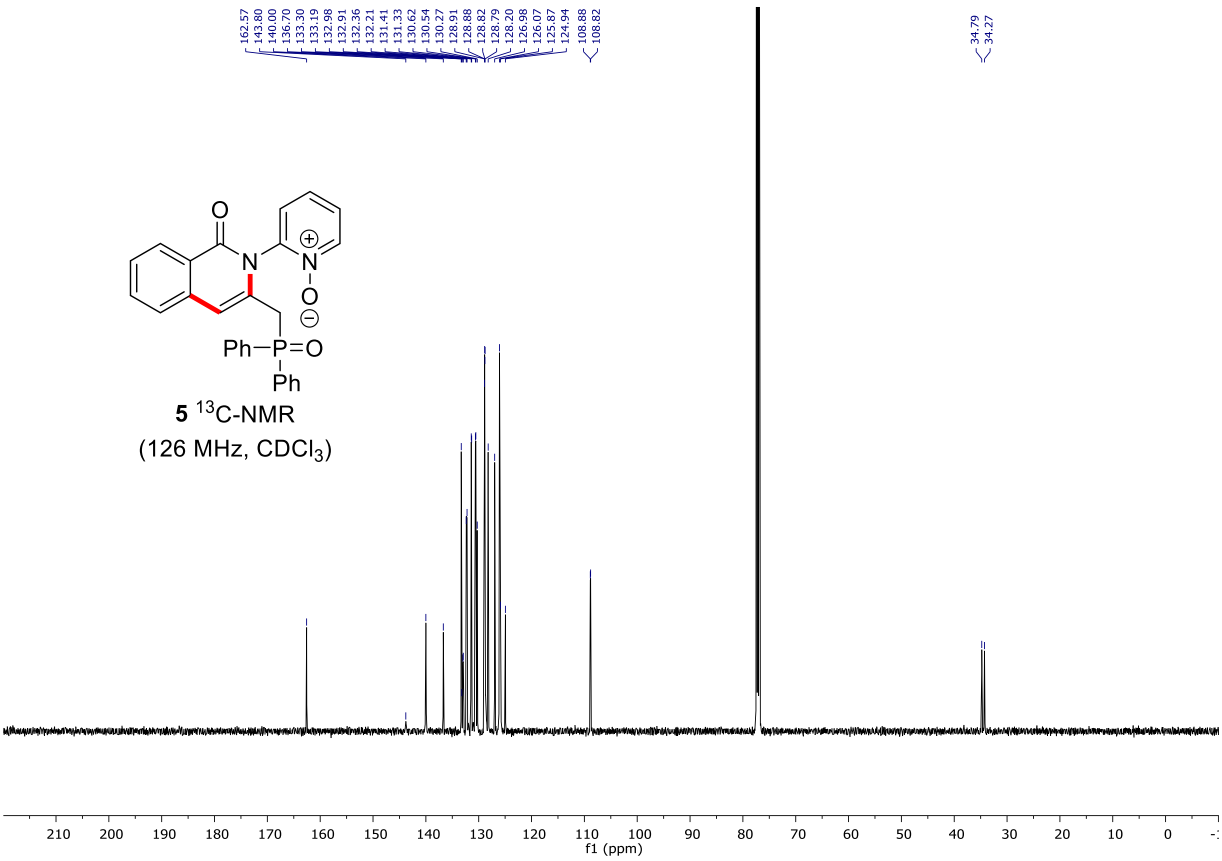
162.57
143.80
140.00
136.70
133.30
133.19
132.98
132.91
132.36
132.21
131.41
131.33
130.62
130.54
130.27
128.91
128.88
128.82
128.79
128.20
126.98
126.07
125.87
124.94
108.88
108.82
34.79
34.27
O
N
N
O
Ph−P=O
Ph
5 13C-NMR
(126 MHz, CDCl3)
210 200 190 180 170 160 150 140 130 120 110 100 90 80 70 60 50 40 30 20 10 0
f1 (ppm)

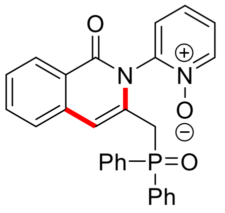

5 ^{31}P-NMR
(162 MHz, $CDCl_3$)

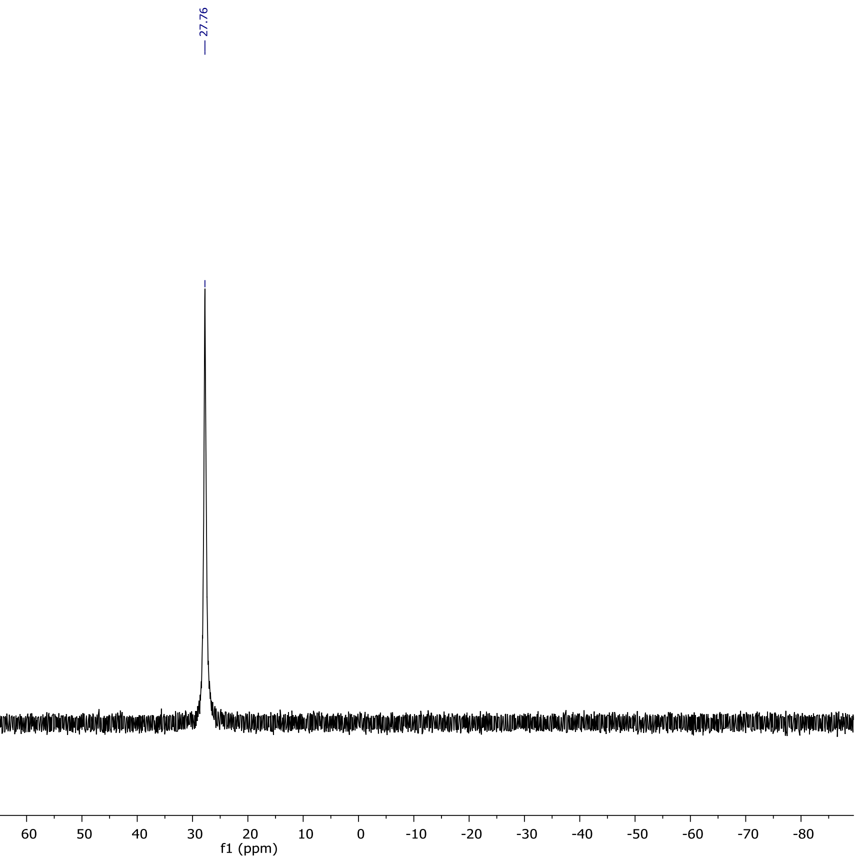

Supplement: Supplementary file 1 — Supplementary [file CSSC-13-668-s001.pdf]
